# Classification and distribution of functional groups of birds and mammals in Mexico

**DOI:** 10.1371/journal.pone.0287036

**Published:** 2023-11-07

**Authors:** Fernando Mayani-Parás, Claudia E. Moreno, Griselda Escalona-Segura, Francisco Botello, Mariana Munguía-Carrara, Víctor Sánchez-Cordero

**Affiliations:** 1 Departamento de Zoología, Instituto de Biología, Universidad Nacional Autónoma de México (UNAM), Mexico City, Mexico; 2 Posgrado en Ciencias Biológicas, Universidad Nacional Autónoma de México, Mexico City, Mexico; 3 Centro de Investigaciones Biológicas, Universidad Autónoma del Estado de Hidalgo, Pachuca, Hidalgo, Mexico; 4 Departamento de Conservación de la Biodiversidad, El Colegio de la Frontera Sur (ECOSUR), Campeche, Campeche, Mexico; 5 Comisión Nacional para el Conocimiento y Uso de la Biodiversidad (CONABIO), Mexico City, Mexico; Universidade Federal de Minas Gerais, BRAZIL

## Abstract

There has been a recent exponential growth in the study of functional trait ecology. Nonetheless, the study of functional traits and functional groups has been limited for terrestrial vertebrates. We conducted a classification update of functional groups (FG) of birds and mammals in Mexico, and determined the distribution patterns of FG species richness in different ecosystems nationwide. We selected six functional traits (feeding habit, locomotion, feeding substrate and technique, activity period, seasonality, and body size) obtained for 987 and 496 species of birds and mammals, respectively. A cophenetic correlation analyses resulted in values of 0.82 for the bird species dendrogram, and 0.79 for the mammal species dendrogram showing that the structures adequately reflected the similarity between observations. We obtained 52 FG for birds, assembled into 9 broader groups based on their feeding habits (16 invertivores, 6 carnivores: 5 herbivores, 9 aquatic vertivore/invertivore, 5 granivores, 1 scavenger, 3 nectarivores, 4 frugivores, and 3 omnivores). We obtained 35 FG for mammals, assembled into 9 broader groups based on their feeding habits (4 granivores, 10 herbivores, 1 nectarivore, 4 frugivores, 8 invertivores, 3 omnivores, 2 aquatic vertivore/invertivore, 1 hematophagous, and 2 carnivores). Overall, the distribution of FG species richness for birds and mammals gradually increased from the Nearctic to the Neotropical region, following a typical latitudinal species richness pattern. Few FG of migratory birds, and FG of granivore and herbivore mammals showed more species in the Nearctic and in the transitional regions. Our study provides a baseline for identifying ecological functions of species of birds and mammals in different ecosystems in Mexico, and contributes to understand the relationship between species diversity, community structure and ecosystem functioning. Identifying spatial patterns of functional trait diversity is important as biodiversity loss has a negative impact on ecosystem functioning and provision of environmental services.

## Introduction

Functional trait diversity has raised interest in biodiversity studies from multi-dimensional theoretical and practical perspectives that involves its relationship with species and phylogenetic diversities, as well as with ecosystem functioning and provision of ecosystem services [1–5]. On the theoretical side, incorporating information on species’ roles concerning species richness provides an integrated framework to address the relationship between diversities at the functional, species, and phylogenetic levels [6, 7]. On the practical side, relating spatial patterns of functional trait and species diversities is particularly important as biodiversity loss has a detrimental impact on ecosystem functioning and the provision of environmental services worldwide [2, 3]. Thus, producing information relating species and phylogenetic diversities with functional trait diversity contributes to understand the potential consequences of biodiversity loss on ecosystems [8].

Functional traits are morphological, physiological, anatomical, biochemical, and behavioral characteristics of individuals or species that directly or indirectly influence their role in the functioning of ecosystems [9–13]. By assigning functional traits, species can be classified into functional groups (FG) based on their ecological similarities, assuming that species of the same FG play similar roles in ecosystem functioning and processes [14, 15]. FG classification has helped to analyze the structure of communities and how they are influenced by the environment [16, 17], species interactions and anthropogenic impact [10, 18], assess how it modulates ecosystem processes and services [12, 19, 20] and to define sound strategies in conservation biology [21, 22].

The study of functional traits and FG has been widely applied in plants and other taxa [23–25]. However, it has been limited for terrestrial vertebrates, probably due to the difficulties in selecting and measuring their functional traits, the intensive sampling effort involved, and the evasive behavior of species [26]. Among the terrestrial vertebrates, birds have been the most studied (e.g., [27, 28]) given that measuring their functional traits is relatively straightforward; e.g., many species are abundant and occupy a high variety of ecological niches, and provide a wide range of ecosystem services [29]. For example, González-Salazar et al. [30] classified 1,502 species of North American birds and mammals (excluding marine, coastal and non-native species) into trophic guilds using three functional traits: feeding habit, feeding substrate and technique, and activity period. The combination of these traits resulted in a classification of 22 trophic guilds for birds and 27 for mammals, and each taxonomic group was assembled into eight broader groups based on their feeding habits [30]. This classification of functional traits has been used to study a wide range of topics as the composition of species, trophic guilds and trophic relationships in communities [31]; activity patterns and temporal variation of species and trophic groups [32]; ecological integrity of ecosystems [33]; impact of urbanization and fragmentation [34]; transmission of diseases or the presence of parasites related to ecological traits [35], and proposing restoration and conservation actions [36].

Here, we expanded the study by González-Salazar et al. [30] by adding species associated with coastal and freshwater ecosystems, as well as more functional traits, to produce a comprehensive classification of FG for species of birds and mammals. For example, an important distinction was that these authors used species activity period due to the temporal segregation that exists between species, allowing their coexistence despite using the same food resources [37, 38], but excluded species seasonality, which for birds is relevant for understanding the demand for trophic, spatial and temporal resources, especially between resident and migratory species. Here, we updated the FG classification of birds and mammals in Mexico and determined the distribution patterns of their species richness in different ecosystems nationwide. Specifically, we expected that adding more species and functional traits would increase the number of FG in birds and mammals compared to a previous classification. Further, given that species richness of birds and mammals is higher in the Neotropical region, we expected a higher species richness of FG in the tropical ecosystems.

## Materials and methods

### Functional traits and data collection

We selected six functional traits (feeding habit, locomotion, feeding substrate and technique, activity period, seasonality, and body size) given their importance on how species use food resources and their role in ecosystem function. The combination of these functional traits avoid species from being assigned to more than one group, and is comparable between both taxonomic groups. The selected functional traits were as follows:

#### Feeding habit

This trait includes all aspects related to species feeding behavior and the flow of matter and energy in ecosystems [27, 39]. Feeding habits of species were obtained by searching for quantitative information on the components of their diet. If a species consumed varied food items, we selected the feeding habit category based on the highest percentage of item consumed. If no quantitative data was available, the main food item reported in the literature was selected. We identified ten categories [40] shown in Table 1.

**Table 1:** List of functional traits and categories used to classify functional groups for birds and mammals in Mexico.

Functional trait	Category	Description	Number of bird species; number of mammal species
**Feeding habit**	Granivore	Main food item: seeds and grains	137; 133
	Herbivore	Main food item: vegetation (stems, shoots, leaves)	29; 112
	Frugivore	Main food item: fruits	67; 37
	Nectarivore	Main food item: nectar	62; 14
	Invertivore	Main food item: invertebrates	464; 160
	Carnivore	Main food item: vertebrates	55; 15
	Scavenger	Main food item: dead animals	6; 0
	Aquatic vertivore/invertivore	Main food item: aquatic invertebrates and vertebrates	137; 8
	Omnivore	Main food item: plants and animals	30; 14
	Hematophagous	Main food item: vertebrate blood	0; 3
**Locomotion**	Terrestrial	Species live and carry out their activities predominantly or entirely on the ground surface	216; 221
	Fossorial	Species adapted to excavate, that live and carry out their activities underground, digging tunnels and burrows	0; 3
	Semi-fossorial	Species adapted to excavate, that live and carry out their activities underground and go to the surface during some hours of the day	0; 62
	Arboreal	Species carry out their activities on trees and live in them most or all of their lives	294; 14
	Semi-arboreal	Species with adaptations for climbing, that carry out their activities on trees, but spend a similar time on the surface of the earth	64; 44
	Semi-aquatic	Species with an affinity for the aquatic environment but must spend part of the day out of the water.	158; 6
	Volant	Species move and carry out their activities in the air, either by flying or gliding	225; 146
**Feeding substrate and technique**	Ground gleaner/ browser/grazer	Species get their food on the ground	148; 205
	Underground browser	Species get their food underground	0; 21
	Ground hunter	Species hunt their prey on the ground	162; 84
	Scavenger	Species feed on carrion	6; 0
	Underground hunter	Species hunt their prey underground	0; 3
	Arboreal gleaner/ browser	Species get their food from trees	155; 79
	Arboreal hunter	Species hunt their prey on trees	239; 12
	Aquatic surface gleaner/ browser	Species hunt their prey on the surface of freshwater bodies	22; 1
	Aquatic surface hunter	Species capture their food on the surface of water bodies	83; 8
	Wader	Species capture their food (aquatic invertebrates) in the mud or sand	54; 0
	Air hunter above canopy	Species hunt their prey in the air above the tree canopy	27; 11
	Air hunter under canopy	Species hunt their prey in the air under the tree canopy	91; 72
**Activity period**	Diurnal	Activity period begins mainly in the morning and continuous during the day	921; 46
	Nocturnal	Activity period begins in the late afternoon and continues through the night.	37; 393
	Cathemeral	Species carry out their activities both during the day and at night	29; 57
**Seasonality (birds)**	Resident	Species present in the country throughout the year	661; 496
	Migrant	Species are only observed in Mexico during some months of the year and migrate north or south the other part of the year	288; 0
	Transient	Species are observed when passing through their migratory route to Central and/or South America	24; 0
	Accidental	Species of which only some individuals are observed sporadically and may be outside their distribution ranges	14, 0
**Body size**	Continuous variable (g)		

#### Locomotion

This functional trait is related to the morphology of a species, its spatial use of resource and habitat adaptations for foraging and refuge [29]. We identified seven categories (Table 1) [41], and excluded the “aquatic” category because no marine species were included in our analyses.

#### Feeding substrate and technique

This functional trait is related to the strategy that a species uses to obtain food, and the area conducting feeding activities. This functional trait reflects how a species is distributed in microhabitats, and how it impacts the use of resources, and the flow of matter and energy in ecosystems [27, 39]. We considered four types of microhabitats (terrestrial, arboreal, aerial and aquatic). These microhabitats were subdivided according to the sites where species predominantly conduct their feeding activities, and identified twelve categories (Table 1) [40]. The category “aquatic dive hunter” was excluded as marine species were not included.

#### Activity period

This functional trait includes the period of the day in which a species conducts its main feeding activity. It is related to species life history and to predator-prey interactions [29, 37, 38]. We identified three categories (Table 1) [40].

#### Seasonality

This functional trait was selected only for birds due to the migratory behavior of some species that remain for months in Mexico. Its ecological importance lies on the demand for trophic, spatial and temporal resources, and in the temporal separation over resource use, and the impact on nutrient cycling and services across broad regions [27, 29]. We identified five categories (Table 1) [40].

#### Body size (g)

This functional trait is associated with species life history, including its metabolic rate, physiology, longevity, demand for trophic resources, energy flow between trophic levels in the ecosystem, feeding behavior, and home range size, among other biological characteristics [27, 29, 39]. This functional trait was considered as a continuous variable.

We built a database including all functional traits reported above (S1 and S2 Tables). The information on functional traits was obtained from the previously published compilation of specialized literature for ecological characteristics for birds and mammals provided by CONABIO (see [40]) and complemented using extensive databases (PanTHERIA; https://animaldiversity.org/; https://birdsoftheworld.org; https://avibase.bsc-eoc.org/); when trait information was not available, we either used genus/family values or our own expertise. The taxonomy was reviewed and updated for continental, freshwater and coastal species [42, 43]. A total of 987 species of birds and 496 species of mammals were included in the study.

### Classification of species into functional groups

We conducted the clustering and analyses of species FG using R software [44]. The functional traits database was converted into a species (S) vs functional traits (t) matrix, which was subsequently converted into a dissimilarity matrix using the Gower’s dissimilarity measure [45], given the use of categorical and continuous variables. We assigned higher weights to the feeding habit and body size traits because (1) the feeding habit trait reflects an important role of species in ecosystem function [46], and (2) the body size trait is closely related to life-history (particularly in mammals) which involves many biological attributes of species. Moreover, combining categorical and continuous traits can result in underestimating the important biological role of the latter traits [47]. Unfortunately, no algorithm exists to assign weights objectively [47]. Thus, we assigned a weight of 0.5 to the functional traits of feeding habits and body size, and of 0.3 to the remaining functional traits.

FG were clustered in a dendrogram using Ward hierarchical clustering algorithm [48]. The dendrogram performance was analyzed with a cophenetic correlation analysis (Pearson’s correlation analysis) between the distances of the dendrogram and the dissimilarity matrix. The elbow method in the NbClust package [49] was used to determine the optimal number of clusters using 30 different indices. The resulting number of FG for birds and mammals were 2. Thus, we decided to cut the dendrogram manually at different arbitrary heights to compare the resulting FG.

### Species distribution maps and patterns of species richness of functional groups

To identify geographic patterns of species richness of FG, we followed a two-step protocol. First, species distributions were obtained from CONABIO´s geoinformation portal (http://www.conabio.gob.mx/informacion/gis/; last accessed on July 2022). These distribution maps were produced by specialists on the avian and mammalian fauna of Mexico. Overall, the species distribution maps from CONABIO followed established modeling protocols, including taxonomic revision of species and georeferenced point localities throughout their distribution range. Ecological niche models projected as species potential distributions were generated in R software [42] with the ENMeval library [50]. The modeling area for each species (M region [51]) was obtained by selecting the polygons of the terrestrial ecoregions of Mexico [52] containing species occurrence data [53–55], and including a buffer zone of 50 km around the polygons used as a cutting template. Species potential distributions were constructed using 19 climatic variables (∼1 km^2^) from the WorldClim database (https://www.worldclim.org/; accessed between 2013 and 2018 [56]), which describe temperature and precipitation values represented in monthly (e.g., precipitation of driest month), quarterly (e.g., mean temperature of coldest quarter), seasonal (e.g., temperature seasonality), and annual trends (e.g., annual precipitation). A correlation analysis was performed to include in the model only one variable when variables had a correlation threshold > 0.7 [57]. Ten thousand background points were selected to parameterize the model within the modeling area, and presence data were divided into training and testing groups using the block method [58]. The best model was selected based on the omission rate and area under the curve (AUC), thus measuring the likelihood that a randomly selected presence point is located in a raster cell, with a higher probability value for species occurrence than a randomly selected absence point. The best model for each species was projected into a presence/absence map using a maximum sensitivity plus specificity threshold, which includes the most occurrences in the smallest possible area, representing the species potential distribution [59].

For birds, we included 790 distribution maps (http://www.conabio.gob.mx/informacion/gis/; last accessed on July 2022), of which 757 and 33 maps were produced by Navarro-Sigüenza and Gordillo-Martínez [60] and Navarro-Sigüenza and Peterson [61], respectively. We excluded 171 shore and freshwater bird species because of a lack of reliable maps, and an additional 26 bird species because they occur only on islands. For terrestrial mammals, we included 433 distribution maps (http://www.conabio.gob.mx/informacion/gis/; last accessed on July 2022), of which 338 were obtained from Sánchez-Cordero et al. [62], 27 maps from Sánchez-Cordero et al. [63], 19 maps from Briones-Salas et al. [64], 10 maps from Farías et al. [65],, 5 maps from Ballesteros-Barrera et al. [66], and 34 maps from this study. We excluded 63 species of mammals because they occurred on islands or lacked a distribution map.

Second, the distribution maps of species belonging to the same FG were overlapped to obtain a species richness map of each FG using a 0.5° grid, with QGIS software [67]. We generated FG species richness maps for birds (considering the non-migratory season and the migratory season) and mammals. We assigned 10 feeding categories for birds and mammal species, of which invertivores and granivores had the highest number of species for both birds (464 and 137 species, respectively) and mammals (160 and 133 species, respectively) (Tables 1 and S1 and S2).

## Results

### Dendrograms of birds and mammals

A cophenetic correlation analyses resulted in values of 0.82 for the bird species dendrogram, and 0.79 for the mammal species dendrogram showing that the structures of both analyzes adequately reflected the similarity between observations. The cut of the dendrogram was established arbitrarily at a similarity height of 0.5 and 0.3 (Gower distance); a 0.5 cutoff resulted in 38 FG for birds and 23 FG for mammals, while a 0.3 cutoff resulted in 52 FG for birds and 35 FG for mammals. The difference was mainly due to the separation of the species activity period and seasonality into narrower groups, when the cut was established at 0.3. We decided to use these groups, which explained in more detail the ecological role of species.

### Functional groups in birds

**Fig 1:**
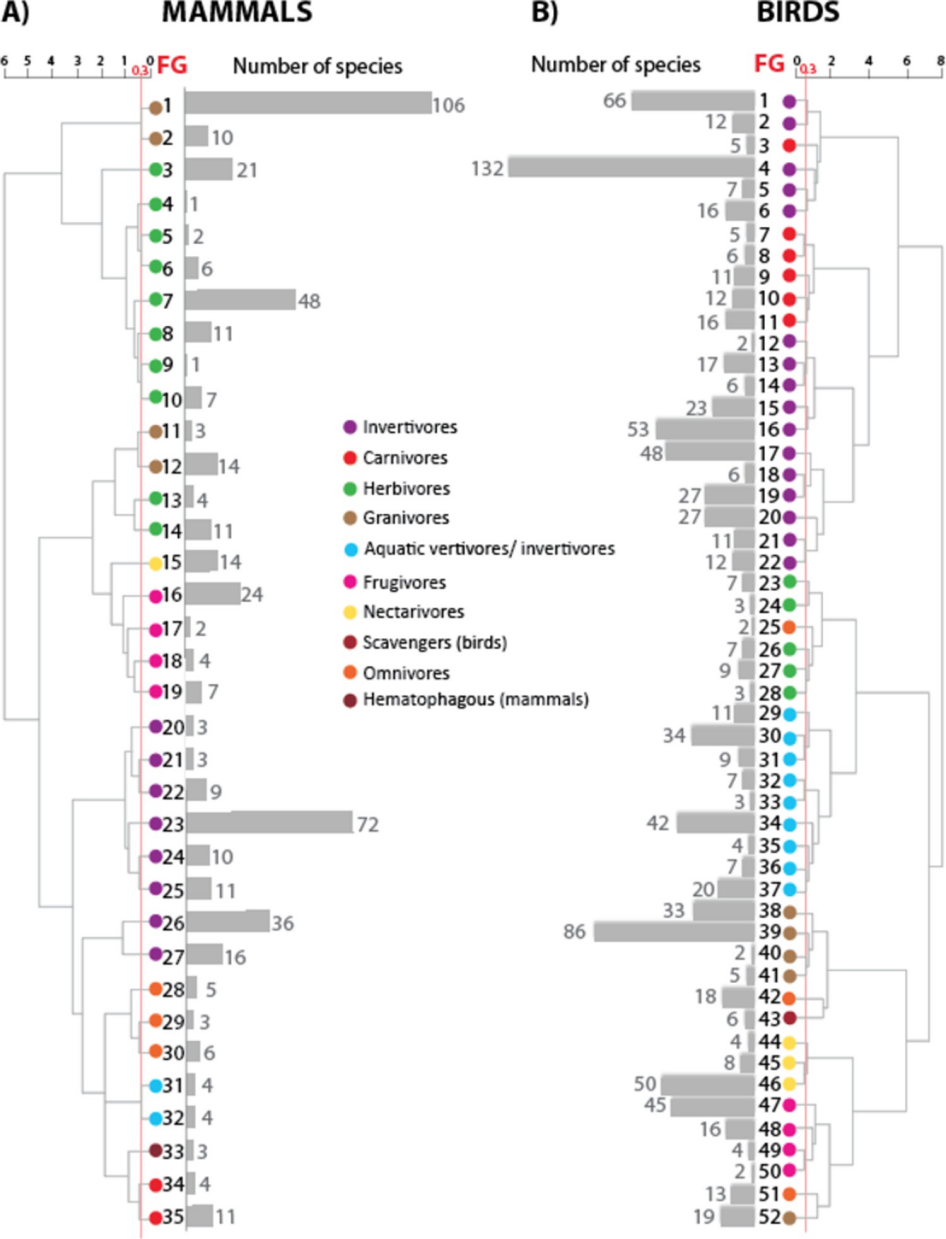
Functional groups (FG) for species of birds and mammals occurring in Mexico. Dendrogram and FG obtained for (A) 496 species of mammals and (B) 987 species of birds. Number in bars indicate the number of species in each FG. Color legends indicate the broader groups based on their feeding habits (invertivores, carnivores, herbivores, granivores, aquatic vertivores/invertivores, frugivores, nectarivores, scavengers [birds], omnivores, hematophagous [mammals]).

We obtained 52 FG for birds (Fig 1B and S1 Table and S1 Text) which were assembled into 9 broader groups based on their feeding habits (invertivores: 16 FG; carnivores: 6 FG; herbivores: 5 FG; aquatic vertivore/invertivore: 9 FG; granivores: 5 FG; scavengers: 1 FG; nectarivores: 3 FG; frugivores: 4 FG; omnivores: 3 FG). The number of each FG was assigned according to its appearance in the dendrogram. The distribution map of each functional group is shown in S1 File.

The FG with the highest number of species were: (1) FG4: invertivores, arboreal hunter, resident (132 species); (2) FG39: granivores, ground gleaner, resident (86 species); (3) FG1: invertivores, arboreal hunter, migrant (66 species); (4) FG16 invertivores, ground hunter, resident (53 species), and (5) FG46: nectarivores, volant, resident (50 species). The functional groups with the lowest number of species were: (1) FG12: invertivores, semi-aquatic hunter (1 species); (2) FG25: omnivores, semi-aquatic gleaner (2 species); (3) FG40: granivores, semi-aquatic gleaner (2 species); (4) FG50: frugivores, semi-arboreal, ground gleaner (2 species) (Fig 1B and S1 Table).

**Fig 2:**
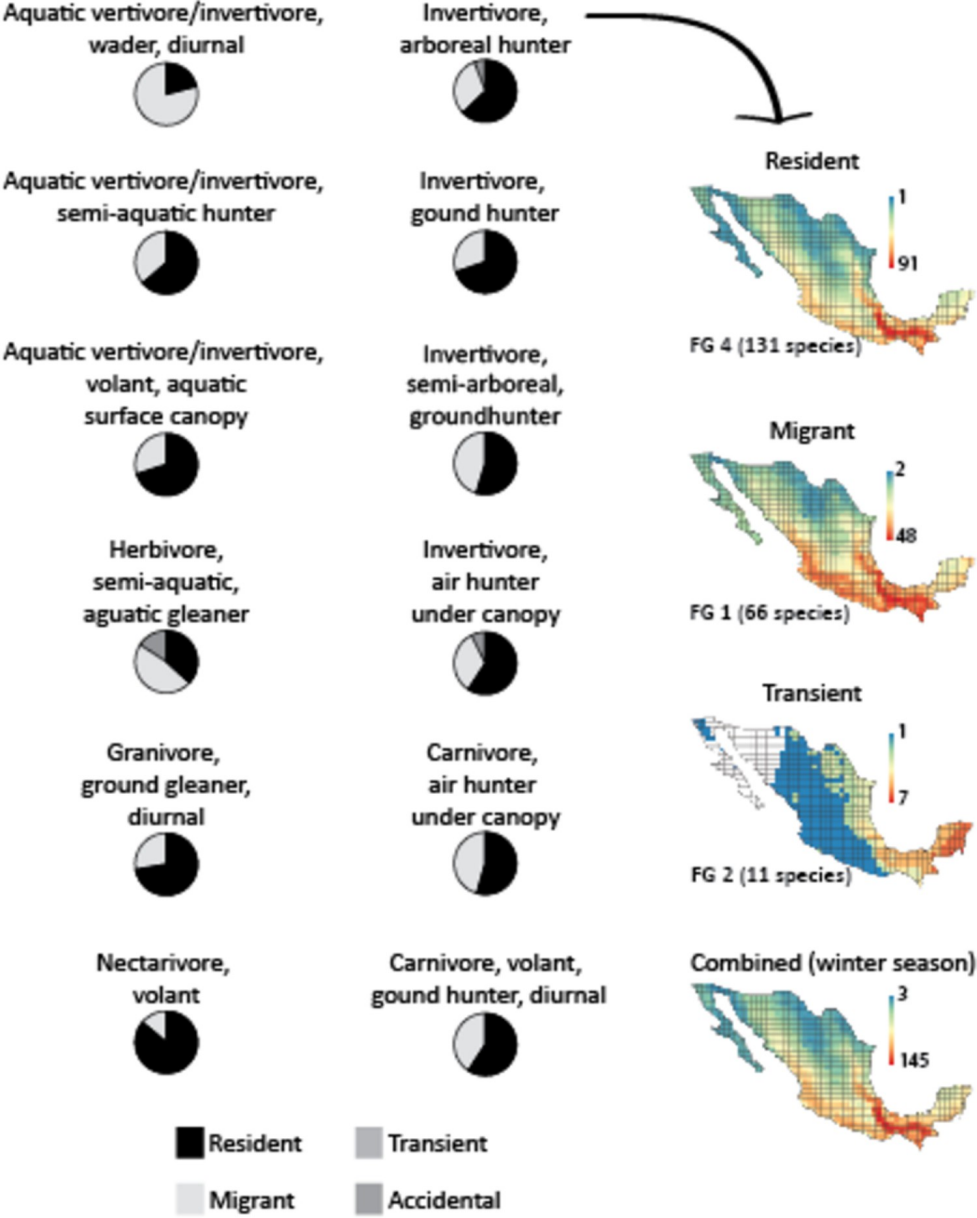
FG of birds divided by seasonality depicting the contribution of resident, migrants, transient and accidental species, with an example of how one FG (invertivore, arboreal hunter) is distributed in Mexico and the number of species found along the country, adding the combined map for the winter season when all species coincide in Mexico.

There was 12 broader FG of species that occupied the same trophic niche but were divided by seasonality, separating resident, migratory, transient, and accidental species into different groups (Fig 2 and S1 File). However, in some months of the year (mainly in winter), they all occur in Mexico using the same resources. Most of these 12 broader FG are dominated by resident species, except for some semi-aquatic species, mainly migrants (Fig 2). In the migratory season, the FG of “invertivores, air hunters under canopy” and the “aquatic vertivore/invertivore, semi-aquatic hunters” became two of the functional groups with more species (81 and 66, respectively).

### Functional groups in mammals

We obtained 35 FG for mammals (Fig 1A and S2 Table and S1 Text) which were assembled into nine broader groups based on their feeding habit (granivores: 4 FG; herbivores: 10 FG; nectarivores: 1 FG; frugivores: 4 FG; invertivores: 8 FG; omnivores: 3 FG; aquatic vertivore/invertivore: 2 FG; hematophagous: 1 FG; carnivores: 2 FG). The number for each FG was assigned according to its appearance in the dendrogram. The distribution maps for each FG are shown in S1 File.

**Fig 3:**
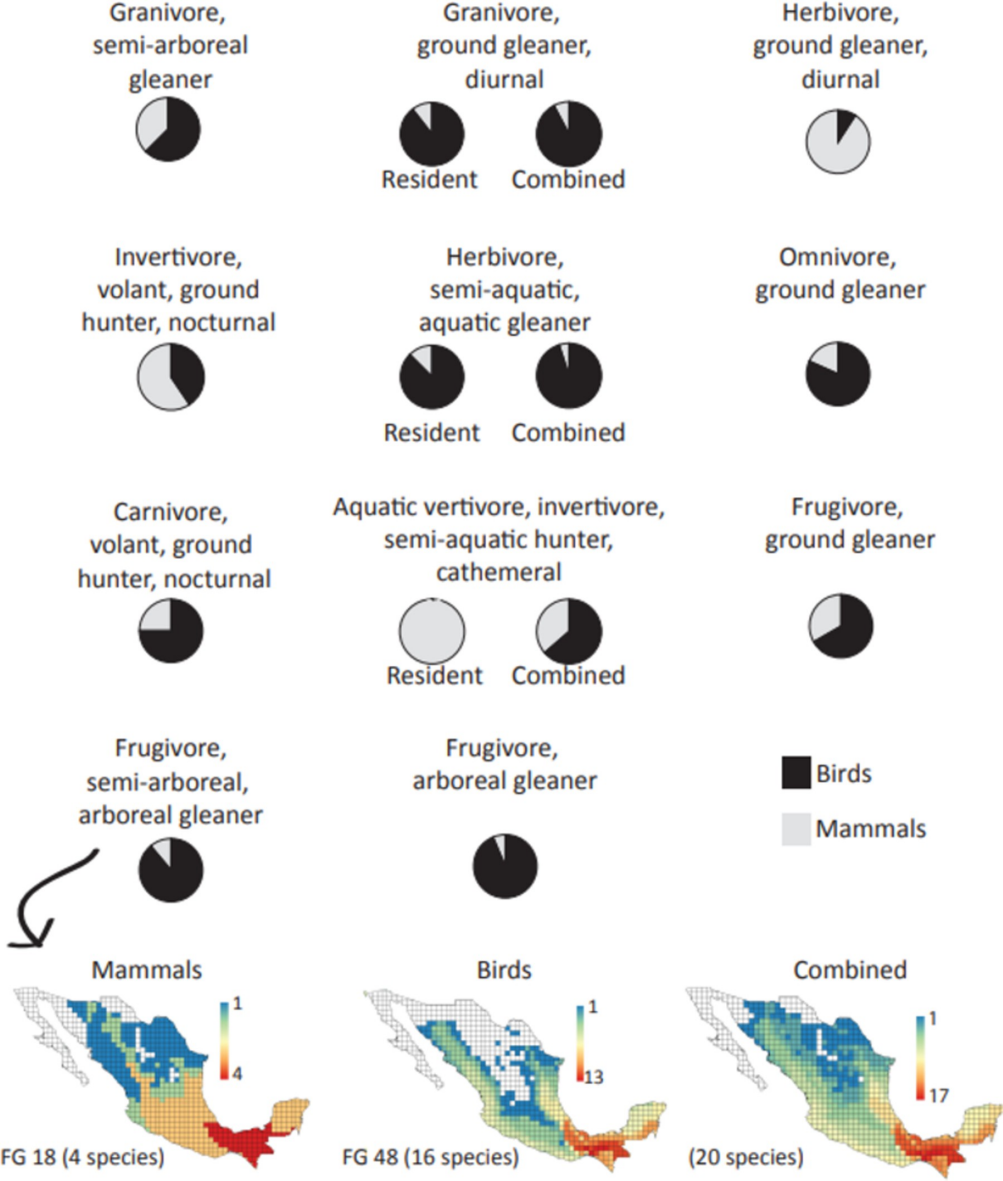
FG shared by species of mammals and birds, and the contribution of each taxonomic group, with an example of how one group (frugivore, semi-arboreal, arboreal gleaner) is distributed in Mexico when considering only mammalian species, only bird species, and both taxonomic groups combined.

The FG with the highest number of species were: (1) FG1: granivores, ground browser, nocturnal (106 species); (2) FG23: invertivores, air hunter under canopy (72 species); (3) FG7: herbivores, ground browser/grazer, nocturnal (48 species); (4) FG26: invertivores, semi-fossorial hunter (36 species); and (5) FG16: frugivores, aerial browser (24 species). In contrast the FG with the lowest number of species were: (1) FG4: herbivores, semi-aquatic browser (1 species); (2) FG9: herbivores, ground browser/grazer, large (1 species); and (3) FG5: herbivores, semi-arboreal, ground browser (2 species) (Fig 1A and S2 Table). Further, birds and mammals shared eleven FG; eight FG were dominated by bird species, two by mammal species, and one (aquatic vertivore/invertivore, semi-aquatic hunter, cathemeral) was dominated by mammals in the non-migratory season, and by birds in the migratory season (Fig 3).

### Distribution of functional groups

**Fig 4:**
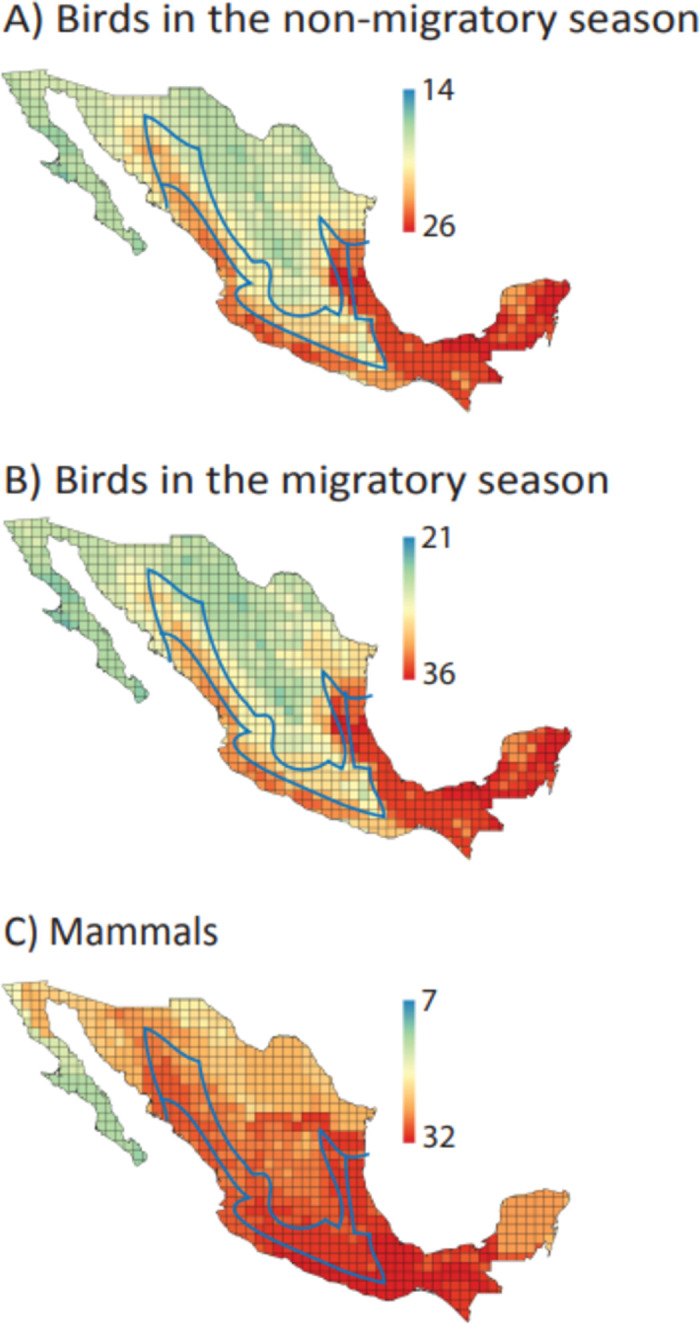
Functional group richness (number of functional groups) across Mexico, for (A) birds in the non-migratory season, (B) birds in the migratory season and (C) mammals. Blue polygon represents the Mexican Transition Zone, between the Nearctic region (north) and the Neotropical region (south) [61].

The distribution of species richness for most FG of birds and mammals followed the typical latitudinal pattern with a gradual increase from the Nearctic to the Neotropical region (S1 File). Only few FG of migratory birds (FG 7, FG 9, FG 15 and FG 38) and other resident species (FG 41, FG 42 and FG 52) showed different distribution patterns; either with more species occurring in the Nearctic region or a homogeneous species richness nationwide. For mammals, some FG of granivores and herbivores showed more species in the Nearctic and in the transitional regions [68], respectively (S1 File). For the 11 FG shared by birds and mammals, their species richness mostly showed a latitudinal pattern (Fig 1F and S1 File). Overall, FG species richness for birds in the non-migratory season and birds in the migratory season showed more species in the Neotropical region (Fig 4A and 4B). For mammals, FG species richness was higher in the Neotropical and transitional regions (Fig 4C).

## Discussion

### Functional group classification

Studies related to functional trait ecology in Mexico have been limited and primarily focused on restricted groups of species and regions [69, 70], rather than including entire taxonomic groups and national coverage (but see [18, 26]). González-Salazar et al. [30, 40] proposed a classification of FG of an important fraction of species of birds and mammals occurring in Mexico. Our study extended their proposal by including a complete list of species of these taxonomic groups occurring in Mexico, adding coastal and freshwater species, such as shorebirds, which represent one of the most abundant group of species [71]. Furthermore, we rearranged FG to include locomotion, seasonality and body size to adequately categorize species according to their trophic function and ecological traits, such as the use of food resources, how species move to obtain their food, habitat use, the flow of matter and energy in ecosystems, and spatiotemporal segregation. Although we recognize that many other functional traits influence the role of species in ecosystems and have been used in previous studies (e.g., activity patterns, social structure, behavioral plasticity; see [72, 73]), it is not clear if they are robust predictors of species ecological conditions [14].

The inclusion of more species and traits increased the number of FG compared to those proposed by González-Salazar et al. [30], resulting in 52 groups of birds (compared to 22) and 35 groups of mammals (compared to 27) that were classified in broader FG according to their feeding habits (Fig 1 and S1 and S2 Tables). For example, by adding coastal species and those with freshwater habits, nine FG of bird species feeding on aquatic vertebrates and invertebrates ("aquatic vertivore/invertivore") were included. Further, by including seasonality as a trait, we were able to separate species exploiting similar food resources into independent groups according to the season they are present in the country. Seasonality is a relevant trait causing changes in the demand for trophic resources year-round due to the temporal segregation and nutrient flow in ecosystems [37–39]. Due to the geographic scale of the study, we considered seasonality as only migrations referring to populations moving outside Mexico for part of the year, and excluding local, altitudinal and migrations where only a single sex migrates. In the case of mammals, seasonality was not included.

Body size was a functional trait that only allowed to differentiate FG of large species on two occasions: with the American white pelican (*Pelecanus erythrorhynchos*) and three other bird species separated from the rest of the “aquatic vertivore/invertivore, semi-aquatic hunter, diurnal”, and the American bison (*Bison bison*) being separated from the “herbivores, ground browser/grazer, diurnal”. One explanation is that when combining categorical and quantitative traits, the important biological role of continuous traits can be underestimated [47]. Furthermore, body size is frequently used in studies of functional ecology due to its relationship with many ecological traits [18, 74]. Body size can show a limited accuracy in explaining species trophic niches, trophic levels or foraging niches when included as the only morphological measure. On the other hand, predictability increases when body size is considered with other morphological traits (such as beak size and shape, for birds) [75]. It is possible that the influence of body size in species clustering could be higher when adding other morphological traits.

Further, closely related species likely have similar functional traits, ecological niches, and interactions because of shared ancestry [76, 77]. While some functional traits show strong phylogenetic signals (the tendency for closely related species to resemble each other more than less related taxa), others are more or less conservative [78]. This suggests that functional traits and trophic niches may have evolved multiple times, showing phenotypic convergence towards the same adaptive optimum [79]. Although species of the same family formed some FG (e.g. Birds- FG49: Frugivores, ground gleaner, Family: Tinamidae; Mammals- FG3: Herbivores, semi fossorial, underground browser, Family: Geomydae; S1 and S2 Tables), a high number of FG were formed by multiple distantly related clades that are closer in the functional space than expected based on their evolutionary relatedness (e.g. Birds- FG39: Granivores, ground gleaner, resident, Families: Odontophoridae, Phasianidae, Columbidae, Alaudidae, Estrildidae, Calcariidae, Emberizidae, Cardinalidae, Icteridae, Fringillidae; Mammals- FG10: Herbivores, ground browser/grazer, diurnal, Families: Cricetidae, Sciuridae, Antilocapridae, Bovidae, Tayassuidae; S1 and S2 Tables), suggesting that the correspondence between traits and function requires an adaptive explanation. Furthermore, we observed a convergence between 11 FG of birds and mammals (Fig 3), suggesting that some functional niches are exclusive for birds and mammals (54.5% and 48.5%, respectively), while other functional niches can be occupied by both taxonomic groups increasing resource competition. It would be interesting to test if this macroevolutionary convergence increases if other terrestrial vertebrates, such as reptiles and amphibians, were included in these analyses [76, 78, 79].

### Distribution of functional groups

Most FG showed higher species richness in the Neotropical region, following the general pattern of species richness increasing towards the tropics [80] (S1 File). This also follows the species richness distribution of birds and mammals in Mexico, where the habitats holding the highest species richness include tropical humid and dry forests, and humid highlands [81, 82]. Only few FG had higher species richness in the Mexican Transvolcanic Belt, a region known as a transition between the Nearctic and Neotropical regions [68]. This region is a topographic and climatic complex area where species from different biogeographical origins co-occur, showing high rates of speciation and endemism, particularly for mammals [83]. Thus, we can expect a high FG species richness in this region for mammals with high number of endemic species with restricted distributions, such as “mammals-herbivores-semi fossorial-underground browsers”.

Some FG of mammals showed a more heterogenous species richness nationwide. In fact, some FG showed a higher species richness in the Nearctic region as a consequence of the high number of small and medium-sized species associated with grasslands, scrublands and arid ecosystems, where they perform important ecological functions, such as seed dispersal, pollination, herbivory, and predator-prey species interactions [46]. Further, representing the temporal segregation of FG in a geographic space helps to understand macroevolutionary convergence, which could be explained by species adaptation to vacant ecological niches [84]. For example, the seasonality of FG allows to understand the changes in demands for trophic resources year-round. Interestingly, some FG in birds separated by seasonality (e.g., invertivores-ground hunter) showed different distribution patterns, with resident and migrant species having predominantly a Neotropical and Nearctic distribution, respectively. Also, temporal differences in the activity period between birds (mainly diurnal) and mammals (mainly nocturnal) facilitates exploiting same resources in the same area avoiding species interference.

### Final remarks

We acknowledge that our FG classification could have limitations, given the criteria that were established when grouping species, as excluding the functional differences that could exist between species of the same FG [14]. Furthermore, a main challenge in functional ecology is managing the intraspecific variation of functional traits [85]. When considering categorical traits, species must be assigned to a single category even if functional traits vary between individuals [86]. Species could belong to more than one category depending, for example, on the temporal and geographic variations that could exist between populations [30], the changes in the species life stage between juveniles and adults [87], or the phenotypic plasticity related to biotic and abiotic environmental factors [88]. Although we included a wide range of ecological traits, their categorical nature is likely to confound observed patterns that could be revealed by functional traits with continuous measurements [89], which can predict subtler fine-scale variations in dietary and behavioral niches than can be achieved by using standard niche categories [75].

Nonetheless, our study provides a solid baseline for identifying ecological functions of species of birds and mammals in different ecosystems in Mexico, allowing a better understanding of the relationship between species diversity, community structure and ecosystem functioning. Our proposal identified distribution patterns of FG species richness for birds and mammals nationwide, providing a framework for management and conservation actions. This is relevant as FG are useful for quantifying ecological redundancy and ecosystem resilience [90, 91]. To identify spatial patterns of functional trait diversity is particularly important as biodiversity loss has a negative impact on ecosystem functioning and provision of environmental services [3]. There is a need to quantitatively address the underlying assumption that loss of functional trait diversity (as a dimension of biodiversity) is directly related to a loss of ecosystem functioning and provision of environmental services [3].

## Supporting information

S1 Table Functional traits for 987 bird species distributed in Mexico and the functional groups assigned to them. (PDF)

S2 Table Functional traits for 496 mammalian species distributed in Mexico and the functional groups assigned to them. (PDF)

S1 Text Description of each functional group of birds and mammals in Mexico. (DOCX)

S1 File Species richness map of each functional group of birds and mammals in Mexico. (DOCX)
