# Supplementary material for: Classification and distribution of functional groups of birds and mammals in Mexico
Source: PLoS One. 2023 Nov 7;18(11):e0287036. doi: 10.1371/journal.pone.0287036 (PMC10629651; doi:10.1371/journal.pone.0287036)
Supplement: S1 Table — (PDF) [file pone.0287036.s001.pdf]

S1 Table. Functional traits for 987 bird species distributed in Mexico and the Functional Groups assigned to them.

Functional group	Species	Order	Family	Feeding habit	Locomotion	Feeding substrate and technique	Activity period	Seasonality	Body size (g)
1	*Coccyzus americanus*	Cuculiformes	Cuculidae	Insectivore	Arboreal	Arboreal hunter	Diurnal	Winter migrant	60.7
1	*Sphyrapicus thyroideus*	Piciformes	Picidae	Insectivore	Arboreal	Arboreal hunter	Diurnal	Winter migrant	47.6
1	*Sphyrapicus varius*	Piciformes	Picidae	Insectivore	Arboreal	Arboreal hunter	Diurnal	Winter migrant	50.3
1	*Sphyrapicus nuchalis*	Piciformes	Picidae	Insectivore	Arboreal	Arboreal hunter	Diurnal	Winter migrant	50.3
1	*Sphyrapicus ruber*	Piciformes	Picidae	Insectivore	Arboreal	Arboreal hunter	Diurnal	Winter migrant	48.9
1	*Vireo griseus*	Passeriformes	Vireonidae	Insectivore	Arboreal	Arboreal hunter	Diurnal	Winter migrant	11.4
1	*Vireo bellii*	Passeriformes	Vireonidae	Insectivore	Arboreal	Arboreal hunter	Diurnal	Winter migrant	8.5
1	*Vireo atricapilla*	Passeriformes	Vireonidae	Insectivore	Arboreal	Arboreal hunter	Diurnal	Winter migrant	9
1	*Vireo vicinior*	Passeriformes	Vireonidae	Insectivore	Arboreal	Arboreal hunter	Diurnal	Winter migrant	12.87
1	*Vireo flavifrons*	Passeriformes	Vireonidae	Insectivore	Arboreal	Arboreal hunter	Diurnal	Winter migrant	18
1	*Vireo plumbeus*	Passeriformes	Vireonidae	Insectivore	Arboreal	Arboreal hunter	Diurnal	Winter migrant	16.4
1	*Vireo cassinii*	Passeriformes	Vireonidae	Insectivore	Arboreal	Arboreal hunter	Diurnal	Winter migrant	14.7
1	*Vireo solitarius*	Passeriformes	Vireonidae	Insectivore	Arboreal	Arboreal hunter	Diurnal	Winter migrant	16.6
1	*Vireo gilvus*	Passeriformes	Vireonidae	Insectivore	Arboreal	Arboreal hunter	Diurnal	Winter migrant	14.8
1	*Vireo philadelphicus*	Passeriformes	Vireonidae	Insectivore	Arboreal	Arboreal hunter	Diurnal	Winter migrant	12.2
1	*Vireo flavoviridis*	Passeriformes	Vireonidae	Insectivore	Arboreal	Arboreal hunter	Diurnal	Winter migrant	18
1	*Vireo altiloquus*	Passeriformes	Vireonidae	Insectivore	Arboreal	Arboreal hunter	Diurnal	Winter migrant	18
1	*Certhia americana*	Passeriformes	Certhiidae	Insectivore	Arboreal	Arboreal hunter	Diurnal	Winter migrant	8.175
1	*Polioptila caerulea*	Passeriformes	Polioptilidae	Insectivore	Arboreal	Arboreal hunter	Diurnal	Winter migrant	6
1	*Corthylio calendula*	Passeriformes	Regulidae	Insectivore	Arboreal	Arboreal hunter	Diurnal	Winter migrant	6.65
1	*Catharus ustulatus*	Passeriformes	Turdidae	Insectivore	Arboreal	Arboreal hunter	Diurnal	Winter migrant	30.3
1	*Dumetella carolinensis*	Passeriformes	Mimidae	Insectivore	Arboreal	Arboreal hunter	Diurnal	Winter migrant	32.15
1	*Helmitheros vermivorum*	Passeriformes	Parulidae	Insectivore	Arboreal	Arboreal hunter	Diurnal	Winter migrant	13.2
1	*Vermivora chrysoptera*	Passeriformes	Parulidae	Insectivore	Arboreal	Arboreal hunter	Diurnal	Winter migrant	8.8
1	*Vermivora cyanoptera*	Passeriformes	Parulidae	Insectivore	Arboreal	Arboreal hunter	Diurnal	Winter migrant	8.4
1	*Mniotilta varia*	Passeriformes	Parulidae	Insectivore	Arboreal	Arboreal hunter	Diurnal	Winter migrant	10.8
1	*Leiothlypis peregrina*	Passeriformes	Parulidae	Insectivore	Arboreal	Arboreal hunter	Diurnal	Winter migrant	10
1	*Leiothlypis celata*	Passeriformes	Parulidae	Insectivore	Arboreal	Arboreal hunter	Diurnal	Winter migrant	9
1	*Leiothlypis crissalis*	Passeriformes	Parulidae	Insectivore	Arboreal	Arboreal hunter	Diurnal	Winter migrant	9.75
1	*Leiothlypis luciae*	Passeriformes	Parulidae	Insectivore	Arboreal	Arboreal hunter	Diurnal	Winter migrant	6.5
1	*Leiothlypis ruficapilla*	Passeriformes	Parulidae	Insectivore	Arboreal	Arboreal hunter	Diurnal	Winter migrant	8.75
1	*Leiothlypis virginiae*	Passeriformes	Parulidae	Insectivore	Arboreal	Arboreal hunter	Diurnal	Winter migrant	8.2
1	*Geothlypis poliocephala*	Passeriformes	Parulidae	Insectivore	Arboreal	Arboreal hunter	Diurnal	Winter migrant	14.6
1	*Geothlypis tolmiei*	Passeriformes	Parulidae	Insectivore	Arboreal	Arboreal hunter	Diurnal	Winter migrant	10.4
1	*Geothlypis philadelphia*	Passeriformes	Parulidae	Insectivore	Arboreal	Arboreal hunter	Diurnal	Winter migrant	12.5
1	*Geothlypis formosa*	Passeriformes	Parulidae	Insectivore	Arboreal	Arboreal hunter	Diurnal	Winter migrant	14
1	*Geothlypis speciosa*	Passeriformes	Parulidae	Insectivore	Arboreal	Arboreal hunter	Diurnal	Winter migrant	10.8
1	*Geothlypis beldingi*	Passeriformes	Parulidae	Insectivore	Arboreal	Arboreal hunter	Diurnal	Winter migrant	15.7
1	*Geothlypis flavovelata*	Passeriformes	Parulidae	Insectivore	Arboreal	Arboreal hunter	Diurnal	Winter migrant	10.9
1	*Geothlypis trichas*	Passeriformes	Parulidae	Insectivore	Arboreal	Arboreal hunter	Diurnal	Winter migrant	10.1
1	*Geothlypis nelsoni*	Passeriformes	Parulidae	Insectivore	Arboreal	Arboreal hunter	Diurnal	Winter migrant	11.1
1	*Setophaga citrina*	Passeriformes	Parulidae	Insectivore	Arboreal	Arboreal hunter	Diurnal	Winter migrant	10.625
1	*Setophaga magnolia*	Passeriformes	Parulidae	Insectivore	Arboreal	Arboreal hunter	Diurnal	Winter migrant	8.7
1	*Setophaga pensylvanica*	Passeriformes	Parulidae	Insectivore	Arboreal	Arboreal hunter	Diurnal	Winter migrant	9.6

1	*Setophaga caerulescens*	Passeriformes	Parulidae	Insectivore	Arboreal	Arboreal hunter	Diurnal	Winter migrant	10.15
1	*Setophaga pinus*	Passeriformes	Parulidae	Insectivore	Arboreal	Arboreal hunter	Diurnal	Winter migrant	11.9
1	*Setophaga coronata*	Passeriformes	Parulidae	Insectivore	Arboreal	Arboreal hunter	Diurnal	Winter migrant	12.55
1	*Setophaga dominica*	Passeriformes	Parulidae	Insectivore	Arboreal	Arboreal hunter	Diurnal	Winter migrant	9.4
1	*Setophaga discolor*	Passeriformes	Parulidae	Insectivore	Arboreal	Arboreal hunter	Diurnal	Winter migrant	7.65
1	*Setophaga nigrescens*	Passeriformes	Parulidae	Insectivore	Arboreal	Arboreal hunter	Diurnal	Winter migrant	8.35
1	*Setophaga townsendi*	Passeriformes	Parulidae	Insectivore	Arboreal	Arboreal hunter	Diurnal	Winter migrant	8.85
1	*Setophaga occidentalis*	Passeriformes	Parulidae	Insectivore	Arboreal	Arboreal hunter	Diurnal	Winter migrant	9.15
1	*Setophaga chrysoparia*	Passeriformes	Parulidae	Insectivore	Arboreal	Arboreal hunter	Diurnal	Winter migrant	10.165
1	*Setophaga virens*	Passeriformes	Parulidae	Insectivore	Arboreal	Arboreal hunter	Diurnal	Winter migrant	9.2
1	*Cardellina canadensis*	Passeriformes	Parulidae	Insectivore	Arboreal	Arboreal hunter	Diurnal	Winter migrant	10.4
1	*Cardellina pusilla*	Passeriformes	Parulidae	Insectivore	Arboreal	Arboreal hunter	Diurnal	Winter migrant	6.5
1	*Cardellina rubrifrons*	Passeriformes	Parulidae	Insectivore	Arboreal	Arboreal hunter	Diurnal	Winter migrant	9.8
1	*Icteria virens*	Passeriformes	Parulidae	Insectivore	Arboreal	Arboreal hunter	Diurnal	Winter migrant	25.3
1	*Piranga rubra*	Passeriformes	Cardinalidae	Insectivore	Arboreal	Arboreal hunter	Diurnal	Winter migrant	28.75
1	*Piranga ludoviciana*	Passeriformes	Cardinalidae	Insectivore	Arboreal	Arboreal hunter	Diurnal	Winter migrant	30
1	*Pheucticus ludovicianus*	Passeriformes	Cardinalidae	Insectivore	Arboreal	Arboreal hunter	Diurnal	Winter migrant	45.6
1	*Icterus spurius*	Passeriformes	Icteridae	Insectivore	Arboreal	Arboreal hunter	Diurnal	Winter migrant	20.05
1	*Icterus cucullatus*	Passeriformes	Icteridae	Insectivore	Arboreal	Arboreal hunter	Diurnal	Winter migrant	24.3
1	*Icterus bullockii*	Passeriformes	Icteridae	Insectivore	Arboreal	Arboreal hunter	Diurnal	Winter migrant	37.85
1	*Icterus galbula*	Passeriformes	Icteridae	Insectivore	Arboreal	Arboreal hunter	Diurnal	Winter migrant	33.75
1	*Icterus parisorum*	Passeriformes	Icteridae	Insectivore	Arboreal	Arboreal hunter	Diurnal	Winter migrant	37.4
2	*Coccyzus erythropthalmus*	Cuculiformes	Cuculidae	Insectivore	Arboreal	Arboreal hunter	Diurnal	Transient	53.45
2	*Dryobates pubescens*	Piciformes	Picidae	Insectivore	Arboreal	Arboreal hunter	Diurnal	Accidental	38.3
2	*Vireo olivaceus*	Passeriformes	Vireonidae	Insectivore	Arboreal	Arboreal hunter	Diurnal	Transient	16.7
2	*Sitta canadensis*	Passeriformes	Sittidae	Insectivore	Arboreal	Arboreal hunter	Diurnal	Transient	9.8
2	*Protonotaria citrea*	Passeriformes	Parulidae	Insectivore	Arboreal	Arboreal hunter	Diurnal	Transient	15.565
2	*Setophaga tigrina*	Passeriformes	Parulidae	Insectivore	Arboreal	Arboreal hunter	Diurnal	Transient	11
2	*Setophaga cerulea*	Passeriformes	Parulidae	Insectivore	Arboreal	Arboreal hunter	Diurnal	Transient	9.35
2	*Setophaga americana*	Passeriformes	Parulidae	Insectivore	Arboreal	Arboreal hunter	Diurnal	Transient	8.6
2	*Setophaga castanea*	Passeriformes	Parulidae	Insectivore	Arboreal	Arboreal hunter	Diurnal	Transient	12.55
2	*Setophaga fusca*	Passeriformes	Parulidae	Insectivore	Arboreal	Arboreal hunter	Diurnal	Transient	9.75
2	*Setophaga striata*	Passeriformes	Parulidae	Insectivore	Arboreal	Arboreal hunter	Diurnal	Transient	13
2	*Piranga olivacea*	Passeriformes	Cardinalidae	Insectivore	Arboreal	Arboreal hunter	Diurnal	Transient	28.6
3	*Geranospiza caerulescens*	Accipitriformes	Accipitridae	Carnivore	Arboreal	Arboreal hunter	Diurnal	Resident	344
3	*Harpia harpyja*	Accipitriformes	Accipitridae	Carnivore	Arboreal	Arboreal hunter	Diurnal	Resident	6200
3	*Spizaetus tyrannus*	Accipitriformes	Accipitridae	Carnivore	Arboreal	Arboreal hunter	Diurnal	Resident	1013
3	*Spizaetus ornatus*	Accipitriformes	Accipitridae	Carnivore	Arboreal	Arboreal hunter	Diurnal	Resident	1215
3	*Morphnus guianensis*	Accipitriformes	Accipitridae	Carnivore	Arboreal	Arboreal hunter	Diurnal	Accidental	1512
4	*Leptodon cayanensis*	Accipitriformes	Accipitridae	Insectivore	Arboreal	Arboreal hunter	Diurnal	Resident	495.75
4	*Piaya cayana*	Cuculiformes	Cuculidae	Insectivore	Arboreal	Arboreal hunter	Diurnal	Resident	108
4	*Coccyzus minor*	Cuculiformes	Cuculidae	Insectivore	Arboreal	Arboreal hunter	Diurnal	Resident	66.65
4	*Tapera naevia*	Cuculiformes	Cuculidae	Insectivore	Arboreal	Arboreal hunter	Diurnal	Resident	52.1
4	*Melanerpes formicivorus*	Piciformes	Picidae	Insectivore	Arboreal	Arboreal hunter	Diurnal	Resident	79.75
4	*Melanerpes pucherani*	Piciformes	Picidae	Insectivore	Arboreal	Arboreal hunter	Diurnal	Resident	62.5
4	*Melanerpes chrysogenys*	Piciformes	Picidae	Insectivore	Arboreal	Arboreal hunter	Diurnal	Resident	67.85
4	*Melanerpes hypopolius*	Piciformes	Picidae	Insectivore	Arboreal	Arboreal hunter	Diurnal	Resident	52.4
4	*Melanerpes pygmaeus*	Piciformes	Picidae	Insectivore	Arboreal	Arboreal hunter	Diurnal	Resident	38.6

4	*Melanerpes uropygialis*	Piciformes	Picidae	Insectivore	Arboreal	Arboreal hunter	Diurnal	Resident	67.8
4	*Melanerpes aurifrons*	Piciformes	Picidae	Insectivore	Arboreal	Arboreal hunter	Diurnal	Resident	80.9
4	*Dryobates scalaris*	Piciformes	Picidae	Insectivore	Arboreal	Arboreal hunter	Diurnal	Resident	30.3
4	*Dryobates nuttallii*	Piciformes	Picidae	Insectivore	Arboreal	Arboreal hunter	Diurnal	Resident	38.3
4	*Dryobates villosus*	Piciformes	Picidae	Insectivore	Arboreal	Arboreal hunter	Diurnal	Resident	19
4	*Dryobates fumigatus*	Piciformes	Picidae	Insectivore	Arboreal	Arboreal hunter	Diurnal	Resident	34.7
4	*Dryobates arizonae*	Piciformes	Picidae	Insectivore	Arboreal	Arboreal hunter	Diurnal	Resident	46.75
4	*Dryobates stricklandi*	Piciformes	Picidae	Insectivore	Arboreal	Arboreal hunter	Diurnal	Resident	46.75
4	*Colaptes rubiginosus*	Piciformes	Picidae	Insectivore	Arboreal	Arboreal hunter	Diurnal	Resident	55.8
4	*Colaptes auricularis*	Piciformes	Picidae	Insectivore	Arboreal	Arboreal hunter	Diurnal	Resident	65.4
4	*Celeus castaneus*	Piciformes	Picidae	Insectivore	Arboreal	Arboreal hunter	Diurnal	Resident	86.6
4	*Dryocopus lineatus*	Piciformes	Picidae	Insectivore	Arboreal	Arboreal hunter	Diurnal	Resident	183.5
4	*Campephilus guatemalensis*	Piciformes	Picidae	Insectivore	Arboreal	Arboreal hunter	Diurnal	Resident	248.5
4	*Campephilus imperialis*	Piciformes	Picidae	Insectivore	Arboreal	Arboreal hunter	Diurnal	Resident	510
4	*Ibycter americanus*	Falconiformes	Falconidae	Insectivore	Arboreal	Arboreal hunter	Diurnal	Resident	586
4	*Thamnophilus doliatus*	Passeriformes	Thamnophilidae	Insectivore	Arboreal	Arboreal hunter	Diurnal	Resident	27.9
4	*Thamnistes anabatinus*	Passeriformes	Thamnophilidae	Insectivore	Arboreal	Arboreal hunter	Diurnal	Resident	20.7
4	*Dysithamnus mentalis*	Passeriformes	Thamnophilidae	Insectivore	Arboreal	Arboreal hunter	Diurnal	Resident	12.8
4	*Myrmotherula schisticolor*	Passeriformes	Thamnophilidae	Insectivore	Arboreal	Arboreal hunter	Diurnal	Resident	9.6
4	*Microrhopias quixensis*	Passeriformes	Thamnophilidae	Insectivore	Arboreal	Arboreal hunter	Diurnal	Resident	7.9
4	*Sittasomus griseicapillus*	Passeriformes	Furnariidae	Insectivore	Arboreal	Arboreal hunter	Diurnal	Resident	14.6
4	*Glyphorynchus spirurus*	Passeriformes	Furnariidae	Insectivore	Arboreal	Arboreal hunter	Diurnal	Resident	14.6
4	*Dendrocolaptes sanctithomae*	Passeriformes	Furnariidae	Insectivore	Arboreal	Arboreal hunter	Diurnal	Resident	66.9
4	*Dendrocolaptes picumnus*	Passeriformes	Furnariidae	Insectivore	Arboreal	Arboreal hunter	Diurnal	Resident	87.6
4	*Xiphocolaptes promeropirhynchus*	Passeriformes	Furnariidae	Insectivore	Arboreal	Arboreal hunter	Diurnal	Resident	117
4	*Xiphorhynchus flavigaster*	Passeriformes	Furnariidae	Insectivore	Arboreal	Arboreal hunter	Diurnal	Resident	43.6
4	*Xiphorhynchus erythropygius*	Passeriformes	Furnariidae	Insectivore	Arboreal	Arboreal hunter	Diurnal	Resident	46.8
4	*Lepidocolaptes leucogaster*	Passeriformes	Furnariidae	Insectivore	Arboreal	Arboreal hunter	Diurnal	Resident	36
4	*Lepidocolaptes souleyetii*	Passeriformes	Furnariidae	Insectivore	Arboreal	Arboreal hunter	Diurnal	Resident	25.7
4	*Lepidocolaptes affinis*	Passeriformes	Furnariidae	Insectivore	Arboreal	Arboreal hunter	Diurnal	Resident	35
4	*Xenops minutus*	Passeriformes	Furnariidae	Insectivore	Arboreal	Arboreal hunter	Diurnal	Resident	10.6
4	*Anabacerthia variegaticeps*	Passeriformes	Furnariidae	Insectivore	Arboreal	Arboreal hunter	Diurnal	Resident	24.8
4	*Ornithion semiflavum*	Passeriformes	Tyrannidae	Insectivore	Arboreal	Arboreal hunter	Diurnal	Resident	7
4	*Myiopagis viridicata*	Passeriformes	Tyrannidae	Insectivore	Arboreal	Arboreal hunter	Diurnal	Resident	12.3
4	*Oncostoma cinereigulare*	Passeriformes	Tyrannidae	Insectivore	Arboreal	Arboreal hunter	Diurnal	Resident	6.1
4	*Poecilotriccus sylvia*	Passeriformes	Tyrannidae	Insectivore	Arboreal	Arboreal hunter	Diurnal	Resident	7.1
4	*Rhynchocyclus brevirostris*	Passeriformes	Tyrannidae	Insectivore	Arboreal	Arboreal hunter	Diurnal	Resident	22.8
4	*Platyrinchus cancrominus*	Passeriformes	Tyrannidae	Insectivore	Arboreal	Arboreal hunter	Diurnal	Resident	12
4	*Rhytipterna holerythra*	Passeriformes	Tyrannidae	Insectivore	Arboreal	Arboreal hunter	Diurnal	Resident	36.8
4	*Pitangus sulphuratus*	Passeriformes	Tyrannidae	Insectivore	Arboreal	Arboreal hunter	Diurnal	Resident	61
4	*Laniocera rufescens*	Passeriformes	Tityridae	Insectivore	Arboreal	Arboreal hunter	Diurnal	Resident	48.1
4	*Tityra semifasciata*	Passeriformes	Tityridae	Insectivore	Arboreal	Arboreal hunter	Diurnal	Resident	79.3
4	*Tityra inquisitor*	Passeriformes	Tityridae	Insectivore	Arboreal	Arboreal hunter	Diurnal	Resident	43.3
4	*Lipaugus unirufus*	Passeriformes	Cotingidae	Insectivore	Arboreal	Arboreal hunter	Diurnal	Resident	82.1
4	*Vireo brevipennis*	Passeriformes	Vireonidae	Insectivore	Arboreal	Arboreal hunter	Diurnal	Resident	11.8
4	*Vireo pallens*	Passeriformes	Vireonidae	Insectivore	Arboreal	Arboreal hunter	Diurnal	Resident	11.6
4	*Vireo bairdi*	Passeriformes	Vireonidae	Insectivore	Arboreal	Arboreal hunter	Diurnal	Resident	12
4	*Vireo nelsoni*	Passeriformes	Vireonidae	Insectivore	Arboreal	Arboreal hunter	Diurnal	Resident	9.4

4	*Vireo huttoni*	Passeriformes	Vireonidae	Insectivore	Arboreal	Arboreal hunter	Diurnal	Resident	11.6
4	*Vireo hypochryseus*	Passeriformes	Vireonidae	Insectivore	Arboreal	Arboreal hunter	Diurnal	Resident	12
4	*Vireo leucophrys*	Passeriformes	Vireonidae	Insectivore	Arboreal	Arboreal hunter	Diurnal	Resident	12.5
4	*Vireo magister*	Passeriformes	Vireonidae	Insectivore	Arboreal	Arboreal hunter	Diurnal	Resident	15.4
4	*Tunchiornis ochraceiceps*	Passeriformes	Vireonidae	Insectivore	Arboreal	Arboreal hunter	Diurnal	Resident	10.4
4	*Pachysylvia decurtata*	Passeriformes	Vireonidae	Insectivore	Arboreal	Arboreal hunter	Diurnal	Resident	8.8
4	*Vireolanius melitophrys*	Passeriformes	Vireonidae	Insectivore	Arboreal	Arboreal hunter	Diurnal	Resident	34.7
4	*Vireolanius pulchellus*	Passeriformes	Vireonidae	Insectivore	Arboreal	Arboreal hunter	Diurnal	Resident	24
4	*Cyclarhis gujanensis*	Passeriformes	Vireonidae	Insectivore	Arboreal	Arboreal hunter	Diurnal	Resident	28.8
4	*Cyanolyca nanus*	Passeriformes	Corvidae	Insectivore	Arboreal	Arboreal hunter	Diurnal	Resident	41
4	*Poecile gambeli*	Passeriformes	Paridae	Insectivore	Arboreal	Arboreal hunter	Diurnal	Resident	10.8
4	*Poecile sclateri*	Passeriformes	Paridae	Insectivore	Arboreal	Arboreal hunter	Diurnal	Resident	11
4	*Baeolophus wollweberi*	Passeriformes	Paridae	Insectivore	Arboreal	Arboreal hunter	Diurnal	Resident	10.8
4	*Baeolophus inornatus*	Passeriformes	Paridae	Insectivore	Arboreal	Arboreal hunter	Diurnal	Resident	15.95
4	*Baeolophus atricristatus*	Passeriformes	Paridae	Insectivore	Arboreal	Arboreal hunter	Diurnal	Resident	16.55
4	*Baeolophus ridgwayi*	Passeriformes	Paridae	Insectivore	Arboreal	Arboreal hunter	Diurnal	Resident	16
4	*Auriparus flaviceps*	Passeriformes	Remizidae	Insectivore	Arboreal	Arboreal hunter	Diurnal	Resident	6.85
4	*Psaltriparus minimus*	Passeriformes	Aegithalidae	Insectivore	Arboreal	Arboreal hunter	Diurnal	Resident	5.3
4	*Sitta carolinensis*	Passeriformes	Sittidae	Insectivore	Arboreal	Arboreal hunter	Diurnal	Resident	21.1
4	*Sitta pygmaea*	Passeriformes	Sittidae	Insectivore	Arboreal	Arboreal hunter	Diurnal	Resident	10.6
4	*Salpinctes obsoletus*	Passeriformes	Troglodytidae	Insectivore	Arboreal	Arboreal hunter	Diurnal	Resident	17.25
4	*Pheugopedius maculipectus*	Passeriformes	Troglodytidae	Insectivore	Arboreal	Arboreal hunter	Diurnal	Resident	14.85
4	*Pheugopedius felix*	Passeriformes	Troglodytidae	Insectivore	Arboreal	Arboreal hunter	Diurnal	Resident	13.2
4	*Ramphocaenus melanurus*	Passeriformes	Polioptilidae	Insectivore	Arboreal	Arboreal hunter	Diurnal	Resident	9.7
4	*Polioptila californica*	Passeriformes	Polioptilidae	Insectivore	Arboreal	Arboreal hunter	Diurnal	Resident	6
4	*Polioptila melanura*	Passeriformes	Polioptilidae	Insectivore	Arboreal	Arboreal hunter	Diurnal	Resident	5.3
4	*Polioptila nigriceps*	Passeriformes	Polioptilidae	Insectivore	Arboreal	Arboreal hunter	Diurnal	Resident	5.6
4	*Polioptila albiloris*	Passeriformes	Polioptilidae	Insectivore	Arboreal	Arboreal hunter	Diurnal	Resident	5.7
4	*Polioptila bilineata*	Passeriformes	Polioptilidae	Insectivore	Arboreal	Arboreal hunter	Diurnal	Resident	6
4	*Polioptila albiventris*	Passeriformes	Polioptilidae	Insectivore	Arboreal	Arboreal hunter	Diurnal	Resident	5.6
4	*Regulus satrapa*	Passeriformes	Regulidae	Insectivore	Arboreal	Arboreal hunter	Diurnal	Resident	6.2
4	*Chamaea fasciata*	Passeriformes	Sylviidae	Insectivore	Arboreal	Arboreal hunter	Diurnal	Resident	14.715
4	*Turdus infuscatus*	Passeriformes	Turdidae	Insectivore	Arboreal	Arboreal hunter	Diurnal	Resident	73.8
4	*Turdus plebejus*	Passeriformes	Turdidae	Insectivore	Arboreal	Arboreal hunter	Diurnal	Resident	86.5
4	*Ridgwayia pinicola*	Passeriformes	Turdidae	Insectivore	Arboreal	Arboreal hunter	Diurnal	Resident	88
4	*Peucedramus taeniatus*	Passeriformes	Peucedramidae	Insectivore	Arboreal	Arboreal hunter	Diurnal	Resident	11.15
4	*Oreothlypis superciliosa*	Passeriformes	Parulidae	Insectivore	Arboreal	Arboreal hunter	Diurnal	Resident	9
4	*Setophaga pitiayumi*	Passeriformes	Parulidae	Insectivore	Arboreal	Arboreal hunter	Diurnal	Resident	6.9
4	*Setophaga petechia*	Passeriformes	Parulidae	Insectivore	Arboreal	Arboreal hunter	Diurnal	Resident	9.5
4	*Setophaga graciae*	Passeriformes	Parulidae	Insectivore	Arboreal	Arboreal hunter	Diurnal	Resident	10.2
4	*Basileuterus lachrymosus*	Passeriformes	Parulidae	Insectivore	Arboreal	Arboreal hunter	Diurnal	Resident	15.2
4	*Basileuterus rufifrons*	Passeriformes	Parulidae	Insectivore	Arboreal	Arboreal hunter	Diurnal	Resident	10.9
4	*Basileuterus belli*	Passeriformes	Parulidae	Insectivore	Arboreal	Arboreal hunter	Diurnal	Resident	10.4
4	*Basileuterus culicivorus*	Passeriformes	Parulidae	Insectivore	Arboreal	Arboreal hunter	Diurnal	Resident	10.5
4	*Cardellina rubra*	Passeriformes	Parulidae	Insectivore	Arboreal	Arboreal hunter	Diurnal	Resident	8.1
4	*Cardellina versicolor*	Passeriformes	Parulidae	Insectivore	Arboreal	Arboreal hunter	Diurnal	Resident	10
4	*Myioborus pictus*	Passeriformes	Parulidae	Insectivore	Arboreal	Arboreal hunter	Diurnal	Resident	9.8
4	*Myioborus miniatus*	Passeriformes	Parulidae	Insectivore	Arboreal	Arboreal hunter	Diurnal	Resident	9.5

4	*Rhodinocichla rosea*	Passeriformes	Thraupidae	Insectivore	Arboreal	Arboreal hunter	Diurnal	Resident	48
4	*Lanio aurantius*	Passeriformes	Thraupidae	Insectivore	Arboreal	Arboreal hunter	Diurnal	Resident	35
4	*Ramphocelus sanguinolentus*	Passeriformes	Thraupidae	Insectivore	Arboreal	Arboreal hunter	Diurnal	Resident	41
4	*Chlorospingus flavopectus*	Passeriformes	Emberizidae	Insectivore	Arboreal	Arboreal hunter	Diurnal	Resident	22
4	*Piranga roseogularis*	Passeriformes	Cardinalidae	Insectivore	Arboreal	Arboreal hunter	Diurnal	Resident	24
4	*Piranga flava*	Passeriformes	Cardinalidae	Insectivore	Arboreal	Arboreal hunter	Diurnal	Resident	38
4	*Piranga bidentata*	Passeriformes	Cardinalidae	Insectivore	Arboreal	Arboreal hunter	Diurnal	Resident	34.7
4	*Piranga leucoptera*	Passeriformes	Cardinalidae	Insectivore	Arboreal	Arboreal hunter	Diurnal	Resident	16
4	*Piranga erythrocephala*	Passeriformes	Cardinalidae	Insectivore	Arboreal	Arboreal hunter	Diurnal	Resident	21.8
4	*Habia rubica*	Passeriformes	Cardinalidae	Insectivore	Arboreal	Arboreal hunter	Diurnal	Resident	32.5
4	*Habia fuscicauda*	Passeriformes	Cardinalidae	Insectivore	Arboreal	Arboreal hunter	Diurnal	Resident	38.5
4	*Caryothraustes poliogaster*	Passeriformes	Cardinalidae	Insectivore	Arboreal	Arboreal hunter	Diurnal	Resident	41.8
4	*Granatellus venustus*	Passeriformes	Cardinalidae	Insectivore	Arboreal	Arboreal hunter	Diurnal	Resident	10.8
4	*Granatellus sallaei*	Passeriformes	Cardinalidae	Insectivore	Arboreal	Arboreal hunter	Diurnal	Resident	9.9
4	*Icterus wagleri*	Passeriformes	Icteridae	Insectivore	Arboreal	Arboreal hunter	Diurnal	Resident	42.4
4	*Icterus maculialatus*	Passeriformes	Icteridae	Insectivore	Arboreal	Arboreal hunter	Diurnal	Resident	37.4
4	*Icterus prosthemelas*	Passeriformes	Icteridae	Insectivore	Arboreal	Arboreal hunter	Diurnal	Resident	28
4	*Icterus chrysater*	Passeriformes	Icteridae	Insectivore	Arboreal	Arboreal hunter	Diurnal	Resident	53.6
4	*Icterus mesomelas*	Passeriformes	Icteridae	Insectivore	Arboreal	Arboreal hunter	Diurnal	Resident	39.9
4	*Icterus pustulatus*	Passeriformes	Icteridae	Insectivore	Arboreal	Arboreal hunter	Diurnal	Resident	37
4	*Icterus auratus*	Passeriformes	Icteridae	Insectivore	Arboreal	Arboreal hunter	Diurnal	Resident	32.1
4	*Icterus pectoralis*	Passeriformes	Icteridae	Insectivore	Arboreal	Arboreal hunter	Diurnal	Resident	44.5
4	*Icterus gularis*	Passeriformes	Icteridae	Insectivore	Arboreal	Arboreal hunter	Diurnal	Resident	55.3
4	*Icterus graduacauda*	Passeriformes	Icteridae	Insectivore	Arboreal	Arboreal hunter	Diurnal	Resident	41.95
4	*Icterus abeillei*	Passeriformes	Icteridae	Insectivore	Arboreal	Arboreal hunter	Diurnal	Resident	33
4	*Amblycercus holosericeus*	Passeriformes	Icteridae	Insectivore	Arboreal	Arboreal hunter	Diurnal	Resident	63.7
4	*Cassiculus melanicterus*	Passeriformes	Icteridae	Insectivore	Arboreal	Arboreal hunter	Diurnal	Resident	79.8
5	*Harpagus bidentatus*	Accipitriformes	Accipitridae	Insectivore	Volant	Arboreal hunter	Diurnal	Resident	210.5
5	*Glaucidium brasilianum*	Strigiformes	Strigidae	Insectivore	Volant	Arboreal hunter	Diurnal	Resident	69.65
5	*Electron carinatum*	Coraciiformes	Momotidae	Insectivore	Volant	Arboreal hunter	Diurnal	Resident	64.9
5	*Notharchus hyperrhynchus*	Piciformes	Bucconidae	Insectivore	Volant	Arboreal hunter	Diurnal	Resident	95.9
5	*Malacoptila panamensis*	Piciformes	Bucconidae	Insectivore	Volant	Arboreal hunter	Diurnal	Resident	42.6
5	*Taraba major*	Passeriformes	Thamnophilidae	Insectivore	Volant	Arboreal hunter	Diurnal	Resident	67.5
5	*Zimmerius vilissimus*	Passeriformes	Tyrannidae	Insectivore	Volant	Arboreal hunter	Diurnal	Resident	10
6	*Clibanornis rubiginosus*	Passeriformes	Furnariidae	Insectivore	Semi-arboreal	Arboreal hunter	Diurnal	Resident	31
6	*Synallaxis erythrothorax*	Passeriformes	Furnariidae	Insectivore	Semi-arboreal	Arboreal hunter	Diurnal	Resident	17.1
6	*Xenotriccus callizonus*	Passeriformes	Tyrannidae	Insectivore	Semi-arboreal	Arboreal hunter	Diurnal	Resident	11.2
6	*Xenotriccus mexicanus*	Passeriformes	Tyrannidae	Insectivore	Semi-arboreal	Arboreal hunter	Diurnal	Resident	13.8
6	*Troglodytes aedon*	Passeriformes	Troglodytidae	Insectivore	Semi-arboreal	Arboreal hunter	Diurnal	Resident	10.9
6	*Troglodytes rufociliatus*	Passeriformes	Troglodytidae	Insectivore	Semi-arboreal	Arboreal hunter	Diurnal	Resident	11
6	*Troglodytes hiemalis*	Passeriformes	Troglodytidae	Insectivore	Semi-arboreal	Arboreal hunter	Diurnal	Winter migrant	9
6	*Troglodytes sissonii*	Passeriformes	Troglodytidae	Insectivore	Semi-arboreal	Arboreal hunter	Diurnal	Resident	10
6	*Troglodytes tanneri*	Passeriformes	Troglodytidae	Insectivore	Semi-arboreal	Arboreal hunter	Diurnal	Resident	10
6	*Troglodytes pacificus*	Passeriformes	Troglodytidae	Insectivore	Semi-arboreal	Arboreal hunter	Diurnal	Winter migrant	10
6	*Thryothorus ludovicianus*	Passeriformes	Troglodytidae	Insectivore	Semi-arboreal	Arboreal hunter	Diurnal	Resident	20.05
6	*Campylorhynchus zonatus*	Passeriformes	Troglodytidae	Insectivore	Semi-arboreal	Arboreal hunter	Diurnal	Resident	34.6
6	*Campylorhynchus rufinucha*	Passeriformes	Troglodytidae	Insectivore	Semi-arboreal	Arboreal hunter	Diurnal	Resident	30.4
6	*Thryophilus sinaloa*	Passeriformes	Troglodytidae	Insectivore	Semi-arboreal	Arboreal hunter	Diurnal	Resident	14.85

6	*Thryophilus pleurostictus*	Passeriformes	Troglodytidae	Insectivore	Semi-arboreal	Arboreal hunter	Diurnal	Resident	18.1
6	*Eucometis penicillata*	Passeriformes	Thraupidae	Insectivore	Semi-arboreal	Arboreal hunter	Diurnal	Resident	27
7	*Accipiter striatus*	Accipitriformes	Accipitridae	Carnivore	Volant	Air hunter under canopy	Diurnal	Winter migrant	138.5
7	*Accipiter cooperii*	Accipitriformes	Accipitridae	Carnivore	Volant	Air hunter under canopy	Diurnal	Winter migrant	439
7	*Accipiter gentilis*	Accipitriformes	Accipitridae	Carnivore	Volant	Air hunter under canopy	Diurnal	Winter migrant	845
7	*Falco columbarius*	Falconiformes	Falconidae	Carnivore	Volant	Air hunter under canopy	Diurnal	Winter migrant	167
7	*Falco peregrinus*	Falconiformes	Falconidae	Carnivore	Volant	Air hunter under canopy	Diurnal	Winter migrant	781.5
8	*Elanoides forficatus*	Accipitriformes	Accipitridae	Carnivore	Volant	Air hunter under canopy	Diurnal	Resident	453.5
8	*Accipiter bicolor*	Accipitriformes	Accipitridae	Carnivore	Volant	Air hunter under canopy	Diurnal	Resident	340.5
8	*Spizaetus melanoleucus*	Accipitriformes	Accipitridae	Carnivore	Volant	Arboreal hunter	Diurnal	Resident	850
8	*Falco femoralis*	Falconiformes	Falconidae	Carnivore	Volant	Air hunter under canopy	Diurnal	Resident	333.5
8	*Falco rufigularis*	Falconiformes	Falconidae	Carnivore	Volant	Air hunter under canopy	Diurnal	Resident	165.5
8	*Falco deiroleucus*	Falconiformes	Falconidae	Carnivore	Volant	Air hunter under canopy	Diurnal	Resident	654
9	*Buteo lineatus*	Accipitriformes	Accipitridae	Carnivore	Volant	Ground hunter	Diurnal	Winter migrant	606.9
9	*Buteo platypterus*	Accipitriformes	Accipitridae	Carnivore	Volant	Ground hunter	Diurnal	Winter migrant	455
9	*Buteo swainsoni*	Accipitriformes	Accipitridae	Carnivore	Volant	Ground hunter	Diurnal	Winter migrant	988.5
9	*Buteo albonotatus*	Accipitriformes	Accipitridae	Carnivore	Volant	Ground hunter	Diurnal	Winter migrant	757
9	*Buteo jamaicensis*	Accipitriformes	Accipitridae	Carnivore	Volant	Ground hunter	Diurnal	Winter migrant	1126
9	*Buteo regalis*	Accipitriformes	Accipitridae	Carnivore	Volant	Ground hunter	Diurnal	Winter migrant	1469.5
9	*Buteo lagopus*	Accipitriformes	Accipitridae	Carnivore	Volant	Ground hunter	Diurnal	Winter migrant	1152.5
9	*Aquila chrysaetos*	Accipitriformes	Accipitridae	Carnivore	Volant	Ground hunter	Diurnal	Winter migrant	4195
9	*Circus hudsonius*	Accipitriformes	Accipitridae	Carnivore	Volant	Ground hunter	Diurnal	Winter migrant	550
9	*Asio otus*	Strigiformes	Strigidae	Carnivore	Volant	Ground hunter	Nocturnal	Winter migrant	262
9	*Asio flammeus*	Strigiformes	Strigidae	Carnivore	Volant	Ground hunter	Cathemeral	Winter migrant	346.5
10	*Tyto alba*	Strigiformes	Tytonidae	Carnivore	Volant	Ground hunter	Nocturnal	Resident	519.95
10	*Pulsatrix perspicillata*	Strigiformes	Strigidae	Carnivore	Volant	Ground hunter	Nocturnal	Resident	873
10	*Bubo virginianus*	Strigiformes	Strigidae	Carnivore	Volant	Ground hunter	Nocturnal	Resident	1505
10	*Strix virgata*	Strigiformes	Strigidae	Carnivore	Volant	Ground hunter	Nocturnal	Resident	240
10	*Strix nigrolineata*	Strigiformes	Strigidae	Carnivore	Volant	Ground hunter	Nocturnal	Resident	350
10	*Strix occidentalis*	Strigiformes	Strigidae	Carnivore	Volant	Ground hunter	Nocturnal	Resident	609.5
10	*Strix varia*	Strigiformes	Strigidae	Carnivore	Volant	Ground hunter	Nocturnal	Resident	716.5
10	*Strix fulvescens*	Strigiformes	Strigidae	Carnivore	Volant	Ground hunter	Nocturnal	Resident	716.5
10	*Asio stygius*	Strigiformes	Strigidae	Carnivore	Volant	Ground hunter	Nocturnal	Resident	653.5
10	*Asio clamator*	Strigiformes	Strigidae	Carnivore	Volant	Ground hunter	Nocturnal	Resident	446
10	*Aegolius acadicus*	Strigiformes	Strigidae	Carnivore	Volant	Ground hunter	Nocturnal	Resident	82.85
10	*Aegolius ridgwayi*	Strigiformes	Strigidae	Carnivore	Volant	Ground hunter	Nocturnal	Resident	80
11	*Chondrohierax uncinatus*	Accipitriformes	Accipitridae	Carnivore	Semi-arboreal	Ground hunter	Diurnal	Resident	276.5
11	*Elanus leucurus*	Accipitriformes	Accipitridae	Carnivore	Volant	Ground hunter	Diurnal	Resident	333
11	*Buteogallus urubitinga*	Accipitriformes	Accipitridae	Carnivore	Volant	Ground hunter	Diurnal	Resident	996.5
11	*Buteogallus solitarius*	Accipitriformes	Accipitridae	Carnivore	Volant	Ground hunter	Diurnal	Resident	3000
11	*Parabuteo unicinctus*	Accipitriformes	Accipitridae	Carnivore	Volant	Ground hunter	Diurnal	Resident	198.5
11	*Pseudastur albicollis*	Accipitriformes	Accipitridae	Carnivore	Volant	Ground hunter	Diurnal	Resident	625
11	*Buteo plagiatus*	Accipitriformes	Accipitridae	Carnivore	Volant	Ground hunter	Diurnal	Resident	528.2
11	*Buteo brachyurus*	Accipitriformes	Accipitridae	Carnivore	Volant	Ground hunter	Diurnal	Resident	502.5
11	*Geranoaetus albicaudatus*	Accipitriformes	Accipitridae	Carnivore	Volant	Ground hunter	Diurnal	Resident	983
11	*Glaucidium gnoma*	Strigiformes	Strigidae	Carnivore	Volant	Ground hunter	Diurnal	Resident	67.45
11	*Glaucidium sanchezi*	Strigiformes	Strigidae	Carnivore	Volant	Ground hunter	Diurnal	Resident	55
11	*Glaucidium palmarum*	Strigiformes	Strigidae	Carnivore	Volant	Ground hunter	Diurnal	Resident	45.7

11	*Micrastur ruficollis*	Falconiformes	Falconidae	Carnivore	Volant	Ground hunter	Diurnal	Resident	178.5
11	*Micrastur semitorquatus*	Falconiformes	Falconidae	Carnivore	Volant	Ground hunter	Diurnal	Resident	731
11	*Herpetotheres cachinnans*	Falconiformes	Falconidae	Carnivore	Volant	Ground hunter	Diurnal	Resident	667.5
11	*Falco mexicanus*	Falconiformes	Falconidae	Carnivore	Volant	Ground hunter	Diurnal	Resident	708.5
12	*Bartramia longicauda*	Charadriiformes	Scolopacidae	Insectivore	Semi-aquatic	Ground hunter	Diurnal	Transient	139.5
13	*Colaptes chrysoides*	Piciformes	Picidae	Insectivore	Semi-arboreal	Ground hunter	Diurnal	Resident	135.71
13	*Cercomacroides tyrannina*	Passeriformes	Thamnophilidae	Insectivore	Semi-arboreal	Ground hunter	Diurnal	Resident	16.3
13	*Dendrocincla anabatina*	Passeriformes	Furnariidae	Insectivore	Semi-arboreal	Ground hunter	Diurnal	Resident	34.35
13	*Dendrocincla homochroa*	Passeriformes	Furnariidae	Insectivore	Semi-arboreal	Ground hunter	Diurnal	Resident	41.05
13	*Automolus ochrolaemus*	Passeriformes	Furnariidae	Insectivore	Semi-arboreal	Ground hunter	Diurnal	Resident	40.2
13	*Thryomanes bewickii*	Passeriformes	Troglodytidae	Insectivore	Semi-arboreal	Ground hunter	Diurnal	Resident	9.9
13	*Campylorhynchus megalopterus*	Passeriformes	Troglodytidae	Insectivore	Semi-arboreal	Ground hunter	Diurnal	Resident	33.1
13	*Campylorhynchus chiapensis*	Passeriformes	Troglodytidae	Insectivore	Semi-arboreal	Ground hunter	Diurnal	Resident	50.9
13	*Campylorhynchus gularis*	Passeriformes	Troglodytidae	Insectivore	Semi-arboreal	Ground hunter	Diurnal	Resident	30.1
13	*Campylorhynchus jocosus*	Passeriformes	Troglodytidae	Insectivore	Semi-arboreal	Ground hunter	Diurnal	Resident	27.6
13	*Campylorhynchus yucatanicus*	Passeriformes	Troglodytidae	Insectivore	Semi-arboreal	Ground hunter	Diurnal	Resident	35.5
13	*Campylorhynchus brunneicapillus*	Passeriformes	Troglodytidae	Insectivore	Semi-arboreal	Ground hunter	Diurnal	Resident	37.2
13	*Thryophilus rufalbus*	Passeriformes	Troglodytidae	Insectivore	Semi-arboreal	Ground hunter	Diurnal	Resident	26.5
13	*Cantorchilus modestus*	Passeriformes	Troglodytidae	Insectivore	Semi-arboreal	Ground hunter	Diurnal	Resident	18.8
13	*Uropsila leucogastra*	Passeriformes	Troglodytidae	Insectivore	Semi-arboreal	Ground hunter	Diurnal	Resident	9.05
13	*Henicorhina leucosticta*	Passeriformes	Troglodytidae	Insectivore	Semi-arboreal	Ground hunter	Diurnal	Resident	15.7
13	*Henicorhina leucophrys*	Passeriformes	Troglodytidae	Insectivore	Semi-arboreal	Ground hunter	Diurnal	Resident	17.25
14	*Colaptes auratus*	Piciformes	Picidae	Insectivore	Semi-arboreal	Ground hunter	Diurnal	Winter migrant	147.85
14	*Cistothorus platensis*	Passeriformes	Troglodytidae	Insectivore	Semi-arboreal	Ground hunter	Diurnal	Winter migrant	7.99
14	*Cistothorus palustris*	Passeriformes	Troglodytidae	Insectivore	Semi-arboreal	Ground hunter	Diurnal	Winter migrant	10
14	*Limnothlypis swainsonii*	Passeriformes	Parulidae	Insectivore	Semi-arboreal	Ground hunter	Diurnal	Winter migrant	18.9
14	*Oporornis agilis*	Passeriformes	Parulidae	Insectivore	Semi-arboreal	Ground hunter	Diurnal	Winter migrant	15.2
14	*Setophaga palmarum*	Passeriformes	Parulidae	Insectivore	Semi-arboreal	Ground hunter	Diurnal	Winter migrant	10.3
15	*Vanellus chilensis*	Charadriiformes	Charadriidae	Insectivore	Terrestrial	Ground hunter	Diurnal	Accidental	327
15	*Catharus fuscescens*	Passeriformes	Turdidae	Insectivore	Terrestrial	Ground hunter	Diurnal	Winter migrant	33.65
15	*Catharus minimus*	Passeriformes	Turdidae	Insectivore	Terrestrial	Ground hunter	Diurnal	Winter migrant	31.6
15	*Catharus guttatus*	Passeriformes	Turdidae	Insectivore	Terrestrial	Ground hunter	Diurnal	Winter migrant	29.65
15	*Hylocichla mustelina*	Passeriformes	Turdidae	Insectivore	Terrestrial	Ground hunter	Diurnal	Winter migrant	57.25
15	*Ixoreus naevius*	Passeriformes	Turdidae	Insectivore	Terrestrial	Ground hunter	Diurnal	Winter migrant	78.4
15	*Oreoscoptes montanus*	Passeriformes	Mimidae	Insectivore	Terrestrial	Ground hunter	Diurnal	Winter migrant	44.25
15	*Toxostoma rufum*	Passeriformes	Mimidae	Insectivore	Terrestrial	Ground hunter	Diurnal	Transient	68.8
15	*Anthus rubescens*	Passeriformes	Motacillidae	Insectivore	Terrestrial	Ground hunter	Diurnal	Winter migrant	20.85
15	*Anthus spragueii*	Passeriformes	Motacillidae	Insectivore	Terrestrial	Ground hunter	Diurnal	Winter migrant	23.3
15	*Anthus hodgsoni*	Passeriformes	Motacillidae	Insectivore	Terrestrial	Ground hunter	Diurnal	Accidental	21
15	*Parkesia motacilla*	Passeriformes	Parulidae	Insectivore	Terrestrial	Ground hunter	Diurnal	Winter migrant	20.3
15	*Parkesia noveboracensis*	Passeriformes	Parulidae	Insectivore	Terrestrial	Ground hunter	Diurnal	Winter migrant	17.9
15	*Peucaea cassinii*	Passeriformes	Emberizidae	Insectivore	Terrestrial	Ground hunter	Diurnal	Winter migrant	18.9
15	*Calamospiza melanocorys*	Passeriformes	Emberizidae	Insectivore	Terrestrial	Ground hunter	Diurnal	Winter migrant	38.025
15	*Ammodramus savannarum*	Passeriformes	Emberizidae	Insectivore	Terrestrial	Ground hunter	Diurnal	Winter migrant	17
15	*Ammospiza maritima*	Passeriformes	Emberizidae	Insectivore	Terrestrial	Ground hunter	Diurnal	Winter migrant	23.25
15	*Melospiza georgiana*	Passeriformes	Emberizidae	Insectivore	Terrestrial	Ground hunter	Diurnal	Winter migrant	17
15	*Passerina caerulea*	Passeriformes	Cardinalidae	Insectivore	Terrestrial	Ground hunter	Diurnal	Winter migrant	14.5
15	*Passerina amoena*	Passeriformes	Cardinalidae	Insectivore	Terrestrial	Ground hunter	Diurnal	Winter migrant	15.3

15	*Sturnella neglecta*	Passeriformes	Icteridae	Insectivore	Terrestrial	Ground hunter	Diurnal	Winter migrant	104.15
15	*Euphagus carolinus*	Passeriformes	Icteridae	Insectivore	Terrestrial	Ground hunter	Diurnal	Winter migrant	59.75
15	*Euphagus cyanocephalus*	Passeriformes	Icteridae	Insectivore	Terrestrial	Ground hunter	Diurnal	Winter migrant	69.3
16	*Burhinus bistriatus*	Charadriiformes	Burhinidae	Insectivore	Terrestrial	Ground hunter	Cathemeral	Resident	780
16	*Dromococcyx phasianellus*	Cuculiformes	Cuculidae	Insectivore	Terrestrial	Ground hunter	Diurnal	Resident	84.5
16	*Morococcyx erythropygus*	Cuculiformes	Cuculidae	Insectivore	Terrestrial	Ground hunter	Diurnal	Resident	65.1
16	*Crotophaga ani*	Cuculiformes	Cuculidae	Insectivore	Terrestrial	Ground hunter	Diurnal	Resident	97.35
16	*Crotophaga sulcirostris*	Cuculiformes	Cuculidae	Insectivore	Terrestrial	Ground hunter	Diurnal	Resident	82.2
16	*Nyctidromus albicollis*	Caprimulgiformes	Caprimulgidae	Insectivore	Terrestrial	Ground hunter	Nocturnal	Resident	53.05
16	*Phalaenoptilus nuttallii*	Caprimulgiformes	Caprimulgidae	Insectivore	Terrestrial	Ground hunter	Nocturnal	Resident	48.35
16	*Nyctiphrynus mcleodii*	Caprimulgiformes	Caprimulgidae	Insectivore	Terrestrial	Ground hunter	Nocturnal	Resident	35
16	*Nyctiphrynus yucatanicus*	Caprimulgiformes	Caprimulgidae	Insectivore	Terrestrial	Ground hunter	Nocturnal	Resident	24.5
16	*Grallaria guatimalensis*	Passeriformes	Grallariidae	Insectivore	Terrestrial	Ground hunter	Diurnal	Resident	94.1
16	*Formicarius analis*	Passeriformes	Formicariidae	Insectivore	Terrestrial	Ground hunter	Diurnal	Resident	62.2
16	*Sclerurus mexicanus*	Passeriformes	Furnariidae	Insectivore	Terrestrial	Ground hunter	Diurnal	Resident	25
16	*Sclerurus guatemalensis*	Passeriformes	Furnariidae	Insectivore	Terrestrial	Ground hunter	Diurnal	Resident	34.7
16	*Microcerculus philomela*	Passeriformes	Troglodytidae	Insectivore	Terrestrial	Ground hunter	Diurnal	Resident	17.8
16	*Catherpes mexicanus*	Passeriformes	Troglodytidae	Insectivore	Terrestrial	Ground hunter	Diurnal	Resident	11.285
16	*Hylorchilus sumichrasti*	Passeriformes	Troglodytidae	Insectivore	Terrestrial	Ground hunter	Diurnal	Resident	28.4
16	*Hylorchilus navai*	Passeriformes	Troglodytidae	Insectivore	Terrestrial	Ground hunter	Diurnal	Resident	29.3
16	*Sialia sialis*	Passeriformes	Turdidae	Insectivore	Terrestrial	Ground hunter	Diurnal	Resident	31.6
16	*Catharus aurantiirostris*	Passeriformes	Turdidae	Insectivore	Terrestrial	Ground hunter	Diurnal	Resident	27
16	*Catharus occidentalis*	Passeriformes	Turdidae	Insectivore	Terrestrial	Ground hunter	Diurnal	Resident	26.2
16	*Catharus frantzii*	Passeriformes	Turdidae	Insectivore	Terrestrial	Ground hunter	Diurnal	Resident	31.4
16	*Catharus mexicanus*	Passeriformes	Turdidae	Insectivore	Terrestrial	Ground hunter	Diurnal	Resident	33
16	*Catharus dryas*	Passeriformes	Turdidae	Insectivore	Terrestrial	Ground hunter	Diurnal	Resident	35.1
16	*Turdus grayi*	Passeriformes	Turdidae	Insectivore	Terrestrial	Ground hunter	Diurnal	Resident	73.8
16	*Turdus assimilis*	Passeriformes	Turdidae	Insectivore	Terrestrial	Ground hunter	Diurnal	Resident	67.5
16	*Turdus rufopalliatus*	Passeriformes	Turdidae	Insectivore	Terrestrial	Ground hunter	Diurnal	Resident	76.8
16	*Turdus rufitorques*	Passeriformes	Turdidae	Insectivore	Terrestrial	Ground hunter	Diurnal	Resident	72
16	*Turdus migratorius*	Passeriformes	Turdidae	Insectivore	Terrestrial	Ground hunter	Diurnal	Resident	77.3
16	*Melanoptila glabrirostris*	Passeriformes	Mimidae	Insectivore	Terrestrial	Ground hunter	Diurnal	Resident	36.3
16	*Mimus polyglottos*	Passeriformes	Mimidae	Insectivore	Terrestrial	Ground hunter	Diurnal	Resident	48.45
16	*Mimus gilvus*	Passeriformes	Mimidae	Insectivore	Terrestrial	Ground hunter	Diurnal	Resident	58.4
16	*Mimus graysoni*	Passeriformes	Mimidae	Insectivore	Terrestrial	Ground hunter	Diurnal	Resident	66.64
16	*Toxostoma longirostre*	Passeriformes	Mimidae	Insectivore	Terrestrial	Ground hunter	Diurnal	Resident	69.9
16	*Toxostoma cinereum*	Passeriformes	Mimidae	Insectivore	Terrestrial	Ground hunter	Diurnal	Resident	59.15
16	*Toxostoma bendirei*	Passeriformes	Mimidae	Insectivore	Terrestrial	Ground hunter	Diurnal	Resident	62.2
16	*Toxostoma ocellatum*	Passeriformes	Mimidae	Insectivore	Terrestrial	Ground hunter	Diurnal	Resident	84.3
16	*Toxostoma curvirostre*	Passeriformes	Mimidae	Insectivore	Terrestrial	Ground hunter	Diurnal	Resident	79.4
16	*Toxostoma redivivum*	Passeriformes	Mimidae	Insectivore	Terrestrial	Ground hunter	Diurnal	Resident	84.4
16	*Toxostoma crissale*	Passeriformes	Mimidae	Insectivore	Terrestrial	Ground hunter	Diurnal	Resident	62.7
16	*Toxostoma lecontei*	Passeriformes	Mimidae	Insectivore	Terrestrial	Ground hunter	Diurnal	Resident	61.82
16	*Toxostoma guttatum*	Passeriformes	Mimidae	Insectivore	Terrestrial	Ground hunter	Diurnal	Resident	55
16	*Melanotis caerulescens*	Passeriformes	Mimidae	Insectivore	Terrestrial	Ground hunter	Diurnal	Resident	63.1
16	*Melanotis hypoleucus*	Passeriformes	Mimidae	Insectivore	Terrestrial	Ground hunter	Diurnal	Resident	62
16	*Anthus cervinus*	Passeriformes	Motacillidae	Insectivore	Terrestrial	Ground hunter	Diurnal	Resident	20.4
16	*Seiurus aurocapilla*	Passeriformes	Parulidae	Insectivore	Terrestrial	Ground hunter	Diurnal	Resident	22.1

16	*Melozone leucotis*	Passeriformes	Emberizidae	Insectivore	Terrestrial	Ground hunter	Diurnal	Resident	42.8
16	*Melozone aberti*	Passeriformes	Emberizidae	Insectivore	Terrestrial	Ground hunter	Diurnal	Resident	45.95
16	*Peucaea botterii*	Passeriformes	Emberizidae	Insectivore	Terrestrial	Ground hunter	Diurnal	Resident	20.8
16	*Amphispizopsis quinquestriata*	Passeriformes	Emberizidae	Insectivore	Terrestrial	Ground hunter	Diurnal	Resident	20
16	*Amphispiza bilineata*	Passeriformes	Emberizidae	Insectivore	Terrestrial	Ground hunter	Diurnal	Resident	13.6
16	*Agelaius tricolor*	Passeriformes	Icteridae	Insectivore	Terrestrial	Ground hunter	Diurnal	Resident	58.7
16	*Sturnella magna*	Passeriformes	Icteridae	Insectivore	Terrestrial	Ground hunter	Diurnal	Resident	89
16	*Dives dives*	Passeriformes	Icteridae	Insectivore	Terrestrial	Ground hunter	Diurnal	Resident	96.2
17	*Chordeiles acutipennis*	Caprimulgiformes	Caprimulgidae	Insectivore	Volant	Air hunter under canopy	Cathemeral	Resident	49.9
17	*Chordeiles minor*	Caprimulgiformes	Caprimulgidae	Insectivore	Volant	Air hunter under canopy	Diurnal	Resident	70.4
17	*Antrostomus salvini*	Caprimulgiformes	Caprimulgidae	Insectivore	Volant	Air hunter under canopy	Nocturnal	Resident	59
17	*Antrostomus badius*	Caprimulgiformes	Caprimulgidae	Insectivore	Volant	Air hunter under canopy	Nocturnal	Resident	64.3
17	*Antrostomus ridgwayi*	Caprimulgiformes	Caprimulgidae	Insectivore	Volant	Air hunter under canopy	Nocturnal	Resident	48
17	*Antrostomus arizonae*	Caprimulgiformes	Caprimulgidae	Insectivore	Volant	Air hunter under canopy	Nocturnal	Resident	52
17	*Hylomanes momotula*	Coraciiformes	Momotidae	Insectivore	Volant	Air hunter under canopy	Diurnal	Resident	29.3
17	*Aspatha gularis*	Coraciiformes	Momotidae	Insectivore	Volant	Air hunter under canopy	Diurnal	Resident	60.9
17	*Momotus mexicanus*	Coraciiformes	Momotidae	Insectivore	Volant	Air hunter under canopy	Diurnal	Resident	75.7
17	*Galbula ruficauda*	Piciformes	Galbulidae	Insectivore	Volant	Air hunter under canopy	Diurnal	Resident	26.5
17	*Camptostoma imberbe*	Passeriformes	Tyrannidae	Insectivore	Volant	Air hunter under canopy	Diurnal	Resident	7.4
17	*Elaenia martinica*	Passeriformes	Tyrannidae	Insectivore	Volant	Air hunter under canopy	Diurnal	Resident	22.6
17	*Elaenia flavogaster*	Passeriformes	Tyrannidae	Insectivore	Volant	Air hunter under canopy	Diurnal	Resident	24.7
17	*Mionectes oleagineus*	Passeriformes	Tyrannidae	Insectivore	Volant	Air hunter under canopy	Diurnal	Resident	12.1
17	*Leptopogon amaurocephalus*	Passeriformes	Tyrannidae	Insectivore	Volant	Air hunter under canopy	Diurnal	Resident	11.7
17	*Todirostrum cinereum*	Passeriformes	Tyrannidae	Insectivore	Volant	Air hunter under canopy	Diurnal	Resident	6.4
17	*Tolmomyias sulphurescens*	Passeriformes	Tyrannidae	Insectivore	Volant	Air hunter under canopy	Diurnal	Resident	14.9
17	*Onychorhynchus coronatus*	Passeriformes	Tyrannidae	Insectivore	Volant	Air hunter under canopy	Diurnal	Resident	14
17	*Terenotriccus erythrurus*	Passeriformes	Tyrannidae	Insectivore	Volant	Air hunter under canopy	Diurnal	Resident	7.5
17	*Myiobius sulphureipygius*	Passeriformes	Tyrannidae	Insectivore	Volant	Air hunter under canopy	Diurnal	Resident	13.5
17	*Mitrephanes phaeocercus*	Passeriformes	Tyrannidae	Insectivore	Volant	Air hunter under canopy	Diurnal	Resident	8.6
17	*Contopus pertinax*	Passeriformes	Tyrannidae	Insectivore	Volant	Air hunter under canopy	Diurnal	Resident	27.2
17	*Contopus sordidulus*	Passeriformes	Tyrannidae	Insectivore	Volant	Air hunter under canopy	Diurnal	Resident	12.5
17	*Contopus cinereus*	Passeriformes	Tyrannidae	Insectivore	Volant	Air hunter under canopy	Diurnal	Resident	11.7
17	*Empidonax occidentalis*	Passeriformes	Tyrannidae	Insectivore	Volant	Air hunter under canopy	Diurnal	Resident	11.4
17	*Pyrocephalus rubinus*	Passeriformes	Tyrannidae	Insectivore	Volant	Air hunter under canopy	Diurnal	Resident	14.4
17	*Myiarchus yucatanensis*	Passeriformes	Tyrannidae	Insectivore	Volant	Air hunter under canopy	Diurnal	Resident	21.4
17	*Myiarchus tuberculifer*	Passeriformes	Tyrannidae	Insectivore	Volant	Air hunter under canopy	Diurnal	Resident	20
17	*Myiarchus nuttingi*	Passeriformes	Tyrannidae	Insectivore	Volant	Air hunter under canopy	Diurnal	Resident	23
17	*Myiarchus tyrannulus*	Passeriformes	Tyrannidae	Insectivore	Volant	Air hunter under canopy	Diurnal	Resident	37.15
17	*Ramphotrigon flammulatum*	Passeriformes	Tyrannidae	Insectivore	Volant	Air hunter under canopy	Diurnal	Resident	17.2
17	*Megarynchus pitangua*	Passeriformes	Tyrannidae	Insectivore	Volant	Air hunter under canopy	Diurnal	Resident	73.5
17	*Myiozetetes similis*	Passeriformes	Tyrannidae	Insectivore	Volant	Air hunter under canopy	Diurnal	Resident	28
17	*Myiodynastes maculatus*	Passeriformes	Tyrannidae	Insectivore	Volant	Air hunter under canopy	Diurnal	Resident	45.9
17	*Myiodynastes luteiventris*	Passeriformes	Tyrannidae	Insectivore	Volant	Air hunter under canopy	Diurnal	Resident	47.1
17	*Legatus leucophaius*	Passeriformes	Tyrannidae	Insectivore	Volant	Air hunter under canopy	Diurnal	Resident	24.4
17	*Tyrannus melancholicus*	Passeriformes	Tyrannidae	Insectivore	Volant	Air hunter under canopy	Diurnal	Resident	37.55
17	*Tyrannus couchii*	Passeriformes	Tyrannidae	Insectivore	Volant	Air hunter under canopy	Diurnal	Resident	45
17	*Tyrannus crassirostris*	Passeriformes	Tyrannidae	Insectivore	Volant	Air hunter under canopy	Diurnal	Resident	55.65
17	*Tyrannus savana*	Passeriformes	Tyrannidae	Insectivore	Volant	Air hunter under canopy	Diurnal	Resident	28.6

17	*Pachyramphus cinnamomeus*	Passeriformes	Tityridae	Insectivore	Volant	Air hunter under canopy	Diurnal	Resident	20.3
17	*Pachyramphus major*	Passeriformes	Tityridae	Insectivore	Volant	Air hunter under canopy	Diurnal	Resident	25.2
17	*Pachyramphus aglaiae*	Passeriformes	Tityridae	Insectivore	Volant	Air hunter under canopy	Diurnal	Resident	30
17	*Pachyramphus polychopterus*	Passeriformes	Tityridae	Insectivore	Volant	Air hunter under canopy	Diurnal	Resident	20
17	*Schiffornis veraepacis*	Passeriformes	Tityridae	Insectivore	Volant	Air hunter under canopy	Diurnal	Resident	26
17	*Myadestes occidentalis*	Passeriformes	Turdidae	Insectivore	Volant	Air hunter under canopy	Diurnal	Resident	41.6
17	*Myadestes unicolor*	Passeriformes	Turdidae	Insectivore	Volant	Air hunter under canopy	Diurnal	Resident	36.7
17	*Phainopepla nitens*	Passeriformes	Ptilogonatidae	Insectivore	Volant	Air hunter under canopy	Diurnal	Resident	24
18	*Contopus virens*	Passeriformes	Tyrannidae	Insectivore	Volant	Air hunter under canopy	Diurnal	Transient	14.1
18	*Empidonax virescens*	Passeriformes	Tyrannidae	Insectivore	Volant	Air hunter under canopy	Diurnal	Transient	12
18	*Empidonax alnorum*	Passeriformes	Tyrannidae	Insectivore	Volant	Air hunter under canopy	Diurnal	Transient	13.25
18	*Tyrannus tyrannus*	Passeriformes	Tyrannidae	Insectivore	Volant	Air hunter under canopy	Diurnal	Transient	43.6
18	*Tyrannus dominicensis*	Passeriformes	Tyrannidae	Insectivore	Volant	Air hunter under canopy	Diurnal	Transient	43.8
18	*Elaenia frantzii*	Passeriformes	Tyrannidae	Insectivore	Volant	Air hunter under canopy	Diurnal	Accidental	15
19	*Antrostomus carolinensis*	Caprimulgiformes	Caprimulgidae	Insectivore	Volant	Air hunter under canopy	Nocturnal	Winter migrant	108.9
19	*Antrostomus vociferus*	Caprimulgiformes	Caprimulgidae	Insectivore	Volant	Air hunter under canopy	Nocturnal	Winter migrant	52.95
19	*Melanerpes lewis*	Piciformes	Picidae	Insectivore	Volant	Air hunter under canopy	Diurnal	Winter migrant	105.8
19	*Contopus cooperi*	Passeriformes	Tyrannidae	Insectivore	Volant	Air hunter under canopy	Diurnal	Winter migrant	33.2
19	*Empidonax flaviventris*	Passeriformes	Tyrannidae	Insectivore	Volant	Air hunter under canopy	Diurnal	Winter migrant	11.45
19	*Empidonax traillii*	Passeriformes	Tyrannidae	Insectivore	Volant	Air hunter under canopy	Diurnal	Winter migrant	13.4
19	*Empidonax albigularis*	Passeriformes	Tyrannidae	Insectivore	Volant	Air hunter under canopy	Diurnal	Winter migrant	10.8
19	*Empidonax minimus*	Passeriformes	Tyrannidae	Insectivore	Volant	Air hunter under canopy	Diurnal	Winter migrant	10.55
19	*Empidonax hammondii*	Passeriformes	Tyrannidae	Insectivore	Volant	Air hunter under canopy	Diurnal	Winter migrant	10.445
19	*Empidonax wrightii*	Passeriformes	Tyrannidae	Insectivore	Volant	Air hunter under canopy	Diurnal	Winter migrant	12.5
19	*Empidonax oberholseri*	Passeriformes	Tyrannidae	Insectivore	Volant	Air hunter under canopy	Diurnal	Winter migrant	10.95
19	*Empidonax affinis*	Passeriformes	Tyrannidae	Insectivore	Volant	Air hunter under canopy	Diurnal	Winter migrant	11.3
19	*Empidonax difficilis*	Passeriformes	Tyrannidae	Insectivore	Volant	Air hunter under canopy	Diurnal	Winter migrant	10.665
19	*Empidonax flavescens*	Passeriformes	Tyrannidae	Insectivore	Volant	Air hunter under canopy	Diurnal	Winter migrant	12.2
19	*Empidonax fulvifrons*	Passeriformes	Tyrannidae	Insectivore	Volant	Air hunter under canopy	Diurnal	Winter migrant	7.9
19	*Sayornis nigricans*	Passeriformes	Tyrannidae	Insectivore	Volant	Air hunter under canopy	Diurnal	Winter migrant	18.25
19	*Sayornis phoebe*	Passeriformes	Tyrannidae	Insectivore	Volant	Air hunter under canopy	Diurnal	Winter migrant	18.3
19	*Sayornis saya*	Passeriformes	Tyrannidae	Insectivore	Volant	Air hunter under canopy	Diurnal	Winter migrant	20.8
19	*Myiarchus cinerascens*	Passeriformes	Tyrannidae	Insectivore	Volant	Air hunter under canopy	Diurnal	Winter migrant	27.95
19	*Myiarchus crinitus*	Passeriformes	Tyrannidae	Insectivore	Volant	Air hunter under canopy	Diurnal	Winter migrant	33.5
19	*Tyrannus vociferans*	Passeriformes	Tyrannidae	Insectivore	Volant	Air hunter under canopy	Diurnal	Winter migrant	45.6
19	*Tyrannus verticalis*	Passeriformes	Tyrannidae	Insectivore	Volant	Air hunter under canopy	Diurnal	Winter migrant	37.9
19	*Tyrannus forficatus*	Passeriformes	Tyrannidae	Insectivore	Volant	Air hunter under canopy	Diurnal	Winter migrant	43.2
19	*Sialia mexicana*	Passeriformes	Turdidae	Insectivore	Volant	Air hunter under canopy	Diurnal	Winter migrant	28.05
19	*Sialia currucoides*	Passeriformes	Turdidae	Insectivore	Volant	Air hunter under canopy	Diurnal	Winter migrant	29.6
19	*Myadestes townsendi*	Passeriformes	Turdidae	Insectivore	Volant	Air hunter under canopy	Diurnal	Winter migrant	32.5
19	*Setophaga ruticilla*	Passeriformes	Parulidae	Insectivore	Volant	Air hunter under canopy	Diurnal	Winter migrant	8.3
20	*Ictinia mississippiensis*	Accipitriformes	Accipitridae	Insectivore	Volant	Air hunter above canopy	Diurnal	Transient	278
20	*Ictinia plumbea*	Accipitriformes	Accipitridae	Insectivore	Volant	Air hunter above canopy	Diurnal	Resident	263.5
20	*Cypseloides niger*	Apodiformes	Apodidae	Insectivore	Volant	Air hunter above canopy	Diurnal	Resident	42.15
20	*Cypseloides storeri*	Apodiformes	Apodidae	Insectivore	Volant	Air hunter above canopy	Diurnal	Resident	39.5
20	*Streptoprocne rutila*	Apodiformes	Apodidae	Insectivore	Volant	Air hunter above canopy	Diurnal	Resident	20.2
20	*Streptoprocne zonaris*	Apodiformes	Apodidae	Insectivore	Volant	Air hunter above canopy	Diurnal	Resident	98.1
20	*Streptoprocne semicollaris*	Apodiformes	Apodidae	Insectivore	Volant	Air hunter above canopy	Diurnal	Resident	175

20	*Chaetura pelagica*	Apodiformes	Apodidae	Insectivore	Volant	Air hunter above canopy	Diurnal	Transient	23.6
20	*Chaetura vauxi*	Apodiformes	Apodidae	Insectivore	Volant	Air hunter above canopy	Diurnal	Resident	18
20	*Aeronautes saxatalis*	Apodiformes	Apodidae	Insectivore	Volant	Air hunter above canopy	Diurnal	Resident	36.8
20	*Panyptila cayennensis*	Apodiformes	Apodidae	Insectivore	Volant	Air hunter above canopy	Diurnal	Resident	21.1
20	*Panyptila sanctihieronymi*	Apodiformes	Apodidae	Insectivore	Volant	Air hunter above canopy	Diurnal	Resident	48.1
20	*Pygochelidon cyanoleuca*	Passeriformes	Hirundinidae	Insectivore	Volant	Air hunter above canopy	Diurnal	Resident	12
20	*Progne tapera*	Passeriformes	Hirundinidae	Insectivore	Volant	Air hunter above canopy	Diurnal	Accidental	22
20	*Progne subis*	Passeriformes	Hirundinidae	Insectivore	Volant	Air hunter above canopy	Diurnal	Resident	53.23
20	*Progne sinaloae*	Passeriformes	Hirundinidae	Insectivore	Volant	Air hunter above canopy	Diurnal	Resident	42.6
20	*Progne chalybea*	Passeriformes	Hirundinidae	Insectivore	Volant	Air hunter above canopy	Diurnal	Resident	42.9
20	*Tachycineta bicolor*	Passeriformes	Hirundinidae	Insectivore	Volant	Air hunter above canopy	Diurnal	Winter migrant	20.1
20	*Tachycineta albilinea*	Passeriformes	Hirundinidae	Insectivore	Volant	Air hunter above canopy	Diurnal	Resident	13.9
20	*Tachycineta thalassina*	Passeriformes	Hirundinidae	Insectivore	Volant	Air hunter above canopy	Diurnal	Winter migrant	14.15
20	*Atticora pileata*	Passeriformes	Hirundinidae	Insectivore	Volant	Air hunter above canopy	Diurnal	Resident	12.2
20	*Stelgidopteryx serripennis*	Passeriformes	Hirundinidae	Insectivore	Volant	Air hunter above canopy	Diurnal	Winter migrant	15.9
20	*Riparia riparia*	Passeriformes	Hirundinidae	Insectivore	Volant	Air hunter above canopy	Diurnal	Winter migrant	13.45
20	*Petrochelidon pyrrhonota*	Passeriformes	Hirundinidae	Insectivore	Volant	Air hunter above canopy	Diurnal	Resident	21.3
20	*Petrochelidon fulva*	Passeriformes	Hirundinidae	Insectivore	Volant	Air hunter above canopy	Diurnal	Resident	15.8
20	*Hirundo rustica*	Passeriformes	Hirundinidae	Insectivore	Volant	Air hunter above canopy	Diurnal	Winter migrant	18.65
20	*Ptiliogonys cinereus*	Passeriformes	Ptilogonatidae	Insectivore	Volant	Air hunter above canopy	Diurnal	Resident	33.6
21	*Megascops kennicottii*	Strigiformes	Strigidae	Insectivore	Volant	Ground hunter	Nocturnal	Resident	120.9
21	*Megascops asio*	Strigiformes	Strigidae	Insectivore	Volant	Ground hunter	Nocturnal	Resident	171
21	*Megascops seductus*	Strigiformes	Strigidae	Insectivore	Volant	Ground hunter	Nocturnal	Resident	160
21	*Megascops cooperi*	Strigiformes	Strigidae	Insectivore	Volant	Ground hunter	Nocturnal	Resident	149
21	*Megascops trichopsis*	Strigiformes	Strigidae	Insectivore	Volant	Ground hunter	Nocturnal	Resident	91.5
21	*Megascops barbarus*	Strigiformes	Strigidae	Insectivore	Volant	Ground hunter	Nocturnal	Resident	69
21	*Megascops guatemalae*	Strigiformes	Strigidae	Insectivore	Volant	Ground hunter	Nocturnal	Resident	107
21	*Lophostrix cristata*	Strigiformes	Strigidae	Insectivore	Volant	Ground hunter	Nocturnal	Resident	544
21	*Lurocalis semitorquatus*	Caprimulgiformes	Caprimulgidae	Insectivore	Volant	Ground hunter	Nocturnal	Resident	87
21	*Nyctibius grandis*	Caprimulgiformes	Nyctibiidae	Insectivore	Volant	Ground hunter	Nocturnal	Resident	273.5
21	*Nyctibius jamaicensis*	Caprimulgiformes	Nyctibiidae	Insectivore	Volant	Ground hunter	Nocturnal	Resident	251
22	*Rupornis magnirostris*	Accipitriformes	Accipitridae	Insectivore	Volant	Ground hunter	Diurnal	Resident	279.5
22	*Psiloscops flammeolus*	Strigiformes	Strigidae	Insectivore	Volant	Ground hunter	Nocturnal	Winter migrant	55.55
22	*Glaucidium griseiceps*	Strigiformes	Strigidae	Insectivore	Volant	Ground hunter	Cathemeral	Resident	61.9
22	*Micrathene whitneyi*	Strigiformes	Strigidae	Insectivore	Volant	Ground hunter	Diurnal	Winter migrant	29.5
22	*Athene cunicularia*	Strigiformes	Strigidae	Insectivore	Volant	Ground hunter	Cathemeral	Winter migrant	155
22	*Hydropsalis maculicaudus*	Caprimulgiformes	Caprimulgidae	Insectivore	Volant	Ground hunter	Diurnal	Resident	30.3
22	*Momotus coeruliceps*	Coraciiformes	Momotidae	Insectivore	Volant	Ground hunter	Diurnal	Resident	123
22	*Momotus lessonii*	Coraciiformes	Momotidae	Insectivore	Volant	Ground hunter	Diurnal	Resident	123
22	*Eumomota superciliosa*	Coraciiformes	Momotidae	Insectivore	Volant	Ground hunter	Diurnal	Resident	62.5
22	*Falco sparverius*	Falconiformes	Falconidae	Insectivore	Volant	Ground hunter	Diurnal	Winter migrant	115.5
22	*Attila spadiceus*	Passeriformes	Tyrannidae	Insectivore	Volant	Ground hunter	Diurnal	Resident	39.1
22	*Lanius ludovicianus*	Passeriformes	Laniidae	Insectivore	Volant	Ground hunter	Diurnal	Winter migrant	47.55
23	*Anser albifrons*	Anseriformes	Anatidae	Herbivore	Semi-aquatic	Ground gleaner	Diurnal	Winter migrant	2486
23	*Anser anser*	Anseriformes	Anatidae	Herbivore	Semi-aquatic	Ground gleaner	Diurnal	Winter migrant	3308.5
23	*Anser caerulescens*	Anseriformes	Anatidae	Herbivore	Semi-aquatic	Ground gleaner	Diurnal	Winter migrant	2548.3
23	*Anser rossii*	Anseriformes	Anatidae	Herbivore	Semi-aquatic	Ground gleaner	Diurnal	Winter migrant	1347.5
23	*Branta bernicla*	Anseriformes	Anatidae	Herbivore	Semi-aquatic	Ground gleaner	Diurnal	Winter migrant	1725

23	*Branta hutchinsii*	Anseriformes	Anatidae	Herbivore	Semi-aquatic	Ground gleaner	Diurnal	Winter migrant	2050
23	*Branta canadensis*	Anseriformes	Anatidae	Herbivore	Semi-aquatic	Ground gleaner	Diurnal	Winter migrant	4841.65
24	*Callipepla gambelii*	Galliformes	Odontophoridae	Herbivore	Terrestrial	Ground gleaner	Diurnal	Resident	169.25
24	*Meleagris gallopavo*	Galliformes	Phasianidae	Herbivore	Terrestrial	Ground gleaner	Diurnal	Resident	3911
24	*Meleagris ocellata*	Galliformes	Phasianidae	Herbivore	Terrestrial	Ground gleaner	Diurnal	Resident	5525
25	*Antigone canadensis*	Gruiformes	Gruidae	Omnivore	Semi-aquatic	Aquatic surface gleaner	Diurnal	Winter migrant	4200
25	*Grus americana*	Gruiformes	Gruidae	Omnivore	Semi-aquatic	Aquatic surface gleaner	Diurnal	Winter migrant	7496.5
26	*Cairina moschata*	Anseriformes	Anatidae	Herbivore	Semi-aquatic	Aquatic surface gleaner	Diurnal	Resident	2117.5
26	*Phoenicopterus ruber*	Phoenicopteriformes	Phoenicopteridae	Herbivore	Semi-aquatic	Aquatic surface gleaner	Diurnal	Resident	3035
26	*Porzana carolina*	Gruiformes	Rallidae	Herbivore	Semi-aquatic	Aquatic surface gleaner	Diurnal	Resident	80.775
26	*Hapalocrex flaviventer*	Gruiformes	Rallidae	Herbivore	Semi-aquatic	Aquatic surface gleaner	Diurnal	Resident	24.95
26	*Porphyrio martinicus*	Gruiformes	Rallidae	Herbivore	Semi-aquatic	Aquatic surface gleaner	Diurnal	Resident	218.45
26	*Gallinula galeata*	Gruiformes	Rallidae	Herbivore	Semi-aquatic	Aquatic surface gleaner	Diurnal	Resident	382
26	*Fulica americana*	Gruiformes	Rallidae	Herbivore	Semi-aquatic	Aquatic surface gleaner	Diurnal	Resident	642
27	*Aix sponsa*	Anseriformes	Anatidae	Herbivore	Semi-aquatic	Aquatic surface gleaner	Diurnal	Winter migrant	673
27	*Mareca strepera*	Anseriformes	Anatidae	Herbivore	Semi-aquatic	Aquatic surface gleaner	Cathemeral	Winter migrant	858
27	*Mareca americana*	Anseriformes	Anatidae	Herbivore	Semi-aquatic	Aquatic surface gleaner	Diurnal	Winter migrant	780.2
27	*Anas platyrhynchos*	Anseriformes	Anatidae	Herbivore	Semi-aquatic	Aquatic surface gleaner	Diurnal	Winter migrant	1189
27	*Spatula cyanoptera*	Anseriformes	Anatidae	Herbivore	Semi-aquatic	Aquatic surface gleaner	Diurnal	Winter migrant	370
27	*Aythya valisineria*	Anseriformes	Anatidae	Herbivore	Semi-aquatic	Aquatic surface gleaner	Cathemeral	Winter migrant	1273.1
27	*Aythya americana*	Anseriformes	Anatidae	Herbivore	Semi-aquatic	Aquatic surface gleaner	Cathemeral	Winter migrant	928
27	*Aythya collaris*	Anseriformes	Anatidae	Herbivore	Semi-aquatic	Aquatic surface gleaner	Diurnal	Winter migrant	727.2
27	*Aythya marila*	Anseriformes	Anatidae	Herbivore	Semi-aquatic	Aquatic surface gleaner	Cathemeral	Winter migrant	1015
28	*Cygnus buccinator*	Anseriformes	Anatidae	Herbivore	Semi-aquatic	Aquatic surface gleaner	Cathemeral	Accidental	1080
28	*Cygnus columbianus*	Anseriformes	Anatidae	Herbivore	Semi-aquatic	Aquatic surface gleaner	Diurnal	Accidental	6750
28	*Mareca penelope*	Anseriformes	Anatidae	Herbivore	Semi-aquatic	Aquatic surface gleaner	Nocturnal	Accidental	650
29	*Charadrius nivosus*	Charadriiformes	Charadriidae	Piscivore	Semi-aquatic	Wader	Cathemeral	Winter migrant	42.25
29	*Charadrius wilsonia*	Charadriiformes	Charadriidae	Piscivore	Semi-aquatic	Wader	Cathemeral	Winter migrant	67.5
29	*Charadrius semipalmatus*	Charadriiformes	Charadriidae	Piscivore	Semi-aquatic	Wader	Cathemeral	Winter migrant	46.95
29	*Charadrius melodus*	Charadriiformes	Charadriidae	Piscivore	Semi-aquatic	Wader	Cathemeral	Winter migrant	55.2
29	*Charadrius vociferus*	Charadriiformes	Charadriidae	Piscivore	Semi-aquatic	Wader	Cathemeral	Winter migrant	121.8
29	*Charadrius morinellus*	Charadriiformes	Charadriidae	Piscivore	Semi-aquatic	Wader	Cathemeral	Winter migrant	120
29	*Himantopus mexicanus*	Charadriiformes	Recurvirostridae	Piscivore	Semi-aquatic	Wader	Cathemeral	Resident	169.55
29	*Tringa semipalmata*	Charadriiformes	Scolopacidae	Piscivore	Semi-aquatic	Wader	Cathemeral	Winter migrant	251
29	*Limosa lapponica*	Charadriiformes	Scolopacidae	Piscivore	Semi-aquatic	Wader	Cathemeral	Transient	486
29	*Limosa fedoa*	Charadriiformes	Scolopacidae	Piscivore	Semi-aquatic	Wader	Cathemeral	Winter migrant	358.5
29	*Calidris alpina*	Charadriiformes	Scolopacidae	Piscivore	Semi-aquatic	Wader	Cathemeral	Winter migrant	55.6
30	*Pluvialis squatarola*	Charadriiformes	Charadriidae	Piscivore	Semi-aquatic	Wader	Diurnal	Winter migrant	250
30	*Pluvialis fulva*	Charadriiformes	Charadriidae	Piscivore	Semi-aquatic	Wader	Diurnal	Winter migrant	145.5
30	*Charadrius montanus*	Charadriiformes	Charadriidae	Piscivore	Semi-aquatic	Wader	Diurnal	Winter migrant	101.42
30	*Recurvirostra americana*	Charadriiformes	Recurvirostridae	Piscivore	Semi-aquatic	Wader	Diurnal	Winter migrant	264.9
30	*Actitis macularius*	Charadriiformes	Scolopacidae	Piscivore	Semi-aquatic	Wader	Diurnal	Winter migrant	38.7
30	*Tringa solitaria*	Charadriiformes	Scolopacidae	Piscivore	Semi-aquatic	Wader	Diurnal	Winter migrant	48.4
30	*Tringa incana*	Charadriiformes	Scolopacidae	Piscivore	Semi-aquatic	Wader	Diurnal	Winter migrant	115.8
30	*Tringa melanoleuca*	Charadriiformes	Scolopacidae	Piscivore	Semi-aquatic	Wader	Diurnal	Winter migrant	170
30	*Tringa flavipes*	Charadriiformes	Scolopacidae	Piscivore	Semi-aquatic	Wader	Diurnal	Winter migrant	77.25
30	*Numenius borealis*	Charadriiformes	Scolopacidae	Piscivore	Semi-aquatic	Wader	Diurnal	Winter migrant	375
30	*Numenius phaeopus*	Charadriiformes	Scolopacidae	Piscivore	Semi-aquatic	Wader	Diurnal	Winter migrant	404

30	*Numenius americanus*	Charadriiformes	Scolopacidae	Piscivore	Semi-aquatic	Wader	Diurnal	Winter migrant	754.5
30	*Arenaria interpres*	Charadriiformes	Scolopacidae	Piscivore	Semi-aquatic	Wader	Diurnal	Winter migrant	107.45
30	*Arenaria melanocephala*	Charadriiformes	Scolopacidae	Piscivore	Semi-aquatic	Wader	Diurnal	Winter migrant	126.5
30	*Calidris virgata*	Charadriiformes	Scolopacidae	Piscivore	Semi-aquatic	Wader	Diurnal	Winter migrant	211.3
30	*Calidris canutus*	Charadriiformes	Scolopacidae	Piscivore	Semi-aquatic	Wader	Diurnal	Winter migrant	124.9
30	*Calidris alba*	Charadriiformes	Scolopacidae	Piscivore	Semi-aquatic	Wader	Diurnal	Winter migrant	63.7
30	*Calidris pusilla*	Charadriiformes	Scolopacidae	Piscivore	Semi-aquatic	Wader	Diurnal	Winter migrant	26.15
30	*Calidris mauri*	Charadriiformes	Scolopacidae	Piscivore	Semi-aquatic	Wader	Diurnal	Winter migrant	27.2
30	*Calidris minuta*	Charadriiformes	Scolopacidae	Piscivore	Semi-aquatic	Wader	Diurnal	Winter migrant	23.75
30	*Calidris minutilla*	Charadriiformes	Scolopacidae	Piscivore	Semi-aquatic	Wader	Diurnal	Winter migrant	23.45
30	*Calidris fuscicollis*	Charadriiformes	Scolopacidae	Piscivore	Semi-aquatic	Wader	Diurnal	Winter migrant	44.55
30	*Calidris bairdii*	Charadriiformes	Scolopacidae	Piscivore	Semi-aquatic	Wader	Diurnal	Winter migrant	46
30	*Calidris melanotos*	Charadriiformes	Scolopacidae	Piscivore	Semi-aquatic	Wader	Diurnal	Winter migrant	75.9
30	*Calidris ferruginea*	Charadriiformes	Scolopacidae	Piscivore	Semi-aquatic	Wader	Diurnal	Winter migrant	69
30	*Calidris himantopus*	Charadriiformes	Scolopacidae	Piscivore	Semi-aquatic	Wader	Diurnal	Winter migrant	57.35
30	*Calidris subruficollis*	Charadriiformes	Scolopacidae	Piscivore	Semi-aquatic	Wader	Diurnal	Winter migrant	62.4
30	*Calidris acuminata*	Charadriiformes	Scolopacidae	Piscivore	Semi-aquatic	Wader	Diurnal	Winter migrant	76.5
30	*Calidris maritima*	Charadriiformes	Scolopacidae	Piscivore	Semi-aquatic	Wader	Diurnal	Winter migrant	65
30	*Limnodromus griseus*	Charadriiformes	Scolopacidae	Piscivore	Semi-aquatic	Wader	Diurnal	Winter migrant	117.5
30	*Limnodromus scolopaceus*	Charadriiformes	Scolopacidae	Piscivore	Semi-aquatic	Wader	Diurnal	Winter migrant	108.5
30	*Gallinago delicata*	Charadriiformes	Scolopacidae	Piscivore	Semi-aquatic	Wader	Diurnal	Winter migrant	97.05
30	*Phalaropus lobatus*	Charadriiformes	Scolopacidae	Piscivore	Semi-aquatic	Wader	Diurnal	Winter migrant	36.8
30	*Phalaropus fulicarius*	Charadriiformes	Scolopacidae	Piscivore	Semi-aquatic	Wader	Diurnal	Winter migrant	55.95
31	*Pluvialis dominica*	Charadriiformes	Charadriidae	Piscivore	Semi-aquatic	Wader	Diurnal	Transient	154.75
31	*Charadrius collaris*	Charadriiformes	Charadriidae	Piscivore	Semi-aquatic	Wader	Diurnal	Resident	35
31	*Haematopus palliatus*	Charadriiformes	Haematopodidae	Piscivore	Semi-aquatic	Wader	Diurnal	Resident	602.5
31	*Haematopus bachmani*	Charadriiformes	Haematopodidae	Piscivore	Semi-aquatic	Wader	Diurnal	Resident	554.9
31	*Tringa stagnatilis*	Charadriiformes	Scolopacidae	Piscivore	Semi-aquatic	Wader	Diurnal	Accidental	77.5
31	*Tringa glareola*	Charadriiformes	Scolopacidae	Piscivore	Semi-aquatic	Wader	Diurnal	Accidental	67.5
31	*Limosa haemastica*	Charadriiformes	Scolopacidae	Piscivore	Semi-aquatic	Wader	Diurnal	Transient	247.5
31	*Calidris pugnax*	Charadriiformes	Scolopacidae	Piscivore	Semi-aquatic	Wader	Diurnal	Accidental	136
31	*Phalaropus tricolor*	Charadriiformes	Scolopacidae	Piscivore	Semi-aquatic	Wader	Diurnal	Transient	66.25
32	*Rostrhamus sociabilis*	Accipitriformes	Accipitridae	Piscivore	Volant	Aquatic surface hunter	Diurnal	Resident	392.3
32	*Busarellus nigricollis*	Accipitriformes	Accipitridae	Piscivore	Volant	Aquatic surface hunter	Diurnal	Resident	650
32	*Buteogallus anthracinus*	Accipitriformes	Accipitridae	Piscivore	Volant	Aquatic surface hunter	Diurnal	Resident	869
32	*Megaceryle torquata*	Coraciiformes	Alcedinidae	Piscivore	Volant	Aquatic surface hunter	Diurnal	Resident	320
32	*Chloroceryle amazona*	Coraciiformes	Alcedinidae	Piscivore	Volant	Aquatic surface hunter	Diurnal	Resident	126.5
32	*Chloroceryle americana*	Coraciiformes	Alcedinidae	Piscivore	Volant	Aquatic surface hunter	Diurnal	Resident	32.05
32	*Chloroceryle aenea*	Coraciiformes	Alcedinidae	Piscivore	Volant	Aquatic surface hunter	Diurnal	Resident	14.9
33	*Pandion haliaetus*	Accipitriformes	Pandioninae	Piscivore	Volant	Aquatic surface hunter	Diurnal	Winter migrant	1700
33	*Haliaeetus leucocephalus*	Accipitriformes	Accipitridae	Piscivore	Volant	Aquatic surface hunter	Diurnal	Winter migrant	4790
33	*Megaceryle alcyon*	Coraciiformes	Alcedinidae	Piscivore	Volant	Aquatic surface hunter	Diurnal	Winter migrant	147.6
34	*Anas fulvigula*	Anseriformes	Anatidae	Piscivore	Semi-aquatic	Aquatic surface hunter	Diurnal	Resident	988.5
34	*Histrionicus histrionicus*	Anseriformes	Anatidae	Piscivore	Semi-aquatic	Aquatic surface hunter	Diurnal	Accidental	597.5
34	*Nomonyx dominicus*	Anseriformes	Anatidae	Piscivore	Semi-aquatic	Aquatic surface hunter	Diurnal	Resident	365
34	*Oxyura jamaicensis*	Anseriformes	Anatidae	Piscivore	Semi-aquatic	Aquatic surface hunter	Diurnal	Resident	563.5
34	*Tachybaptus dominicus*	Podicipediformes	Podicipedidae	Piscivore	Semi-aquatic	Aquatic surface hunter	Diurnal	Resident	122.5
34	*Podilymbus podiceps*	Podicipediformes	Podicipedidae	Piscivore	Semi-aquatic	Aquatic surface hunter	Diurnal	Resident	416

34	*Aechmophorus occidentalis*	Podicipediformes	Podicipedidae	Piscivore	Semi-aquatic	Aquatic surface hunter	Diurnal	Resident	997.5
34	*Aechmophorus clarkii*	Podicipediformes	Podicipedidae	Piscivore	Semi-aquatic	Aquatic surface hunter	Diurnal	Resident	1102.25
34	*Mycteria americana*	Ciconiiformes	Ciconiidae	Piscivore	Semi-aquatic	Aquatic surface hunter	Diurnal	Resident	2377.5
34	*Pelecanus occidentalis*	Pelecaniformes	Pelecanidae	Piscivore	Semi-aquatic	Aquatic surface hunter	Diurnal	Resident	3057
34	*Botaurus pinnatus*	Pelecaniformes	Ardeidae	Piscivore	Semi-aquatic	Aquatic surface hunter	Nocturnal	Resident	800
34	*Ixobrychus exilis*	Pelecaniformes	Ardeidae	Piscivore	Semi-aquatic	Aquatic surface hunter	Diurnal	Resident	86.3
34	*Tigrisoma mexicanum*	Pelecaniformes	Ardeidae	Piscivore	Semi-aquatic	Aquatic surface hunter	Diurnal	Resident	1160
34	*Ardea alba*	Pelecaniformes	Ardeidae	Piscivore	Semi-aquatic	Aquatic surface hunter	Diurnal	Resident	873.5
34	*Egretta thula*	Pelecaniformes	Ardeidae	Piscivore	Semi-aquatic	Aquatic surface hunter	Diurnal	Resident	369
34	*Egretta caerulea*	Pelecaniformes	Ardeidae	Piscivore	Semi-aquatic	Aquatic surface hunter	Diurnal	Resident	339.5
34	*Egretta tricolor*	Pelecaniformes	Ardeidae	Piscivore	Semi-aquatic	Aquatic surface hunter	Diurnal	Resident	415
34	*Egretta rufescens*	Pelecaniformes	Ardeidae	Piscivore	Semi-aquatic	Aquatic surface hunter	Diurnal	Resident	613.75
34	*Butorides virescens*	Pelecaniformes	Ardeidae	Piscivore	Semi-aquatic	Aquatic surface hunter	Diurnal	Resident	226.5
34	*Agamia agami*	Pelecaniformes	Ardeidae	Piscivore	Semi-aquatic	Aquatic surface hunter	Diurnal	Resident	550
34	*Nycticorax nycticorax*	Pelecaniformes	Ardeidae	Piscivore	Semi-aquatic	Aquatic surface hunter	Diurnal	Resident	869.8
34	*Nyctanassa violacea*	Pelecaniformes	Ardeidae	Piscivore	Semi-aquatic	Aquatic surface hunter	Diurnal	Resident	682.5
34	*Cochlearius cochlearius*	Pelecaniformes	Ardeidae	Piscivore	Semi-aquatic	Aquatic surface hunter	Cathemeral	Resident	600
34	*Eudocimus albus*	Pelecaniformes	Threskiornithidae	Piscivore	Semi-aquatic	Aquatic surface hunter	Diurnal	Resident	900
34	*Plegadis falcinellus*	Pelecaniformes	Threskiornithidae	Piscivore	Semi-aquatic	Aquatic surface hunter	Diurnal	Resident	633.75
34	*Plegadis chihi*	Pelecaniformes	Threskiornithidae	Piscivore	Semi-aquatic	Aquatic surface hunter	Diurnal	Resident	612.5
34	*Platalea ajaja*	Pelecaniformes	Threskiornithidae	Piscivore	Semi-aquatic	Aquatic surface hunter	Diurnal	Resident	1475
34	*Laterallus ruber*	Gruiformes	Rallidae	Piscivore	Semi-aquatic	Aquatic surface hunter	Diurnal	Resident	45.2
34	*Laterallus exilis*	Gruiformes	Rallidae	Piscivore	Semi-aquatic	Aquatic surface hunter	Diurnal	Resident	31.5
34	*Laterallus jamaicensis*	Gruiformes	Rallidae	Piscivore	Semi-aquatic	Aquatic surface hunter	Diurnal	Resident	26.9
34	*Rallus longirostris*	Gruiformes	Rallidae	Piscivore	Semi-aquatic	Aquatic surface hunter	Diurnal	Resident	304.005
34	*Rallus obsoletus*	Gruiformes	Rallidae	Piscivore	Semi-aquatic	Aquatic surface hunter	Diurnal	Resident	253
34	*Rallus crepitans*	Gruiformes	Rallidae	Piscivore	Semi-aquatic	Aquatic surface hunter	Diurnal	Resident	300
34	*Rallus tenuirostris*	Gruiformes	Rallidae	Piscivore	Semi-aquatic	Aquatic surface hunter	Diurnal	Resident	275
34	*Aramides albiventris*	Gruiformes	Rallidae	Piscivore	Semi-aquatic	Aquatic surface hunter	Diurnal	Resident	392
34	*Aramides axillaris*	Gruiformes	Rallidae	Piscivore	Semi-aquatic	Aquatic surface hunter	Diurnal	Resident	275
34	*Amaurolimnas concolor*	Gruiformes	Rallidae	Piscivore	Semi-aquatic	Aquatic surface hunter	Diurnal	Resident	95
34	*Pardirallus maculatus*	Gruiformes	Rallidae	Piscivore	Semi-aquatic	Aquatic surface hunter	Diurnal	Resident	171
34	*Heliornis fulica*	Gruiformes	Heliornithidae	Piscivore	Semi-aquatic	Aquatic surface hunter	Diurnal	Resident	115
34	*Aramus guarauna*	Gruiformes	Aramidae	Piscivore	Semi-aquatic	Aquatic surface hunter	Diurnal	Resident	1160
34	*Jacana spinosa*	Charadriiformes	Jacanidae	Piscivore	Semi-aquatic	Aquatic surface hunter	Diurnal	Resident	108.5
34	*Cinclus mexicanus*	Passeriformes	Cinclidae	Piscivore	Semi-aquatic	Aquatic surface hunter	Diurnal	Resident	57.8
35	*Gavia immer*	Gaviiformes	Gaviidae	Piscivore	Semi-aquatic	Aquatic surface hunter	Diurnal	Winter migrant	5315.5
35	*Gavia adamsii*	Gaviiformes	Gaviidae	Piscivore	Semi-aquatic	Aquatic surface hunter	Diurnal	Winter migrant	4900
35	*Jabiru mycteria*	Ciconiiformes	Ciconiidae	Piscivore	Semi-aquatic	Aquatic surface hunter	Diurnal	Resident	8000
35	*Pelecanus erythrorhynchos*	Pelecaniformes	Pelecanidae	Piscivore	Semi-aquatic	Aquatic surface hunter	Diurnal	Winter migrant	5649.5
36	*Spatula clypeata*	Anseriformes	Anatidae	Piscivore	Semi-aquatic	Aquatic surface hunter	Cathemeral	Winter migrant	547.5
36	*Anas acuta*	Anseriformes	Anatidae	Piscivore	Semi-aquatic	Aquatic surface hunter	Cathemeral	Winter migrant	946.5
36	*Spatula querquedula*	Anseriformes	Anatidae	Piscivore	Semi-aquatic	Aquatic surface hunter	Cathemeral	Winter migrant	425
36	*Anas crecca*	Anseriformes	Anatidae	Piscivore	Semi-aquatic	Aquatic surface hunter	Cathemeral	Winter migrant	325
36	*Melanitta deglandi*	Anseriformes	Anatidae	Piscivore	Semi-aquatic	Aquatic surface hunter	Cathemeral	Winter migrant	1350
36	*Bucephala islandica*	Anseriformes	Anatidae	Piscivore	Semi-aquatic	Aquatic surface hunter	Cathemeral	Winter migrant	910
36	*Mergus merganser*	Anseriformes	Anatidae	Piscivore	Semi-aquatic	Aquatic surface hunter	Cathemeral	Winter migrant	1523
37	*Spatula discors*	Anseriformes	Anatidae	Piscivore	Semi-aquatic	Aquatic surface hunter	Diurnal	Winter migrant	360

37	*Aythya affinis*	Anseriformes	Anatidae	Piscivore	Semi-aquatic	Aquatic surface hunter	Diurnal	Winter migrant	819.5
37	*Melanitta perspicillata*	Anseriformes	Anatidae	Piscivore	Semi-aquatic	Aquatic surface hunter	Diurnal	Winter migrant	1022.3
37	*Melanitta americana*	Anseriformes	Anatidae	Piscivore	Semi-aquatic	Aquatic surface hunter	Diurnal	Winter migrant	1052.2
37	*Clangula hyemalis*	Anseriformes	Anatidae	Piscivore	Semi-aquatic	Aquatic surface hunter	Diurnal	Winter migrant	798.2
37	*Bucephala albeola*	Anseriformes	Anatidae	Piscivore	Semi-aquatic	Aquatic surface hunter	Diurnal	Winter migrant	401
37	*Bucephala clangula*	Anseriformes	Anatidae	Piscivore	Semi-aquatic	Aquatic surface hunter	Diurnal	Winter migrant	915
37	*Lophodytes cucullatus*	Anseriformes	Anatidae	Piscivore	Semi-aquatic	Aquatic surface hunter	Diurnal	Winter migrant	617
37	*Mergus serrator*	Anseriformes	Anatidae	Piscivore	Semi-aquatic	Aquatic surface hunter	Diurnal	Winter migrant	1041
37	*Gavia stellata*	Gaviiformes	Gaviidae	Piscivore	Semi-aquatic	Aquatic surface hunter	Diurnal	Winter migrant	1242.5
37	*Gavia arctica*	Gaviiformes	Gaviidae	Piscivore	Semi-aquatic	Aquatic surface hunter	Diurnal	Winter migrant	2600
37	*Gavia pacifica*	Gaviiformes	Gaviidae	Piscivore	Semi-aquatic	Aquatic surface hunter	Diurnal	Winter migrant	1668.5
37	*Podiceps auritus*	Podicipediformes	Podicipedidae	Piscivore	Semi-aquatic	Aquatic surface hunter	Diurnal	Winter migrant	453
37	*Podiceps grisegena*	Podicipediformes	Podicipedidae	Piscivore	Semi-aquatic	Aquatic surface hunter	Diurnal	Winter migrant	1023
37	*Podiceps nigricollis*	Podicipediformes	Podicipedidae	Piscivore	Semi-aquatic	Aquatic surface hunter	Diurnal	Winter migrant	368.85
37	*Botaurus lentiginosus*	Pelecaniformes	Ardeidae	Piscivore	Semi-aquatic	Aquatic surface hunter	Diurnal	Winter migrant	748.5
37	*Ardea herodias*	Pelecaniformes	Ardeidae	Piscivore	Semi-aquatic	Aquatic surface hunter	Diurnal	Winter migrant	2295
37	*Coturnicops noveboracensis*	Gruiformes	Rallidae	Piscivore	Semi-aquatic	Aquatic surface hunter	Diurnal	Winter migrant	55.7
37	*Rallus elegans*	Gruiformes	Rallidae	Piscivore	Semi-aquatic	Aquatic surface hunter	Diurnal	Winter migrant	360.5
37	*Rallus limicola*	Gruiformes	Rallidae	Piscivore	Semi-aquatic	Aquatic surface hunter	Diurnal	Winter migrant	79.35
38	*Ectopistes migratorius*	Columbiformes	Columbidae	Granivore	Terrestrial	Ground gleaner	Diurnal	Winter migrant	298.5
38	*Artemisiospiza nevadensis*	Passeriformes	Passerellidae	Granivore	Terrestrial	Ground gleaner	Diurnal	Winter migrant	18
38	*Calcarius lapponicus*	Passeriformes	Calcariidae	Granivore	Terrestrial	Ground gleaner	Diurnal	Winter migrant	27
38	*Calcarius ornatus*	Passeriformes	Calcariidae	Granivore	Terrestrial	Ground gleaner	Diurnal	Winter migrant	19.95
38	*Pipilo chlorurus*	Passeriformes	Emberizidae	Granivore	Terrestrial	Ground gleaner	Diurnal	Winter migrant	28.5
38	*Spizella pallida*	Passeriformes	Emberizidae	Granivore	Terrestrial	Ground gleaner	Diurnal	Winter migrant	12.1
38	*Spizella breweri*	Passeriformes	Emberizidae	Granivore	Terrestrial	Ground gleaner	Diurnal	Winter migrant	10.9
38	*Spizella pusilla*	Passeriformes	Emberizidae	Granivore	Terrestrial	Ground gleaner	Diurnal	Winter migrant	13.05
38	*Pooecetes gramineus*	Passeriformes	Emberizidae	Granivore	Terrestrial	Ground gleaner	Diurnal	Winter migrant	23.585
38	*Chondestes grammacus*	Passeriformes	Emberizidae	Granivore	Terrestrial	Ground gleaner	Diurnal	Winter migrant	30.4
38	*Artemisiospiza belli*	Passeriformes	Emberizidae	Granivore	Terrestrial	Ground gleaner	Diurnal	Winter migrant	15
38	*Passerculus sandwichensis*	Passeriformes	Emberizidae	Granivore	Terrestrial	Ground gleaner	Diurnal	Winter migrant	20.05
38	*Centronyx bairdii*	Passeriformes	Emberizidae	Granivore	Terrestrial	Ground gleaner	Diurnal	Winter migrant	17.65
38	*Ammospiza leconteii*	Passeriformes	Emberizidae	Granivore	Terrestrial	Ground gleaner	Diurnal	Winter migrant	13.6
38	*Ammospiza nelsoni*	Passeriformes	Emberizidae	Granivore	Terrestrial	Ground gleaner	Diurnal	Winter migrant	14.35
38	*Passerella iliaca*	Passeriformes	Emberizidae	Granivore	Terrestrial	Ground gleaner	Diurnal	Winter migrant	35.75
38	*Melospiza melodia*	Passeriformes	Emberizidae	Granivore	Terrestrial	Ground gleaner	Diurnal	Winter migrant	24.2
38	*Melospiza lincolnii*	Passeriformes	Emberizidae	Granivore	Terrestrial	Ground gleaner	Diurnal	Winter migrant	18.1
38	*Zonotrichia albicollis*	Passeriformes	Emberizidae	Granivore	Terrestrial	Ground gleaner	Diurnal	Winter migrant	25.9
38	*Zonotrichia querula*	Passeriformes	Emberizidae	Granivore	Terrestrial	Ground gleaner	Diurnal	Accidental	36.25
38	*Zonotrichia leucophrys*	Passeriformes	Emberizidae	Granivore	Terrestrial	Ground gleaner	Diurnal	Winter migrant	29.4
38	*Zonotrichia atricapilla*	Passeriformes	Emberizidae	Granivore	Terrestrial	Ground gleaner	Diurnal	Winter migrant	29.8
38	*Junco hyemalis*	Passeriformes	Emberizidae	Granivore	Terrestrial	Ground gleaner	Diurnal	Winter migrant	19.6
38	*Passerina cyanea*	Passeriformes	Cardinalidae	Granivore	Terrestrial	Ground gleaner	Diurnal	Winter migrant	14.5
38	*Passerina versicolor*	Passeriformes	Cardinalidae	Granivore	Terrestrial	Ground gleaner	Diurnal	Winter migrant	12.4
38	*Passerina ciris*	Passeriformes	Cardinalidae	Granivore	Terrestrial	Ground gleaner	Diurnal	Winter migrant	15.5
38	*Spiza americana*	Passeriformes	Cardinalidae	Granivore	Terrestrial	Ground gleaner	Diurnal	Winter migrant	26.85
38	*Dolichonyx oryzivorus*	Passeriformes	Icteridae	Granivore	Terrestrial	Ground gleaner	Diurnal	Winter migrant	45.8
38	*Agelaius phoeniceus*	Passeriformes	Icteridae	Granivore	Terrestrial	Ground gleaner	Diurnal	Winter migrant	52.55

38	*Xanthocephalus xanthocephalus*	Passeriformes	Icteridae	Granivore	Terrestrial	Ground gleaner	Diurnal	Winter migrant	64.5
38	*Molothrus ater*	Passeriformes	Icteridae	Granivore	Terrestrial	Ground gleaner	Diurnal	Winter migrant	43.9
38	*Haemorhous cassinii*	Passeriformes	Fringillidae	Granivore	Terrestrial	Ground gleaner	Diurnal	Winter migrant	26.95
39	*Dendrortyx barbatus*	Galliformes	Odontophoridae	Granivore	Terrestrial	Ground gleaner	Diurnal	Resident	432
39	*Dendrortyx macroura*	Galliformes	Odontophoridae	Granivore	Terrestrial	Ground gleaner	Diurnal	Resident	419.25
39	*Dendrortyx leucophrys*	Galliformes	Odontophoridae	Granivore	Terrestrial	Ground gleaner	Diurnal	Resident	350
39	*Oreortyx pictus*	Galliformes	Odontophoridae	Granivore	Terrestrial	Ground gleaner	Diurnal	Resident	239.2
39	*Callipepla squamata*	Galliformes	Odontophoridae	Granivore	Terrestrial	Ground gleaner	Diurnal	Resident	184
39	*Callipepla douglasii*	Galliformes	Odontophoridae	Granivore	Terrestrial	Ground gleaner	Diurnal	Resident	172
39	*Callipepla californica*	Galliformes	Odontophoridae	Granivore	Terrestrial	Ground gleaner	Diurnal	Resident	174.2
39	*Philortyx fasciatus*	Galliformes	Odontophoridae	Granivore	Terrestrial	Ground gleaner	Diurnal	Resident	130
39	*Colinus virginianus*	Galliformes	Odontophoridae	Granivore	Terrestrial	Ground gleaner	Diurnal	Resident	171.5
39	*Colinus nigrogularis*	Galliformes	Odontophoridae	Granivore	Terrestrial	Ground gleaner	Diurnal	Resident	129
39	*Odontophorus guttatus*	Galliformes	Odontophoridae	Granivore	Terrestrial	Ground gleaner	Diurnal	Resident	304
39	*Dactylortyx thoracicus*	Galliformes	Odontophoridae	Granivore	Terrestrial	Ground gleaner	Diurnal	Resident	205
39	*Cyrtonyx montezumae*	Galliformes	Odontophoridae	Granivore	Terrestrial	Ground gleaner	Diurnal	Resident	185.58
39	*Cyrtonyx ocellatus*	Galliformes	Odontophoridae	Granivore	Terrestrial	Ground gleaner	Diurnal	Resident	200
39	*Zenaida asiatica*	Columbiformes	Columbidae	Granivore	Terrestrial	Ground gleaner	Diurnal	Resident	153
39	*Zenaida aurita*	Columbiformes	Columbidae	Granivore	Terrestrial	Ground gleaner	Diurnal	Resident	159
39	*Zenaida macroura*	Columbiformes	Columbidae	Granivore	Terrestrial	Ground gleaner	Diurnal	Resident	119
39	*Zenaida graysoni*	Columbiformes	Columbidae	Granivore	Terrestrial	Ground gleaner	Diurnal	Resident	190
39	*Columbina inca*	Columbiformes	Columbidae	Granivore	Terrestrial	Ground gleaner	Diurnal	Resident	45.75
39	*Columbina passerina*	Columbiformes	Columbidae	Granivore	Terrestrial	Ground gleaner	Diurnal	Resident	34.4
39	*Columbina minuta*	Columbiformes	Columbidae	Granivore	Terrestrial	Ground gleaner	Diurnal	Resident	29.6
39	*Columbina talpacoti*	Columbiformes	Columbidae	Granivore	Terrestrial	Ground gleaner	Diurnal	Resident	47.25
39	*Claravis pretiosa*	Columbiformes	Columbidae	Granivore	Terrestrial	Ground gleaner	Diurnal	Resident	69.65
39	*Paraclaravis mondetoura*	Columbiformes	Columbidae	Granivore	Terrestrial	Ground gleaner	Diurnal	Resident	92.35
39	*Leptotila verreauxi*	Columbiformes	Columbidae	Granivore	Terrestrial	Ground gleaner	Diurnal	Resident	118.5
39	*Leptotila plumbeiceps*	Columbiformes	Columbidae	Granivore	Terrestrial	Ground gleaner	Diurnal	Resident	154.5
39	*Leptotila jamaicensis*	Columbiformes	Columbidae	Granivore	Terrestrial	Ground gleaner	Diurnal	Resident	156.75
39	*Leptotila cassini*	Columbiformes	Columbidae	Granivore	Terrestrial	Ground gleaner	Diurnal	Resident	157.25
39	*Zentrygon albifacies*	Columbiformes	Columbidae	Granivore	Terrestrial	Ground gleaner	Diurnal	Resident	305
39	*Zentrygon carrikeri*	Columbiformes	Columbidae	Granivore	Terrestrial	Ground gleaner	Diurnal	Resident	220
39	*Geotrygon montana*	Columbiformes	Columbidae	Granivore	Terrestrial	Ground gleaner	Diurnal	Resident	119.5
39	*Eremophila alpestris*	Passeriformes	Alaudidae	Granivore	Terrestrial	Ground gleaner	Diurnal	Resident	31.35
39	*Rhynchophanes mccownii*	Passeriformes	Calcariidae	Granivore	Terrestrial	Ground gleaner	Diurnal	Resident	25.7
39	*Volatinia jacarina*	Passeriformes	Emberizidae	Granivore	Terrestrial	Ground gleaner	Diurnal	Resident	9.7
39	*Sporophila torqueola*	Passeriformes	Emberizidae	Granivore	Terrestrial	Ground gleaner	Diurnal	Resident	7.8
39	*Sporophila minuta*	Passeriformes	Emberizidae	Granivore	Terrestrial	Ground gleaner	Diurnal	Resident	7.5
39	*Sporophila funerea*	Passeriformes	Emberizidae	Granivore	Terrestrial	Ground gleaner	Diurnal	Resident	12.3
39	*Tiaris olivaceus*	Passeriformes	Emberizidae	Granivore	Terrestrial	Ground gleaner	Diurnal	Resident	8.9
39	*Haplospiza rustica*	Passeriformes	Emberizidae	Granivore	Terrestrial	Ground gleaner	Diurnal	Resident	15.6
39	*Sicalis luteola*	Passeriformes	Emberizidae	Granivore	Terrestrial	Ground gleaner	Diurnal	Resident	16
39	*Arremon aurantiirostris*	Passeriformes	Emberizidae	Granivore	Terrestrial	Ground gleaner	Diurnal	Resident	34.5
39	*Arremon brunneinucha*	Passeriformes	Emberizidae	Granivore	Terrestrial	Ground gleaner	Diurnal	Resident	46.6
39	*Arremon virenticeps*	Passeriformes	Emberizidae	Granivore	Terrestrial	Ground gleaner	Diurnal	Resident	41
39	*Arremonops rufivirgatus*	Passeriformes	Emberizidae	Granivore	Terrestrial	Ground gleaner	Diurnal	Resident	22.44
39	*Arremonops chloronotus*	Passeriformes	Emberizidae	Granivore	Terrestrial	Ground gleaner	Diurnal	Resident	27.3

39	*Atlapetes albinucha*	Passeriformes	Emberizidae	Granivore	Terrestrial	Ground gleaner	Diurnal	Resident	32.4
39	*Atlapetes pileatus*	Passeriformes	Emberizidae	Granivore	Terrestrial	Ground gleaner	Diurnal	Resident	24
39	*Pipilo ocai*	Passeriformes	Emberizidae	Granivore	Terrestrial	Ground gleaner	Diurnal	Resident	61.05
39	*Pipilo maculatus*	Passeriformes	Emberizidae	Granivore	Terrestrial	Ground gleaner	Diurnal	Resident	39.38
39	*Pipilo erythrophthalmus*	Passeriformes	Emberizidae	Granivore	Terrestrial	Ground gleaner	Diurnal	Resident	40.5
39	*Aimophila rufescens*	Passeriformes	Emberizidae	Granivore	Terrestrial	Ground gleaner	Diurnal	Resident	34.3
39	*Aimophila ruficeps*	Passeriformes	Emberizidae	Granivore	Terrestrial	Ground gleaner	Diurnal	Resident	18.8
39	*Aimophila notosticta*	Passeriformes	Emberizidae	Granivore	Terrestrial	Ground gleaner	Diurnal	Resident	27.1
39	*Melozone biarcuata*	Passeriformes	Emberizidae	Granivore	Terrestrial	Ground gleaner	Diurnal	Resident	28
39	*Melozone kieneri*	Passeriformes	Emberizidae	Granivore	Terrestrial	Ground gleaner	Diurnal	Resident	38.7
39	*Melozone fusca*	Passeriformes	Emberizidae	Granivore	Terrestrial	Ground gleaner	Diurnal	Resident	45.55
39	*Melozone albicollis*	Passeriformes	Emberizidae	Granivore	Terrestrial	Ground gleaner	Diurnal	Resident	46.5
39	*Melozone crissalis*	Passeriformes	Emberizidae	Granivore	Terrestrial	Ground gleaner	Diurnal	Resident	52.85
39	*Peucaea sumichrasti*	Passeriformes	Emberizidae	Granivore	Terrestrial	Ground gleaner	Diurnal	Resident	29.45
39	*Peucaea carpalis*	Passeriformes	Emberizidae	Granivore	Terrestrial	Ground gleaner	Diurnal	Resident	15.08
39	*Peucaea ruficauda*	Passeriformes	Emberizidae	Granivore	Terrestrial	Ground gleaner	Diurnal	Resident	28.2
39	*Peucaea humeralis*	Passeriformes	Emberizidae	Granivore	Terrestrial	Ground gleaner	Diurnal	Resident	24.1
39	*Peucaea mystacalis*	Passeriformes	Emberizidae	Granivore	Terrestrial	Ground gleaner	Diurnal	Resident	22.2
39	*Oriturus superciliosus*	Passeriformes	Emberizidae	Granivore	Terrestrial	Ground gleaner	Diurnal	Resident	41.5
39	*Spizella passerina*	Passeriformes	Emberizidae	Granivore	Terrestrial	Ground gleaner	Diurnal	Resident	13.25
39	*Spizella wortheni*	Passeriformes	Emberizidae	Granivore	Terrestrial	Ground gleaner	Diurnal	Resident	12.4
39	*Spizella atrogularis*	Passeriformes	Emberizidae	Granivore	Terrestrial	Ground gleaner	Diurnal	Resident	12.85
39	*Xenospiza baileyi*	Passeriformes	Emberizidae	Granivore	Terrestrial	Ground gleaner	Diurnal	Resident	17.4
39	*Zonotrichia capensis*	Passeriformes	Emberizidae	Granivore	Terrestrial	Ground gleaner	Diurnal	Resident	20.5
39	*Junco phaeonotus*	Passeriformes	Emberizidae	Granivore	Terrestrial	Ground gleaner	Diurnal	Resident	20.4
39	*Junco insularis*	Passeriformes	Emberizidae	Granivore	Terrestrial	Ground gleaner	Diurnal	Resident	19.6
39	*Junco bairdi*	Passeriformes	Emberizidae	Granivore	Terrestrial	Ground gleaner	Diurnal	Resident	19.6
39	*Cyanoloxia cyanoides*	Passeriformes	Cardinalidae	Granivore	Terrestrial	Ground gleaner	Diurnal	Resident	32.5
39	*Cyanocompsa parellina*	Passeriformes	Cardinalidae	Granivore	Terrestrial	Ground gleaner	Diurnal	Resident	15
39	*Passerina rositae*	Passeriformes	Cardinalidae	Granivore	Terrestrial	Ground gleaner	Diurnal	Resident	20
39	*Passerina leclancherii*	Passeriformes	Cardinalidae	Granivore	Terrestrial	Ground gleaner	Diurnal	Resident	14
39	*Molothrus aeneus*	Passeriformes	Icteridae	Granivore	Terrestrial	Ground gleaner	Diurnal	Resident	62.9
39	*Molothrus oryzivorus*	Passeriformes	Icteridae	Granivore	Terrestrial	Ground gleaner	Diurnal	Resident	190.5
39	*Haemorhous mexicanus*	Passeriformes	Fringillidae	Granivore	Terrestrial	Ground gleaner	Diurnal	Resident	21.4
40	*Dendrocygna autumnalis*	Anseriformes	Anatidae	Granivore	Semi-aquatic	Ground gleaner	Diurnal	Resident	778.2
40	*Dendrocygna bicolor*	Anseriformes	Anatidae	Granivore	Semi-aquatic	Aquatic surface gleaner	Diurnal	Resident	756.9
41	*Patagioenas fasciata*	Columbiformes	Columbidae	Granivore	Semi-arboreal	Ground gleaner	Diurnal	Resident	342.5
41	*Nucifraga columbiana*	Passeriformes	Corvidae	Granivore	Semi-arboreal	Arboreal gleaner	Diurnal	Resident	172.5
41	*Cardinalis cardinalis*	Passeriformes	Cardinalidae	Granivore	Semi-arboreal	Ground gleaner	Diurnal	Resident	45.25
41	*Cardinalis sinuatus*	Passeriformes	Cardinalidae	Granivore	Semi-arboreal	Ground gleaner	Diurnal	Resident	35.5
41	*Coccothraustes abeillei*	Passeriformes	Fringillidae	Granivore	Semi-arboreal	Ground gleaner	Diurnal	Resident	48.5
42	*Geococcyx velox*	Cuculiformes	Cuculidae	Omnivore	Terrestrial	Ground gleaner	Diurnal	Resident	203
42	*Geococcyx californianus*	Cuculiformes	Cuculidae	Omnivore	Terrestrial	Ground gleaner	Diurnal	Resident	302.71
42	*Gymnorhinus cyanocephalus*	Passeriformes	Corvidae	Omnivore	Terrestrial	Ground gleaner	Diurnal	Resident	105
42	*Aphelocoma woodhouseii*	Passeriformes	Corvidae	Omnivore	Terrestrial	Ground gleaner	Diurnal	Resident	120
42	*Aphelocoma californica*	Passeriformes	Corvidae	Omnivore	Terrestrial	Ground gleaner	Diurnal	Resident	97.55
42	*Aphelocoma ultramarina*	Passeriformes	Corvidae	Omnivore	Terrestrial	Ground gleaner	Diurnal	Resident	124
42	*Aphelocoma wollweberi*	Passeriformes	Corvidae	Omnivore	Terrestrial	Ground gleaner	Diurnal	Resident	124.45

42	*Aphelocoma unicolor*	Passeriformes	Corvidae	Omnivore	Terrestrial	Ground gleaner	Diurnal	Resident	124
42	*Corvus brachyrhynchos*	Passeriformes	Corvidae	Omnivore	Terrestrial	Ground gleaner	Diurnal	Winter migrant	448
42	*Corvus imparatus*	Passeriformes	Corvidae	Omnivore	Terrestrial	Ground gleaner	Diurnal	Resident	221
42	*Corvus sinaloae*	Passeriformes	Corvidae	Omnivore	Terrestrial	Ground gleaner	Diurnal	Resident	244
42	*Corvus cryptoleucus*	Passeriformes	Corvidae	Omnivore	Terrestrial	Ground gleaner	Diurnal	Winter migrant	534
42	*Corvus corax*	Passeriformes	Corvidae	Omnivore	Terrestrial	Ground gleaner	Diurnal	Resident	782.5
42	*Quiscalus mexicanus*	Passeriformes	Icteridae	Omnivore	Terrestrial	Ground gleaner	Diurnal	Resident	168.7
42	*Quiscalus palustris*	Passeriformes	Icteridae	Omnivore	Terrestrial	Ground gleaner	Diurnal	Resident	143
42	*Quiscalus quiscula*	Passeriformes	Icteridae	Omnivore	Terrestrial	Ground gleaner	Diurnal	Winter migrant	110
43	*Coragyps atratus*	Accipitriformes	Cathartidae	Scavenger	Terrestrial	Scavenger	Diurnal	Resident	2080.5
43	*Cathartes aura*	Accipitriformes	Cathartidae	Scavenger	Terrestrial	Scavenger	Diurnal	Resident	2033
43	*Cathartes burrovianus*	Accipitriformes	Cathartidae	Scavenger	Terrestrial	Scavenger	Diurnal	Resident	976.5
43	*Gymnogyps californianus*	Accipitriformes	Cathartidae	Scavenger	Terrestrial	Scavenger	Diurnal	Resident	9102
43	*Sarcoramphus papa*	Accipitriformes	Cathartidae	Scavenger	Terrestrial	Scavenger	Diurnal	Resident	3400
43	*Caracara plancus*	Falconiformes	Falconidae	Scavenger	Terrestrial	Scavenger	Diurnal	Resident	1168.35
44	*Coereba flaveola*	Passeriformes	Incertae	Nectarivore	Arboreal	Arboreal gleaner	Diurnal	Resident	9.4
44	*Cyanerpes lucidus*	Passeriformes	Thraupidae	Nectarivore	Arboreal	Arboreal gleaner	Diurnal	Resident	11.4
44	*Cyanerpes cyaneus*	Passeriformes	Thraupidae	Nectarivore	Arboreal	Arboreal gleaner	Diurnal	Resident	14
44	*Diglossa baritula*	Passeriformes	Emberizidae	Nectarivore	Arboreal	Arboreal gleaner	Diurnal	Resident	8
45	*Calothorax lucifer*	Apodiformes	Trochilidae	Nectarivore	Volant	Arboreal gleaner	Diurnal	Winter migrant	3.35
45	*Archilochus colubris*	Apodiformes	Trochilidae	Nectarivore	Volant	Arboreal gleaner	Diurnal	Winter migrant	3.125
45	*Archilochus alexandri*	Apodiformes	Trochilidae	Nectarivore	Volant	Arboreal gleaner	Diurnal	Winter migrant	3.4
45	*Calypte anna*	Apodiformes	Trochilidae	Nectarivore	Volant	Arboreal gleaner	Diurnal	Winter migrant	4.25
45	*Calypte costae*	Apodiformes	Trochilidae	Nectarivore	Volant	Arboreal gleaner	Diurnal	Winter migrant	3.1
45	*Selasphorus sasin*	Apodiformes	Trochilidae	Nectarivore	Volant	Arboreal gleaner	Diurnal	Winter migrant	3.6
45	*Selasphorus calliope*	Apodiformes	Trochilidae	Nectarivore	Volant	Arboreal gleaner	Diurnal	Winter migrant	2.65
45	*Amazilia yucatanensis*	Apodiformes	Trochilidae	Nectarivore	Volant	Arboreal gleaner	Diurnal	Winter migrant	3.85
46	*Florisuga mellivora*	Apodiformes	Trochilidae	Nectarivore	Volant	Arboreal gleaner	Diurnal	Resident	7.4
46	*Phaethornis longirostris*	Apodiformes	Trochilidae	Nectarivore	Volant	Arboreal gleaner	Diurnal	Resident	3
46	*Phaethornis striigularis*	Apodiformes	Trochilidae	Nectarivore	Volant	Arboreal gleaner	Diurnal	Resident	3
46	*Phaethornis mexicanus*	Apodiformes	Trochilidae	Nectarivore	Volant	Arboreal gleaner	Diurnal	Resident	3
46	*Colibri thalassinus*	Apodiformes	Trochilidae	Nectarivore	Volant	Arboreal gleaner	Diurnal	Resident	5.9
46	*Heliothryx barroti*	Apodiformes	Trochilidae	Nectarivore	Volant	Arboreal gleaner	Diurnal	Resident	5.5
46	*Anthracothorax prevostii*	Apodiformes	Trochilidae	Nectarivore	Volant	Arboreal gleaner	Diurnal	Resident	6.95
46	*Lophornis brachylophus*	Apodiformes	Trochilidae	Nectarivore	Volant	Arboreal gleaner	Diurnal	Resident	2.6
46	*Lophornis helenae*	Apodiformes	Trochilidae	Nectarivore	Volant	Arboreal gleaner	Diurnal	Resident	2.6
46	*Eugenes fulgens*	Apodiformes	Trochilidae	Nectarivore	Volant	Arboreal gleaner	Diurnal	Resident	7.05
46	*Heliomaster longirostris*	Apodiformes	Trochilidae	Nectarivore	Volant	Arboreal gleaner	Diurnal	Resident	6.6
46	*Heliomaster constantii*	Apodiformes	Trochilidae	Nectarivore	Volant	Arboreal gleaner	Diurnal	Resident	7.3
46	*Lampornis viridipallens*	Apodiformes	Trochilidae	Nectarivore	Volant	Arboreal gleaner	Diurnal	Resident	5.35
46	*Lampornis amethystinus*	Apodiformes	Trochilidae	Nectarivore	Volant	Arboreal gleaner	Diurnal	Resident	5.2
46	*Lampornis clemenciae*	Apodiformes	Trochilidae	Nectarivore	Volant	Arboreal gleaner	Diurnal	Resident	7.6
46	*Lamprolaima rhami*	Apodiformes	Trochilidae	Nectarivore	Volant	Arboreal gleaner	Diurnal	Resident	6.1
46	*Doricha enicura*	Apodiformes	Trochilidae	Nectarivore	Volant	Arboreal gleaner	Diurnal	Resident	2.4
46	*Doricha eliza*	Apodiformes	Trochilidae	Nectarivore	Volant	Arboreal gleaner	Diurnal	Resident	2.45
46	*Tilmatura dupontii*	Apodiformes	Trochilidae	Nectarivore	Volant	Arboreal gleaner	Diurnal	Resident	2.2
46	*Calothorax pulcher*	Apodiformes	Trochilidae	Nectarivore	Volant	Arboreal gleaner	Diurnal	Resident	2.9
46	*Selasphorus heloisa*	Apodiformes	Trochilidae	Nectarivore	Volant	Arboreal gleaner	Diurnal	Resident	2.2

46	*Selasphorus ellioti*	Apodiformes	Trochilidae	Nectarivore	Volant	Arboreal gleaner	Diurnal	Resident	2.3
46	*Selasphorus platycercus*	Apodiformes	Trochilidae	Nectarivore	Volant	Arboreal gleaner	Diurnal	Resident	3.55
46	*Selasphorus rufus*	Apodiformes	Trochilidae	Nectarivore	Volant	Arboreal gleaner	Diurnal	Resident	3.46
46	*Cynanthus auriceps*	Apodiformes	Trochilidae	Nectarivore	Volant	Arboreal gleaner	Diurnal	Resident	2.2
46	*Cynanthus canivetii*	Apodiformes	Trochilidae	Nectarivore	Volant	Arboreal gleaner	Diurnal	Resident	2.6
46	*Cynanthus forficatus*	Apodiformes	Trochilidae	Nectarivore	Volant	Arboreal gleaner	Diurnal	Resident	2.4
46	*Phaeoptila sordida*	Apodiformes	Trochilidae	Nectarivore	Volant	Arboreal gleaner	Diurnal	Resident	4.4
46	*Cynanthus latirostris*	Apodiformes	Trochilidae	Nectarivore	Volant	Arboreal gleaner	Diurnal	Resident	3.3
46	*Abeillia abeillei*	Apodiformes	Trochilidae	Nectarivore	Volant	Arboreal gleaner	Diurnal	Resident	2.7
46	*Phaeochroa cuvierii*	Apodiformes	Trochilidae	Nectarivore	Volant	Arboreal gleaner	Diurnal	Resident	8.9
46	*Pampa curvipennis*	Apodiformes	Trochilidae	Nectarivore	Volant	Arboreal gleaner	Diurnal	Resident	6.2
46	*Pampa excellens*	Apodiformes	Trochilidae	Nectarivore	Volant	Arboreal gleaner	Diurnal	Resident	9.25
46	*Pampa rufa*	Apodiformes	Trochilidae	Nectarivore	Volant	Arboreal gleaner	Diurnal	Resident	7.85
46	*Campylopterus hemileucurus*	Apodiformes	Trochilidae	Nectarivore	Volant	Arboreal gleaner	Diurnal	Resident	11.5
46	*Eupherusa eximia*	Apodiformes	Trochilidae	Nectarivore	Volant	Arboreal gleaner	Diurnal	Resident	4.3
46	*Eupherusa cyanophrys*	Apodiformes	Trochilidae	Nectarivore	Volant	Arboreal gleaner	Diurnal	Resident	4.9
46	*Eupherusa poliocerca*	Apodiformes	Trochilidae	Nectarivore	Volant	Arboreal gleaner	Diurnal	Resident	4.6
46	*Eupherusa ridgwayi*	Apodiformes	Trochilidae	Nectarivore	Volant	Arboreal gleaner	Diurnal	Resident	3.8
46	*Chlorestes candida*	Apodiformes	Trochilidae	Nectarivore	Volant	Arboreal gleaner	Diurnal	Resident	3.8
46	*Saucerottia cyanocephala*	Apodiformes	Trochilidae	Nectarivore	Volant	Arboreal gleaner	Diurnal	Resident	5.8
46	*Saucerottia beryllina*	Apodiformes	Trochilidae	Nectarivore	Volant	Arboreal gleaner	Diurnal	Resident	4.65
46	*Saucerottia cyanura*	Apodiformes	Trochilidae	Nectarivore	Volant	Arboreal gleaner	Diurnal	Resident	3.9
46	*Amazilia tzacatl*	Apodiformes	Trochilidae	Nectarivore	Volant	Arboreal gleaner	Diurnal	Resident	5
46	*Amazilia rutila*	Apodiformes	Trochilidae	Nectarivore	Volant	Arboreal gleaner	Diurnal	Resident	5
46	*Ramosomyia violiceps*	Apodiformes	Trochilidae	Nectarivore	Volant	Arboreal gleaner	Diurnal	Resident	5.4
46	*Ramosomyia viridifrons*	Apodiformes	Trochilidae	Nectarivore	Volant	Arboreal gleaner	Diurnal	Resident	5.4
46	*Chlorestes eliciae*	Apodiformes	Trochilidae	Nectarivore	Volant	Arboreal gleaner	Diurnal	Resident	3.6
46	*Basilinna leucotis*	Apodiformes	Trochilidae	Nectarivore	Volant	Arboreal gleaner	Diurnal	Resident	3.4
46	*Basilinna xantusii*	Apodiformes	Trochilidae	Nectarivore	Volant	Arboreal gleaner	Diurnal	Resident	3.475
47	*Penelope purpurascens*	Galliformes	Cracidae	Frugivore	Arboreal	Arboreal gleaner	Diurnal	Resident	2060
47	*Oreophasis derbianus*	Galliformes	Cracidae	Frugivore	Arboreal	Arboreal gleaner	Diurnal	Resident	2076
47	*Patagioenas cayennensis*	Columbiformes	Columbidae	Frugivore	Arboreal	Arboreal gleaner	Diurnal	Resident	240
47	*Trogon massena*	Trogoniformes	Trogonidae	Frugivore	Arboreal	Arboreal gleaner	Diurnal	Resident	141
47	*Trogon melanocephalus*	Trogoniformes	Trogonidae	Frugivore	Arboreal	Arboreal gleaner	Diurnal	Resident	85.1
47	*Trogon citreolus*	Trogoniformes	Trogonidae	Frugivore	Arboreal	Arboreal gleaner	Diurnal	Resident	83.1
47	*Trogon caligatus*	Trogoniformes	Trogonidae	Frugivore	Arboreal	Arboreal gleaner	Diurnal	Resident	51.5
47	*Trogon elegans*	Trogoniformes	Trogonidae	Frugivore	Arboreal	Arboreal gleaner	Diurnal	Resident	67.3
47	*Trogon mexicanus*	Trogoniformes	Trogonidae	Frugivore	Arboreal	Arboreal gleaner	Diurnal	Resident	69.3
47	*Trogon collaris*	Trogoniformes	Trogonidae	Frugivore	Arboreal	Arboreal gleaner	Diurnal	Resident	64.4
47	*Euptilotis neoxenus*	Trogoniformes	Trogonidae	Frugivore	Arboreal	Arboreal gleaner	Diurnal	Resident	122.5
47	*Pharomachrus mocinno*	Trogoniformes	Trogonidae	Frugivore	Arboreal	Arboreal gleaner	Diurnal	Resident	206
47	*Aulacorhynchus prasinus*	Piciformes	Ramphastidae	Frugivore	Arboreal	Arboreal gleaner	Diurnal	Resident	154.5
47	*Pteroglossus torquatus*	Piciformes	Ramphastidae	Frugivore	Arboreal	Arboreal gleaner	Diurnal	Resident	226
47	*Ramphastos sulfuratus*	Piciformes	Ramphastidae	Frugivore	Arboreal	Arboreal gleaner	Diurnal	Resident	339
47	*Psittacara holochlorus*	Psittaciformes	Psittacidae	Frugivore	Arboreal	Arboreal gleaner	Diurnal	Resident	138
47	*Psittacara strenuus*	Psittaciformes	Psittacidae	Frugivore	Arboreal	Arboreal gleaner	Diurnal	Resident	208
47	*Eupsittula nana*	Psittaciformes	Psittacidae	Frugivore	Arboreal	Arboreal gleaner	Diurnal	Resident	80.95
47	*Eupsittula canicularis*	Psittaciformes	Psittacidae	Frugivore	Arboreal	Arboreal gleaner	Diurnal	Resident	75.2

47	*Ara militaris*	Psittaciformes	Psittacidae	Frugivore	Arboreal	Arboreal gleaner	Diurnal	Resident	1134
47	*Ara macao*	Psittaciformes	Psittacidae	Frugivore	Arboreal	Arboreal gleaner	Diurnal	Resident	957.5
47	*Forpus cyanopygius*	Psittaciformes	Psittacidae	Frugivore	Arboreal	Arboreal gleaner	Diurnal	Resident	33.5
47	*Brotogeris jugularis*	Psittaciformes	Psittacidae	Frugivore	Arboreal	Arboreal gleaner	Diurnal	Resident	63.5
47	*Pyrilia haematotis*	Psittaciformes	Psittacidae	Frugivore	Arboreal	Arboreal gleaner	Diurnal	Resident	149
47	*Pionus senilis*	Psittaciformes	Psittacidae	Frugivore	Arboreal	Arboreal gleaner	Diurnal	Resident	212
47	*Amazona albifrons*	Psittaciformes	Psittacidae	Frugivore	Arboreal	Arboreal gleaner	Diurnal	Resident	218
47	*Amazona xantholora*	Psittaciformes	Psittacidae	Frugivore	Arboreal	Arboreal gleaner	Diurnal	Resident	217
47	*Amazona finschi*	Psittaciformes	Psittacidae	Frugivore	Arboreal	Arboreal gleaner	Diurnal	Resident	302
47	*Amazona autumnalis*	Psittaciformes	Psittacidae	Frugivore	Arboreal	Arboreal gleaner	Diurnal	Resident	416
47	*Amazona farinosa*	Psittaciformes	Psittacidae	Frugivore	Arboreal	Arboreal gleaner	Diurnal	Resident	610
47	*Cotinga amabilis*	Passeriformes	Cotingidae	Frugivore	Arboreal	Arboreal gleaner	Diurnal	Resident	71.5
47	*Manacus candei*	Passeriformes	Pipridae	Frugivore	Arboreal	Arboreal gleaner	Diurnal	Resident	19.85
47	*Chiroxiphia linearis*	Passeriformes	Pipridae	Frugivore	Arboreal	Arboreal gleaner	Diurnal	Resident	17.95
47	*Ceratopipra mentalis*	Passeriformes	Pipridae	Frugivore	Arboreal	Arboreal gleaner	Diurnal	Resident	15
47	*Spindalis zena*	Passeriformes	Spindalidae	Frugivore	Arboreal	Arboreal gleaner	Diurnal	Resident	28.5
47	*Bombycilla cedrorum*	Passeriformes	Bombycillidae	Frugivore	Arboreal	Arboreal gleaner	Diurnal	Winter migrant	34.75
47	*Ramphocelus passerinii*	Passeriformes	Thraupidae	Frugivore	Arboreal	Arboreal gleaner	Diurnal	Resident	32
47	*Thraupis episcopus*	Passeriformes	Thraupidae	Frugivore	Arboreal	Arboreal gleaner	Diurnal	Resident	35
47	*Thraupis abbas*	Passeriformes	Thraupidae	Frugivore	Arboreal	Arboreal gleaner	Diurnal	Resident	45
47	*Poecilostreptus cabanisi*	Passeriformes	Thraupidae	Frugivore	Arboreal	Arboreal gleaner	Diurnal	Resident	20
47	*Stilpnia larvata*	Passeriformes	Thraupidae	Frugivore	Arboreal	Arboreal gleaner	Diurnal	Resident	20
47	*Chlorophanes spiza*	Passeriformes	Thraupidae	Frugivore	Arboreal	Arboreal gleaner	Diurnal	Resident	19
47	*Psarocolius wagleri*	Passeriformes	Icteridae	Frugivore	Arboreal	Arboreal gleaner	Diurnal	Resident	163.5
47	*Psarocolius montezuma*	Passeriformes	Icteridae	Frugivore	Arboreal	Arboreal gleaner	Diurnal	Resident	324
47	*Chlorophonia occipitalis*	Passeriformes	Fringillidae	Frugivore	Arboreal	Arboreal gleaner	Diurnal	Resident	25.8
48	*Ortalis vetula*	Galliformes	Cracidae	Frugivore	Semi-arboreal	Arboreal gleaner	Diurnal	Resident	563
48	*Ortalis wagleri*	Galliformes	Cracidae	Frugivore	Semi-arboreal	Arboreal gleaner	Diurnal	Resident	834
48	*Ortalis poliocephala*	Galliformes	Cracidae	Frugivore	Semi-arboreal	Arboreal gleaner	Diurnal	Resident	760
48	*Ortalis leucogastra*	Galliformes	Cracidae	Frugivore	Semi-arboreal	Arboreal gleaner	Diurnal	Resident	499.5
48	*Penelopina nigra*	Galliformes	Cracidae	Frugivore	Semi-arboreal	Arboreal gleaner	Diurnal	Resident	890
48	*Patagioenas speciosa*	Columbiformes	Columbidae	Frugivore	Semi-arboreal	Arboreal gleaner	Diurnal	Resident	243.5
48	*Patagioenas flavirostris*	Columbiformes	Columbidae	Frugivore	Semi-arboreal	Arboreal gleaner	Diurnal	Resident	324
48	*Patagioenas nigrirostris*	Columbiformes	Columbidae	Frugivore	Semi-arboreal	Arboreal gleaner	Diurnal	Resident	176
48	*Saltator grandis*	Passeriformes	Incertae	Frugivore	Semi-arboreal	Arboreal gleaner	Diurnal	Resident	54.9
48	*Saltator maximus*	Passeriformes	Incertae	Frugivore	Semi-arboreal	Arboreal gleaner	Diurnal	Resident	47.65
48	*Saltator atriceps*	Passeriformes	Incertae	Frugivore	Semi-arboreal	Arboreal gleaner	Diurnal	Resident	79.7
48	*Euphonia affinis*	Passeriformes	Fringillidae	Frugivore	Semi-arboreal	Arboreal gleaner	Diurnal	Resident	10
48	*Euphonia hirundinacea*	Passeriformes	Fringillidae	Frugivore	Semi-arboreal	Arboreal gleaner	Diurnal	Resident	14
48	*Chlorophonia elegantissima*	Passeriformes	Fringillidae	Frugivore	Semi-arboreal	Arboreal gleaner	Diurnal	Resident	15
48	*Euphonia gouldi*	Passeriformes	Fringillidae	Frugivore	Semi-arboreal	Arboreal gleaner	Diurnal	Resident	14
48	*Euphonia minuta*	Passeriformes	Fringillidae	Frugivore	Semi-arboreal	Arboreal gleaner	Diurnal	Resident	10
49	*Tinamus major*	Tinamiformes	Tinamidae	Frugivore	Terrestrial	Ground gleaner	Diurnal	Resident	1052
49	*Crypturellus soui*	Tinamiformes	Tinamidae	Frugivore	Terrestrial	Ground gleaner	Diurnal	Resident	209
49	*Crypturellus cinnamomeus*	Tinamiformes	Tinamidae	Frugivore	Terrestrial	Ground gleaner	Diurnal	Resident	422
49	*Crypturellus boucardi*	Tinamiformes	Tinamidae	Frugivore	Terrestrial	Ground gleaner	Diurnal	Resident	443
50	*Crax rubra*	Galliformes	Cracidae	Frugivore	Semi-arboreal	Ground gleaner	Diurnal	Resident	3909
50	*Patagioenas leucocephala*	Columbiformes	Columbidae	Frugivore	Semi-arboreal	Ground gleaner	Diurnal	Resident	247

51	*Cyanolyca mirabilis*	Passeriformes	Corvidae	Omnivore	Arboreal	Arboreal gleaner	Diurnal	Resident	52.4
51	*Cyanolyca pumilo*	Passeriformes	Corvidae	Omnivore	Arboreal	Arboreal gleaner	Diurnal	Resident	47
51	*Cyanolyca cucullata*	Passeriformes	Corvidae	Omnivore	Arboreal	Arboreal gleaner	Diurnal	Resident	101.6
51	*Calocitta colliei*	Passeriformes	Corvidae	Omnivore	Arboreal	Arboreal gleaner	Diurnal	Resident	234
51	*Calocitta formosa*	Passeriformes	Corvidae	Omnivore	Arboreal	Arboreal gleaner	Diurnal	Resident	210
51	*Psilorhinus morio*	Passeriformes	Corvidae	Omnivore	Arboreal	Arboreal gleaner	Diurnal	Resident	204
51	*Cyanocorax dickeyi*	Passeriformes	Corvidae	Omnivore	Arboreal	Arboreal gleaner	Diurnal	Resident	176
51	*Cyanocorax yncas*	Passeriformes	Corvidae	Omnivore	Arboreal	Arboreal gleaner	Diurnal	Resident	89.35
51	*Cyanocorax sanblasianus*	Passeriformes	Corvidae	Omnivore	Arboreal	Arboreal gleaner	Diurnal	Resident	109
51	*Cyanocorax yucatanicus*	Passeriformes	Corvidae	Omnivore	Arboreal	Arboreal gleaner	Diurnal	Resident	118
51	*Cyanocorax beecheii*	Passeriformes	Corvidae	Omnivore	Arboreal	Arboreal gleaner	Diurnal	Resident	193
51	*Cyanocitta stelleri*	Passeriformes	Corvidae	Omnivore	Semi-arboreal	Arboreal gleaner	Diurnal	Resident	106
52	*Rhynchopsitta pachyrhyncha*	Psittaciformes	Psittacidae	Granivore	Arboreal	Arboreal gleaner	Diurnal	Resident	325
52	*Rhynchopsitta terrisi*	Psittaciformes	Psittacidae	Granivore	Arboreal	Arboreal gleaner	Diurnal	Resident	442
52	*Bolborhynchus lineola*	Psittaciformes	Psittacidae	Granivore	Arboreal	Arboreal gleaner	Diurnal	Resident	54.3
52	*Amazona viridigenalis*	Psittaciformes	Psittacidae	Granivore	Arboreal	Arboreal gleaner	Diurnal	Resident	300
52	*Amazona oratrix*	Psittaciformes	Psittacidae	Granivore	Arboreal	Arboreal gleaner	Diurnal	Resident	517
52	*Amazona auropalliata*	Psittaciformes	Psittacidae	Granivore	Arboreal	Arboreal gleaner	Diurnal	Resident	450
52	*Rhodothraupis celaeno*	Passeriformes	Cardinalidae	Granivore	Arboreal	Arboreal gleaner	Diurnal	Resident	60
52	*Pheucticus chrysopeplus*	Passeriformes	Cardinalidae	Granivore	Arboreal	Arboreal gleaner	Diurnal	Resident	62.7
52	*Pheucticus melanocephalus*	Passeriformes	Cardinalidae	Granivore	Arboreal	Arboreal gleaner	Diurnal	Winter migrant	46.25
52	*Amaurospiza concolor*	Passeriformes	Cardinalidae	Granivore	Arboreal	Arboreal gleaner	Diurnal	Resident	13
52	*Haemorhous purpureus*	Passeriformes	Fringillidae	Granivore	Arboreal	Arboreal gleaner	Diurnal	Winter migrant	25.175
52	*Loxia curvirostra*	Passeriformes	Fringillidae	Granivore	Arboreal	Arboreal gleaner	Diurnal	Resident	29.79
52	*Spinus pinus*	Passeriformes	Fringillidae	Granivore	Arboreal	Arboreal gleaner	Diurnal	Resident	14.605
52	*Spinus atriceps*	Passeriformes	Fringillidae	Granivore	Arboreal	Arboreal gleaner	Diurnal	Resident	13.5
52	*Spinus notatus*	Passeriformes	Fringillidae	Granivore	Arboreal	Arboreal gleaner	Diurnal	Resident	10.9
52	*Spinus psaltria*	Passeriformes	Fringillidae	Granivore	Arboreal	Arboreal gleaner	Diurnal	Resident	9.5
52	*Spinus lawrencei*	Passeriformes	Fringillidae	Granivore	Arboreal	Arboreal gleaner	Diurnal	Winter migrant	10.95
52	*Spinus tristis*	Passeriformes	Fringillidae	Granivore	Arboreal	Arboreal gleaner	Diurnal	Winter migrant	12.9
52	*Coccothraustes vespertinus*	Passeriformes	Fringillidae	Granivore	Arboreal	Arboreal gleaner	Diurnal	Resident	61.4
