# Supplementary material for: Classification and distribution of functional groups of birds and mammals in Mexico
Source: PLoS One. 2023 Nov 7;18(11):e0287036. doi: 10.1371/journal.pone.0287036 (PMC10629651; doi:10.1371/journal.pone.0287036)
Supplement: S2 Table — (PDF) [file pone.0287036.s002.pdf]

Appendix 2. Functional traits for 496 mammalian species distributed in Mexico and the Functional Groups assigned to them.

Functional group	Species	Order	Family	Feeding habit	Locomotion	Feeding substrate and technique	Activity period	Seasonality	Body size (g)
1	*Heteromys irroratus*	Rodentia	Heteromyidae	Granivore	Terrestrial	Ground browser	Nocturnal	Resident	42
1	*Heteromys pictus*	Rodentia	Heteromyidae	Granivore	Terrestrial	Ground browser	Nocturnal	Resident	55
1	*Heteromys salvini*	Rodentia	Heteromyidae	Granivore	Terrestrial	Ground browser	Nocturnal	Resident	56
1	*Heteromys spectabilis*	Rodentia	Heteromyidae	Granivore	Terrestrial	Ground browser	Nocturnal	Resident	56
1	*Chaetodipus arenarius*	Rodentia	Heteromyidae	Granivore	Terrestrial	Ground browser	Nocturnal	Resident	15
1	*Chaetodipus artus*	Rodentia	Heteromyidae	Granivore	Terrestrial	Ground browser	Nocturnal	Resident	20
1	*Chaetodipus baileyi*	Rodentia	Heteromyidae	Granivore	Terrestrial	Ground browser	Nocturnal	Resident	31
1	*Chaetodipus californicus*	Rodentia	Heteromyidae	Granivore	Terrestrial	Ground browser	Nocturnal	Resident	20
1	*Chaetodipus eremicus*	Rodentia	Heteromyidae	Granivore	Terrestrial	Ground browser	Nocturnal	Resident	16
1	*Chaetodipus fallax*	Rodentia	Heteromyidae	Granivore	Terrestrial	Ground browser	Nocturnal	Resident	19.5
1	*Chaetodipus formosus*	Rodentia	Heteromyidae	Granivore	Terrestrial	Ground browser	Nocturnal	Resident	21
1	*Chaetodipus goldmani*	Rodentia	Heteromyidae	Granivore	Terrestrial	Ground browser	Nocturnal	Resident	20
1	*Chaetodipus hispidus*	Rodentia	Heteromyidae	Granivore	Terrestrial	Ground browser	Nocturnal	Resident	37.5
1	*Chaetodipus intermedius*	Rodentia	Heteromyidae	Granivore	Terrestrial	Ground browser	Nocturnal	Resident	14.5
1	*Chaetodipus lineatus*	Rodentia	Heteromyidae	Granivore	Terrestrial	Ground browser	Nocturnal	Resident	17
1	*Chaetodipus nelsoni*	Rodentia	Heteromyidae	Granivore	Terrestrial	Ground browser	Nocturnal	Resident	14.25
1	*Chaetodipus penicillatus*	Rodentia	Heteromyidae	Granivore	Terrestrial	Ground browser	Nocturnal	Resident	16.5
1	*Chaetodipus pernix*	Rodentia	Heteromyidae	Granivore	Terrestrial	Ground browser	Nocturnal	Resident	17
1	*Chaetodipus rudinoris*	Rodentia	Heteromyidae	Granivore	Terrestrial	Ground browser	Nocturnal	Resident	26
1	*Chaetodipus spinatus*	Rodentia	Heteromyidae	Granivore	Terrestrial	Ground browser	Nocturnal	Resident	21
1	*Chaetodipus ammophilus*	Rodentia	Heteromyidae	Granivore	Terrestrial	Ground browser	Nocturnal	Resident	15
1	*Chaetodipus siccus*	Rodentia	Heteromyidae	Granivore	Terrestrial	Ground browser	Nocturnal	Resident	15
1	*Dipodomys compactus*	Rodentia	Heteromyidae	Granivore	Terrestrial	Ground browser	Nocturnal	Resident	77
1	*Dipodomys deserti*	Rodentia	Heteromyidae	Granivore	Terrestrial	Ground browser	Nocturnal	Resident	110.5
1	*Dipodomys gravipes*	Rodentia	Heteromyidae	Granivore	Terrestrial	Ground browser	Nocturnal	Resident	69
1	*Dipodomys merriami*	Rodentia	Heteromyidae	Granivore	Terrestrial	Ground browser	Nocturnal	Resident	42.5
1	*Dipodomys nelsoni*	Rodentia	Heteromyidae	Granivore	Terrestrial	Ground browser	Nocturnal	Resident	78.5
1	*Dipodomys ordii*	Rodentia	Heteromyidae	Granivore	Terrestrial	Ground browser	Nocturnal	Resident	73
1	*Dipodomys phillipsii*	Rodentia	Heteromyidae	Granivore	Terrestrial	Ground browser	Nocturnal	Resident	53
1	*Dipodomys simulans*	Rodentia	Heteromyidae	Granivore	Terrestrial	Ground browser	Nocturnal	Resident	61
1	*Dipodomys spectabilis*	Rodentia	Heteromyidae	Granivore	Terrestrial	Ground browser	Nocturnal	Resident	133.5
1	*Dipodomys ornatus*	Rodentia	Heteromyidae	Granivore	Terrestrial	Ground browser	Nocturnal	Resident	53
1	*Heteromys desmarestianus*	Rodentia	Heteromyidae	Granivore	Terrestrial	Ground browser	Nocturnal	Resident	72
1	*Heteromys gaumeri*	Rodentia	Heteromyidae	Granivore	Terrestrial	Ground browser	Nocturnal	Resident	56.5
1	*Heteromys nelsoni*	Rodentia	Heteromyidae	Granivore	Terrestrial	Ground browser	Nocturnal	Resident	56
1	*Perognathus amplus*	Rodentia	Heteromyidae	Granivore	Terrestrial	Ground browser	Nocturnal	Resident	9
1	*Perognathus flavescens*	Rodentia	Heteromyidae	Granivore	Terrestrial	Ground browser	Nocturnal	Resident	11.5
1	*Perognathus flavus*	Rodentia	Heteromyidae	Granivore	Terrestrial	Ground browser	Nocturnal	Resident	7.5
1	*Perognathus longimembris*	Rodentia	Heteromyidae	Granivore	Terrestrial	Ground browser	Nocturnal	Resident	8.5
1	*Perognathus merriami*	Rodentia	Heteromyidae	Granivore	Terrestrial	Ground browser	Nocturnal	Resident	8.5
1	*Megadontomys cryophilus*	Rodentia	Cricetidae	Granivore	Terrestrial	Ground browser	Nocturnal	Resident	58
1	*Megadontomys nelsoni*	Rodentia	Cricetidae	Granivore	Terrestrial	Ground browser	Nocturnal	Resident	58
1	*Megadontomys thomasi*	Rodentia	Cricetidae	Granivore	Terrestrial	Ground browser	Nocturnal	Resident	58
1	*Oligoryzomys fulvescens*	Rodentia	Cricetidae	Granivore	Terrestrial	Ground browser	Nocturnal	Resident	12

1	*Handleyomys alfaroi*	Rodentia	Cricetidae	Granivore	Terrestrial	Ground browser	Nocturnal	Resident	32
1	*Handleyomys chapmani*	Rodentia	Cricetidae	Granivore	Terrestrial	Ground browser	Nocturnal	Resident	24
1	*Oryzomys couesi*	Rodentia	Cricetidae	Granivore	Terrestrial	Ground browser	Nocturnal	Resident	60
1	*Handleyomys melanotis*	Rodentia	Cricetidae	Granivore	Terrestrial	Ground browser	Nocturnal	Resident	25
1	*Oryzomys palustris*	Rodentia	Cricetidae	Granivore	Terrestrial	Ground browser	Nocturnal	Resident	37
1	*Handleyomys saturatior*	Rodentia	Cricetidae	Granivore	Terrestrial	Ground browser	Nocturnal	Resident	370
1	*Peromyscus levipes*	Rodentia	Cricetidae	Granivore	Terrestrial	Ground browser	Nocturnal	Resident	20.5
1	*Peromyscus aztecus*	Rodentia	Cricetidae	Granivore	Terrestrial	Ground browser	Nocturnal	Resident	38.5
1	*Peromyscus boylii*	Rodentia	Cricetidae	Granivore	Terrestrial	Ground browser	Nocturnal	Resident	29
1	*Peromyscus bullatus*	Rodentia	Cricetidae	Granivore	Terrestrial	Ground browser	Nocturnal	Resident	28
1	*Peromyscus californicus*	Rodentia	Cricetidae	Granivore	Terrestrial	Ground browser	Nocturnal	Resident	44
1	*Peromyscus crinitus*	Rodentia	Cricetidae	Granivore	Terrestrial	Ground browser	Nocturnal	Resident	20.5
1	*Peromyscus difficilis*	Rodentia	Cricetidae	Granivore	Terrestrial	Ground browser	Nocturnal	Resident	28
1	*Peromyscus eremicus*	Rodentia	Cricetidae	Granivore	Terrestrial	Ground browser	Nocturnal	Resident	15.5
1	*Peromyscus eva*	Rodentia	Cricetidae	Granivore	Terrestrial	Ground browser	Nocturnal	Resident	16.5
1	*Peromyscus fraterculus*	Rodentia	Cricetidae	Granivore	Terrestrial	Ground browser	Nocturnal	Resident	15.5
1	*Peromyscus furvus*	Rodentia	Cricetidae	Granivore	Terrestrial	Ground browser	Nocturnal	Resident	16.5
1	*Peromyscus gratus*	Rodentia	Cricetidae	Granivore	Terrestrial	Ground browser	Nocturnal	Resident	25
1	*Peromyscus guatemalensis*	Rodentia	Cricetidae	Granivore	Terrestrial	Ground browser	Nocturnal	Resident	54
1	*Peromyscus gymnotis*	Rodentia	Cricetidae	Granivore	Terrestrial	Ground browser	Nocturnal	Resident	40.5
1	*Peromyscus hooperi*	Rodentia	Cricetidae	Granivore	Terrestrial	Ground browser	Nocturnal	Resident	24.5
1	*Peromyscus hylocetes*	Rodentia	Cricetidae	Granivore	Terrestrial	Ground browser	Nocturnal	Resident	29
1	*Peromyscus leucopus*	Rodentia	Cricetidae	Granivore	Terrestrial	Ground browser	Nocturnal	Resident	21.5
1	*Peromyscus maniculatus*	Rodentia	Cricetidae	Granivore	Terrestrial	Ground browser	Nocturnal	Resident	22.5
1	*Peromyscus megalops*	Rodentia	Cricetidae	Granivore	Terrestrial	Ground browser	Nocturnal	Resident	40
1	*Peromyscus mekisturus*	Rodentia	Cricetidae	Granivore	Terrestrial	Ground browser	Nocturnal	Resident	30.62
1	*Peromyscus melanocarpus*	Rodentia	Cricetidae	Granivore	Terrestrial	Ground browser	Nocturnal	Resident	59
1	*Peromyscus melanophrys*	Rodentia	Cricetidae	Granivore	Terrestrial	Ground browser	Nocturnal	Resident	42
1	*Peromyscus melanotis*	Rodentia	Cricetidae	Granivore	Terrestrial	Ground browser	Nocturnal	Resident	22.5
1	*Peromyscus melanurus*	Rodentia	Cricetidae	Granivore	Terrestrial	Ground browser	Nocturnal	Resident	30.62
1	*Peromyscus merriami*	Rodentia	Cricetidae	Granivore	Terrestrial	Ground browser	Nocturnal	Resident	17.5
1	*Peromyscus nasutus*	Rodentia	Cricetidae	Granivore	Terrestrial	Ground browser	Nocturnal	Resident	30.62
1	*Peromyscus ochraventer*	Rodentia	Cricetidae	Granivore	Terrestrial	Ground browser	Nocturnal	Resident	32
1	*Peromyscus pectoralis*	Rodentia	Cricetidae	Granivore	Terrestrial	Ground browser	Nocturnal	Resident	31.5
1	*Peromyscus polius*	Rodentia	Cricetidae	Granivore	Terrestrial	Ground browser	Nocturnal	Resident	29
1	*Peromyscus sagax*	Rodentia	Cricetidae	Granivore	Terrestrial	Ground browser	Nocturnal	Resident	29
1	*Peromyscus simulus*	Rodentia	Cricetidae	Granivore	Terrestrial	Ground browser	Nocturnal	Resident	29
1	*Peromyscus spicilegus*	Rodentia	Cricetidae	Granivore	Terrestrial	Ground browser	Nocturnal	Resident	29
1	*Peromyscus truei*	Rodentia	Cricetidae	Granivore	Terrestrial	Ground browser	Nocturnal	Resident	20
1	*Peromyscus winkelmanni*	Rodentia	Cricetidae	Granivore	Terrestrial	Ground browser	Nocturnal	Resident	50
1	*Peromyscus yucatanicus*	Rodentia	Cricetidae	Granivore	Terrestrial	Ground browser	Nocturnal	Resident	15.5
1	*Peromyscus zarhynchus*	Rodentia	Cricetidae	Granivore	Terrestrial	Ground browser	Nocturnal	Resident	67.5
1	*Peromyscus carletoni*	Rodentia	Cricetidae	Granivore	Terrestrial	Ground browser	Nocturnal	Resident	30.62
1	*Peromyscus dickeyi*	Rodentia	Cricetidae	Granivore	Terrestrial	Ground browser	Nocturnal	Resident	30.62
1	*Peromyscus guardia*	Rodentia	Cricetidae	Granivore	Terrestrial	Ground browser	Nocturnal	Resident	30.62
1	*Peromyscus interparietalis*	Rodentia	Cricetidae	Granivore	Terrestrial	Ground browser	Nocturnal	Resident	30.62
1	*Peromyscus madrensis*	Rodentia	Cricetidae	Granivore	Terrestrial	Ground browser	Nocturnal	Resident	30.62
1	*Peromyscus pembertoni*	Rodentia	Cricetidae	Granivore	Terrestrial	Ground browser	Nocturnal	Resident	30.62

1	*Peromyscus pseudocrinitus*	Rodentia	Cricetidae	Granivore	Terrestrial	Ground browser	Nocturnal	Resident	30.62
1	*Peromyscus schmidlyi*	Rodentia	Cricetidae	Granivore	Terrestrial	Ground browser	Nocturnal	Resident	29
1	*Peromyscus sejugis*	Rodentia	Cricetidae	Granivore	Terrestrial	Ground browser	Nocturnal	Resident	30.62
1	*Peromyscus slevini*	Rodentia	Cricetidae	Granivore	Terrestrial	Ground browser	Nocturnal	Resident	30.62
1	*Peromyscus stephani*	Rodentia	Cricetidae	Granivore	Terrestrial	Ground browser	Nocturnal	Resident	30.62
1	*Reithrodontomys bakeri*	Rodentia	Cricetidae	Granivore	Terrestrial	Ground browser	Nocturnal	Resident	19
1	*Reithrodontomys burti*	Rodentia	Cricetidae	Granivore	Terrestrial	Ground browser	Nocturnal	Resident	50.5
1	*Reithrodontomys hirsutus*	Rodentia	Cricetidae	Granivore	Terrestrial	Ground browser	Nocturnal	Resident	20
1	*Reithrodontomys megalotis*	Rodentia	Cricetidae	Granivore	Terrestrial	Ground browser	Nocturnal	Resident	12.5
1	*Reithrodontomys montanus*	Rodentia	Cricetidae	Granivore	Terrestrial	Ground browser	Nocturnal	Resident	9.5
1	*Reithrodontomys sumichrasti*	Rodentia	Cricetidae	Granivore	Terrestrial	Ground browser	Nocturnal	Resident	14
1	*Reithrodontomys tenuirostris*	Rodentia	Cricetidae	Granivore	Terrestrial	Ground browser	Nocturnal	Resident	17.8
1	*Reithrodontomys zacatecae*	Rodentia	Cricetidae	Granivore	Terrestrial	Ground browser	Nocturnal	Resident	11
1	*Reithrodontomys spectabilis*	Rodentia	Cricetidae	Granivore	Terrestrial	Ground browser	Nocturnal	Resident	17.8
2	*Sigmodon arizonae*	Rodentia	Cricetidae	Granivore	Terrestrial	Ground browser	Cathemeral	Resident	168
2	*Sigmodon mascotensis*	Rodentia	Cricetidae	Granivore	Terrestrial	Ground browser	Cathemeral	Resident	92
2	*Ammospermophilus harrisii*	Rodentia	Sciuridae	Granivore	Terrestrial	Ground browser	Diurnal	Resident	126
2	*Ammospermophilus interpres*	Rodentia	Sciuridae	Granivore	Terrestrial	Ground browser	Diurnal	Resident	110
2	*Ammospermophilus leucurus*	Rodentia	Sciuridae	Granivore	Terrestrial	Ground browser	Diurnal	Resident	120
2	*Neotamias dorsalis*	Rodentia	Sciuridae	Granivore	Terrestrial	Ground browser	Diurnal	Resident	67.5
2	*Neotamias merriami*	Rodentia	Sciuridae	Granivore	Terrestrial	Ground browser	Diurnal	Resident	47
2	*Neotamias obscurus*	Rodentia	Sciuridae	Granivore	Terrestrial	Ground browser	Diurnal	Resident	47
2	*Neotamias bulleri*	Rodentia	Sciuridae	Granivore	Terrestrial	Ground browser	Diurnal	Resident	70
2	*Neotamias durangae*	Rodentia	Sciuridae	Granivore	Terrestrial	Ground browser	Diurnal	Resident	4
3	*Cratogeomys castanops*	Rodentia	Geomyidae	Herbivore	Semi-fossorial	Underground browser	Nocturnal	Resident	271.5
3	*Cratogeomys fumosus*	Rodentia	Geomyidae	Herbivore	Semi-fossorial	Underground browser	Nocturnal	Resident	560
3	*Cratogeomys goldmani*	Rodentia	Geomyidae	Herbivore	Semi-fossorial	Underground browser	Nocturnal	Resident	306.5
3	*Cratogeomys merriami*	Rodentia	Geomyidae	Herbivore	Semi-fossorial	Underground browser	Nocturnal	Resident	420
3	*Cratogeomys fulvescens*	Rodentia	Geomyidae	Herbivore	Semi-fossorial	Underground browser	Nocturnal	Resident	420
3	*Cratogeomys perotensis*	Rodentia	Geomyidae	Herbivore	Semi-fossorial	Underground browser	Nocturnal	Resident	420
3	*Cratogeomys planiceps*	Rodentia	Geomyidae	Herbivore	Semi-fossorial	Underground browser	Nocturnal	Resident	425
3	*Geomys arenarius*	Rodentia	Geomyidae	Herbivore	Semi-fossorial	Underground browser	Nocturnal	Resident	209
3	*Geomys personatus*	Rodentia	Geomyidae	Herbivore	Semi-fossorial	Underground browser	Nocturnal	Resident	400
3	*Geomys tropicalis*	Rodentia	Geomyidae	Herbivore	Semi-fossorial	Underground browser	Nocturnal	Resident	300
3	*Orthogeomys grandis*	Rodentia	Geomyidae	Herbivore	Semi-fossorial	Underground browser	Nocturnal	Resident	830
3	*Heterogeomys hispidus*	Rodentia	Geomyidae	Herbivore	Semi-fossorial	Underground browser	Nocturnal	Resident	505.5
3	*Heterogeomys lanius*	Rodentia	Geomyidae	Herbivore	Semi-fossorial	Underground browser	Nocturnal	Resident	667
3	*Pappogeomys bulleri*	Rodentia	Geomyidae	Herbivore	Semi-fossorial	Underground browser	Nocturnal	Resident	122.5
3	*Thomomys bottae*	Rodentia	Geomyidae	Herbivore	Semi-fossorial	Underground browser	Cathemeral	Resident	160.5
3	*Thomomys umbrinus*	Rodentia	Geomyidae	Herbivore	Semi-fossorial	Underground browser	Nocturnal	Resident	135
3	*Thomomys atrovarius*	Rodentia	Geomyidae	Herbivore	Semi-fossorial	Underground browser	Nocturnal	Resident	135
3	*Thomomys nayarensis*	Rodentia	Geomyidae	Herbivore	Semi-fossorial	Underground browser	Nocturnal	Resident	135
3	*Thomomys nigricans*	Rodentia	Geomyidae	Herbivore	Semi-fossorial	Underground browser	Nocturnal	Resident	135
3	*Thomomys sheldoni*	Rodentia	Geomyidae	Herbivore	Semi-fossorial	Underground browser	Nocturnal	Resident	135
3	*Zygogeomys trichopus*	Rodentia	Geomyidae	Herbivore	Semi-fossorial	Underground browser	Nocturnal	Resident	545
4	*Ondatra zibethicus*	Rodentia	Cricetidae	Herbivore	Semi-aquatic	Aquatic surface browser	Cathemeral	Resident	1300
5	*Hodomys alleni*	Rodentia	Cricetidae	Herbivore	Semi-arboreal	Ground browser	Nocturnal	Resident	285.5
5	*Callospermophilus madrensis*	Rodentia	Sciuridae	Herbivore	Semi-arboreal	Ground browser	Diurnal	Resident	133.5

6	*Castor canadensis*	Rodentia	Castoridae	Herbivore	Semi-aquatic	Ground browser	Diurnal	Resident	25000
6	*Cynomys ludovicianus*	Rodentia	Sciuridae	Herbivore	Semi-fossorial	Ground browser	Diurnal	Resident	905
6	*Cynomys mexicanus*	Rodentia	Sciuridae	Herbivore	Semi-fossorial	Ground browser	Diurnal	Resident	1000
6	*Xerospermophilus spilosoma*	Rodentia	Sciuridae	Herbivore	Semi-fossorial	Ground browser	Diurnal	Resident	140
6	*Xerospermophilus tereticaudus*	Rodentia	Sciuridae	Herbivore	Semi-fossorial	Ground browser	Diurnal	Resident	140
6	*Otospermophilus variegatus*	Rodentia	Sciuridae	Herbivore	Semi-fossorial	Ground browser	Diurnal	Resident	725
7	*Lepus alleni*	Lagomorpha	Leporidae	Herbivore	Terrestrial	Ground browser	Nocturnal	Resident	4500
7	*Lepus californicus*	Lagomorpha	Leporidae	Herbivore	Terrestrial	Ground browser	Nocturnal	Resident	1500
7	*Lepus callotis*	Lagomorpha	Leporidae	Herbivore	Terrestrial	Ground browser	Nocturnal	Resident	2500
7	*Lepus flavigularis*	Lagomorpha	Leporidae	Herbivore	Terrestrial	Ground browser	Nocturnal	Resident	3500
7	*Sylvilagus audubonii*	Lagomorpha	Leporidae	Herbivore	Terrestrial	Ground browser	Nocturnal	Resident	1002.5
7	*Sylvilagus bachmani*	Lagomorpha	Leporidae	Herbivore	Terrestrial	Ground browser	Nocturnal	Resident	680
7	*Sylvilagus brasiliensis*	Lagomorpha	Leporidae	Herbivore	Terrestrial	Ground browser	Nocturnal	Resident	725
7	*Sylvilagus insonus*	Lagomorpha	Leporidae	Herbivore	Terrestrial	Ground browser	Nocturnal	Resident	1124
7	*Sylvilagus graysoni*	Lagomorpha	Leporidae	Herbivore	Terrestrial	Ground browser	Nocturnal	Resident	1124
7	*Sylvilagus robustus*	Lagomorpha	Leporidae	Herbivore	Terrestrial	Ground browser	Nocturnal	Resident	1500
7	*Sylvilagus mansuetus*	Lagomorpha	Leporidae	Herbivore	Terrestrial	Ground browser	Nocturnal	Resident	1124
7	*Cuniculus paca*	Rodentia	Cuniculidae	Herbivore	Terrestrial	Ground browser	Nocturnal	Resident	9000
7	*Baiomys taylori*	Rodentia	Cricetidae	Herbivore	Terrestrial	Ground browser	Nocturnal	Resident	7.5
7	*Microtus californicus*	Rodentia	Cricetidae	Herbivore	Terrestrial	Ground browser	Nocturnal	Resident	31
7	*Microtus guatemalensis*	Rodentia	Cricetidae	Herbivore	Terrestrial	Ground browser	Nocturnal	Resident	31
7	*Microtus mexicanus*	Rodentia	Cricetidae	Herbivore	Terrestrial	Ground browser	Nocturnal	Resident	34.5
7	*Microtus oaxacensis*	Rodentia	Cricetidae	Herbivore	Terrestrial	Ground browser	Nocturnal	Resident	31
7	*Microtus quasiater*	Rodentia	Cricetidae	Herbivore	Terrestrial	Ground browser	Nocturnal	Resident	28
7	*Microtus umbrosus*	Rodentia	Cricetidae	Herbivore	Terrestrial	Ground browser	Nocturnal	Resident	31
7	*Nelsonia goldmani*	Rodentia	Cricetidae	Herbivore	Terrestrial	Ground browser	Nocturnal	Resident	50
7	*Nelsonia neotomodon*	Rodentia	Cricetidae	Herbivore	Terrestrial	Ground browser	Nocturnal	Resident	49
7	*Neotoma albigula*	Rodentia	Cricetidae	Herbivore	Terrestrial	Ground browser	Nocturnal	Resident	172.5
7	*Neotoma angustapalata*	Rodentia	Cricetidae	Herbivore	Terrestrial	Ground browser	Nocturnal	Resident	210
7	*Neotoma goldmani*	Rodentia	Cricetidae	Herbivore	Terrestrial	Ground browser	Nocturnal	Resident	91
7	*Neotoma lepida*	Rodentia	Cricetidae	Herbivore	Terrestrial	Ground browser	Nocturnal	Resident	145
7	*Neotoma leucodon*	Rodentia	Cricetidae	Herbivore	Terrestrial	Ground browser	Nocturnal	Resident	172.5
7	*Neotoma macrotis*	Rodentia	Cricetidae	Herbivore	Terrestrial	Ground browser	Nocturnal	Resident	225
7	*Neotoma mexicana*	Rodentia	Cricetidae	Herbivore	Terrestrial	Ground browser	Nocturnal	Resident	177
7	*Neotoma micropus*	Rodentia	Cricetidae	Herbivore	Terrestrial	Ground browser	Nocturnal	Resident	202
7	*Neotoma nelsoni*	Rodentia	Cricetidae	Herbivore	Terrestrial	Ground browser	Nocturnal	Resident	177
7	*Neotoma palatina*	Rodentia	Cricetidae	Herbivore	Terrestrial	Ground browser	Nocturnal	Resident	198
7	*Neotoma phenax*	Rodentia	Cricetidae	Herbivore	Terrestrial	Ground browser	Nocturnal	Resident	233.5
7	*Neotoma bryanti*	Rodentia	Cricetidae	Herbivore	Terrestrial	Ground browser	Nocturnal	Resident	177
7	*Neotoma devia*	Rodentia	Cricetidae	Herbivore	Terrestrial	Ground browser	Nocturnal	Resident	145
7	*Neotoma insularis*	Rodentia	Cricetidae	Herbivore	Terrestrial	Ground browser	Nocturnal	Resident	145
7	*Neotoma melanura*	Rodentia	Cricetidae	Herbivore	Terrestrial	Ground browser	Nocturnal	Resident	172.5
7	*Neotoma picta*	Rodentia	Cricetidae	Herbivore	Terrestrial	Ground browser	Nocturnal	Resident	177
7	*Neotomodon alstoni*	Rodentia	Cricetidae	Herbivore	Terrestrial	Ground browser	Nocturnal	Resident	50
7	*Handleyomys rhabdops*	Rodentia	Cricetidae	Herbivore	Terrestrial	Ground browser	Nocturnal	Resident	37
7	*Handleyomys rostratus*	Rodentia	Cricetidae	Herbivore	Terrestrial	Ground browser	Nocturnal	Resident	42.5
7	*Reithrodontomys chrysopsis*	Rodentia	Cricetidae	Herbivore	Terrestrial	Ground browser	Nocturnal	Resident	19
7	*Sigmodon alleni*	Rodentia	Cricetidae	Herbivore	Terrestrial	Ground browser	Nocturnal	Resident	182

7	*Sigmodon fulviventer*	Rodentia	Cricetidae	Herbivore	Terrestrial	Ground browser	Nocturnal	Resident	210
7	*Sigmodon hispidus*	Rodentia	Cricetidae	Herbivore	Terrestrial	Ground browser	Nocturnal	Resident	210
7	*Sigmodon leucotis*	Rodentia	Cricetidae	Herbivore	Terrestrial	Ground browser	Nocturnal	Resident	113
7	*Sigmodon planifrons*	Rodentia	Cricetidae	Herbivore	Terrestrial	Ground browser	Nocturnal	Resident	182
7	*Sigmodon toltecus*	Rodentia	Cricetidae	Herbivore	Terrestrial	Ground browser	Nocturnal	Resident	210
7	*Sigmodon zanjonensis*	Rodentia	Cricetidae	Herbivore	Terrestrial	Ground browser	Nocturnal	Resident	182
8	*Romerolagus diazi*	Lagomorpha	Leporidae	Herbivore	Terrestrial	Ground browser	Cathemeral	Resident	494.25
8	*Sylvilagus cunicularius*	Lagomorpha	Leporidae	Herbivore	Terrestrial	Ground browser	Cathemeral	Resident	2050
8	*Sylvilagus floridanus*	Lagomorpha	Leporidae	Herbivore	Terrestrial	Ground browser	Cathemeral	Resident	1165
8	*Microtus pennsylvanicus*	Rodentia	Cricetidae	Herbivore	Terrestrial	Ground browser	Cathemeral	Resident	31
8	*Sigmodon ochrognathus*	Rodentia	Cricetidae	Herbivore	Terrestrial	Ground browser	Cathemeral	Resident	87
8	*Sigmodon hirsutus*	Rodentia	Cricetidae	Herbivore	Terrestrial	Ground browser	Cathemeral	Resident	210
8	*Mazama temama*	Artiodactyla	Cervidae	Herbivore	Terrestrial	Ground browser	Cathemeral	Resident	37500
8	*Mazama pandora*	Artiodactyla	Cervidae	Herbivore	Terrestrial	Ground browser	Cathemeral	Resident	21000
8	*Odocoileus hemionus*	Artiodactyla	Cervidae	Herbivore	Terrestrial	Ground browser	Cathemeral	Resident	95000
8	*Odocoileus virginianus*	Artiodactyla	Cervidae	Herbivore	Terrestrial	Ground browser	Cathemeral	Resident	81000
8	*Tapirella bairdii*	Perissodactyla	Tapiridae	Herbivore	Terrestrial	Ground browser	Cathemeral	Resident	225000
9	*Bison bison*	Artiodactyla	Bovidae	Herbivore	Terrestrial	Ground browser	Diurnal	Resident	900000
10	*Baiomys musculus*	Rodentia	Cricetidae	Herbivore	Terrestrial	Ground browser	Diurnal	Resident	8
10	*Otospermophilus beecheyi*	Rodentia	Sciuridae	Herbivore	Terrestrial	Ground browser	Diurnal	Resident	509
10	*Ictidomys mexicanus*	Rodentia	Sciuridae	Herbivore	Terrestrial	Ground browser	Diurnal	Resident	274.5
10	*Antilocapra americana*	Artiodactyla	Antilocapridae	Herbivore	Terrestrial	Ground browser	Diurnal	Resident	58500
10	*Ovis canadensis*	Artiodactyla	Bovidae	Herbivore	Terrestrial	Ground browser	Diurnal	Resident	70000
10	*Dicotyles tajacu*	Artiodactyla	Tayassuidae	Herbivore	Terrestrial	Ground browser	Diurnal	Resident	21928.57
10	*Tayassu pecari*	Artiodactyla	Tayassuidae	Herbivore	Terrestrial	Ground browser	Diurnal	Resident	38500
11	*Otonyctomys hatti*	Rodentia	Cricetidae	Granivore	Semi-arboreal	Ground browser	Nocturnal	Resident	59
11	*Reithrodontomys gracilis*	Rodentia	Cricetidae	Granivore	Semi-arboreal	Ground browser	Nocturnal	Resident	10.5
11	*Tamiasciurus mearnsi*	Rodentia	Sciuridae	Granivore	Semi-arboreal	Ground browser	Diurnal	Resident	226.5
12	*Habromys chinanteco*	Rodentia	Cricetidae	Granivore	Semi-arboreal	Arboreal browser	Nocturnal	Resident	19.5
12	*Habromys delicatulus*	Rodentia	Cricetidae	Granivore	Semi-arboreal	Arboreal browser	Nocturnal	Resident	14.5
12	*Habromys ixtlani*	Rodentia	Cricetidae	Granivore	Semi-arboreal	Arboreal browser	Nocturnal	Resident	40
12	*Habromys lepturus*	Rodentia	Cricetidae	Granivore	Semi-arboreal	Arboreal browser	Nocturnal	Resident	32
12	*Habromys lophurus*	Rodentia	Cricetidae	Granivore	Semi-arboreal	Arboreal browser	Nocturnal	Resident	32
12	*Habromys simulatus*	Rodentia	Cricetidae	Granivore	Semi-arboreal	Arboreal browser	Nocturnal	Resident	18
12	*Habromys schmidlyi*	Rodentia	Cricetidae	Granivore	Semi-arboreal	Arboreal browser	Nocturnal	Resident	12.5
12	*Peromyscus perfulvus*	Rodentia	Cricetidae	Granivore	Semi-arboreal	Arboreal browser	Nocturnal	Resident	32.5
12	*Reithrodontomys mexicanus*	Rodentia	Cricetidae	Granivore	Semi-arboreal	Arboreal browser	Nocturnal	Resident	19
12	*Glaucomys volans*	Rodentia	Sciuridae	Granivore	Arboreal	Arboreal browser	Nocturnal	Resident	65.5
12	*Sciurus griseus*	Rodentia	Sciuridae	Granivore	Semi-arboreal	Arboreal browser	Diurnal	Resident	551.8
12	*Sciurus nayaritensis*	Rodentia	Sciuridae	Granivore	Semi-arboreal	Arboreal browser	Diurnal	Resident	776
12	*Sciurus niger*	Rodentia	Sciuridae	Granivore	Semi-arboreal	Arboreal browser	Diurnal	Resident	964.5
12	*Sciurus oculatus*	Rodentia	Sciuridae	Granivore	Semi-arboreal	Arboreal browser	Diurnal	Resident	650
13	*Tylomys bullaris*	Rodentia	Cricetidae	Herbivore	Arboreal	Arboreal browser	Nocturnal	Resident	310.5
13	*Tylomys nudicaudus*	Rodentia	Cricetidae	Herbivore	Arboreal	Arboreal browser	Nocturnal	Resident	310.5
13	*Tylomys tumbalensis*	Rodentia	Cricetidae	Herbivore	Arboreal	Arboreal browser	Nocturnal	Resident	310.5
13	*Xenomys nelsoni*	Rodentia	Cricetidae	Herbivore	Arboreal	Arboreal browser	Nocturnal	Resident	115
14	*Erethizon dorsatum*	Rodentia	Erethizontidae	Herbivore	Semi-arboreal	Arboreal browser	Nocturnal	Resident	9500
14	*Osgoodomys banderanus*	Rodentia	Cricetidae	Herbivore	Semi-arboreal	Arboreal browser	Nocturnal	Resident	48.5

14	*Reithrodontomys microdon*	Rodentia	Cricetidae	Herbivore	Semi-arboreal	Arboreal browser	Nocturnal	Resident	20
14	*Sciurus aberti*	Rodentia	Sciuridae	Herbivore	Semi-arboreal	Arboreal browser	Diurnal	Resident	500
14	*Sciurus alleni*	Rodentia	Sciuridae	Herbivore	Semi-arboreal	Arboreal browser	Diurnal	Resident	400
14	*Sciurus arizonensis*	Rodentia	Sciuridae	Herbivore	Semi-arboreal	Arboreal browser	Diurnal	Resident	551.8
14	*Sciurus aureogaster*	Rodentia	Sciuridae	Herbivore	Semi-arboreal	Arboreal browser	Diurnal	Resident	561
14	*Sciurus colliaei*	Rodentia	Sciuridae	Herbivore	Semi-arboreal	Arboreal browser	Diurnal	Resident	435
14	*Sciurus variegatoides*	Rodentia	Sciuridae	Herbivore	Semi-arboreal	Arboreal browser	Diurnal	Resident	551.8
14	*Notocitellus adocetus*	Rodentia	Sciuridae	Herbivore	Semi-arboreal	Arboreal browser	Diurnal	Resident	206.5
14	*Notocitellus annulatus*	Rodentia	Sciuridae	Herbivore	Semi-arboreal	Arboreal browser	Diurnal	Resident	442.5
15	*Anoura geoffroyi*	Chiroptera	Phyllostomidae	Nectarivore	Volant	Arboreal browser	Nocturnal	Resident	15.2
15	*Choeroniscus godmani*	Chiroptera	Phyllostomidae	Nectarivore	Volant	Arboreal browser	Nocturnal	Resident	6.5
15	*Choeronycteris mexicana*	Chiroptera	Phyllostomidae	Nectarivore	Volant	Arboreal browser	Nocturnal	Resident	16.8
15	*Glossophaga commissarisi*	Chiroptera	Phyllostomidae	Nectarivore	Volant	Arboreal browser	Nocturnal	Resident	8.9
15	*Glossophaga leachii*	Chiroptera	Phyllostomidae	Nectarivore	Volant	Arboreal browser	Nocturnal	Resident	11
15	*Glossophaga morenoi*	Chiroptera	Phyllostomidae	Nectarivore	Volant	Arboreal browser	Nocturnal	Resident	8.5
15	*Glossophaga soricina*	Chiroptera	Phyllostomidae	Nectarivore	Volant	Arboreal browser	Nocturnal	Resident	9.6
15	*Hylonycteris underwoodi*	Chiroptera	Phyllostomidae	Nectarivore	Volant	Arboreal browser	Nocturnal	Resident	6.225
15	*Leptonycteris curasoae*	Chiroptera	Phyllostomidae	Nectarivore	Volant	Arboreal browser	Nocturnal	Resident	20
15	*Leptonycteris nivalis*	Chiroptera	Phyllostomidae	Nectarivore	Volant	Arboreal browser	Nocturnal	Resident	23
15	*Leptonycteris yerbabuenae*	Chiroptera	Phyllostomidae	Nectarivore	Volant	Arboreal browser	Nocturnal	Resident	20
15	*Lichonycteris obscura*	Chiroptera	Phyllostomidae	Nectarivore	Volant	Arboreal browser	Nocturnal	Resident	5.6
15	*Musonycteris harrisoni*	Chiroptera	Phyllostomidae	Nectarivore	Volant	Arboreal browser	Nocturnal	Resident	10
15	*Phyllostomus discolor*	Chiroptera	Phyllostomidae	Nectarivore	Volant	Arboreal browser	Nocturnal	Resident	40
16	*Artibeus hirsutus*	Chiroptera	Phyllostomidae	Frugivore	Volant	Arboreal browser	Nocturnal	Resident	39.5
16	*Artibeus jamaicensis*	Chiroptera	Phyllostomidae	Frugivore	Volant	Arboreal browser	Nocturnal	Resident	45
16	*Artibeus lituratus*	Chiroptera	Phyllostomidae	Frugivore	Volant	Arboreal browser	Nocturnal	Resident	51
16	*Carollia perspicillata*	Chiroptera	Phyllostomidae	Frugivore	Volant	Arboreal browser	Nocturnal	Resident	20.5
16	*Carollia sowelli*	Chiroptera	Phyllostomidae	Frugivore	Volant	Arboreal browser	Nocturnal	Resident	16.5
16	*Carollia subrufa*	Chiroptera	Phyllostomidae	Frugivore	Volant	Arboreal browser	Nocturnal	Resident	18
16	*Centurio senex*	Chiroptera	Phyllostomidae	Frugivore	Volant	Arboreal browser	Nocturnal	Resident	20
16	*Chiroderma salvini*	Chiroptera	Phyllostomidae	Frugivore	Volant	Arboreal browser	Nocturnal	Resident	28.15
16	*Chiroderma villosum*	Chiroptera	Phyllostomidae	Frugivore	Volant	Arboreal browser	Nocturnal	Resident	27.5
16	*Dermanura azteca*	Chiroptera	Phyllostomidae	Frugivore	Volant	Arboreal browser	Nocturnal	Resident	21
16	*Dermanura phaeotis*	Chiroptera	Phyllostomidae	Frugivore	Volant	Arboreal browser	Nocturnal	Resident	12
16	*Dermanura tolteca*	Chiroptera	Phyllostomidae	Frugivore	Volant	Arboreal browser	Nocturnal	Resident	17.5
16	*Dermanura watsoni*	Chiroptera	Phyllostomidae	Frugivore	Volant	Arboreal browser	Nocturnal	Resident	12
16	*Enchisthenes hartii*	Chiroptera	Phyllostomidae	Frugivore	Volant	Arboreal browser	Nocturnal	Resident	16
16	*Platyrrhinus helleri*	Chiroptera	Phyllostomidae	Frugivore	Volant	Arboreal browser	Nocturnal	Resident	15.5
16	*Sturnira lilium*	Chiroptera	Phyllostomidae	Frugivore	Volant	Arboreal browser	Nocturnal	Resident	18.5
16	*Sturnira ludovici*	Chiroptera	Phyllostomidae	Frugivore	Volant	Arboreal browser	Nocturnal	Resident	21.5
16	*Sturnira hondurensis*	Chiroptera	Phyllostomidae	Frugivore	Volant	Arboreal browser	Nocturnal	Resident	18.5
16	*Sturnira parvidens*	Chiroptera	Phyllostomidae	Frugivore	Volant	Arboreal browser	Nocturnal	Resident	18.5
16	*Uroderma bilobatum*	Chiroptera	Phyllostomidae	Frugivore	Volant	Arboreal browser	Nocturnal	Resident	17
16	*Uroderma magnirostrum*	Chiroptera	Phyllostomidae	Frugivore	Volant	Arboreal browser	Nocturnal	Resident	16.5
16	*Vampyressa thyone*	Chiroptera	Phyllostomidae	Frugivore	Volant	Arboreal browser	Nocturnal	Resident	7.9
16	*Vampyrodes caraccioli*	Chiroptera	Phyllostomidae	Frugivore	Volant	Arboreal browser	Nocturnal	Resident	10
16	*Vampyrodes major*	Chiroptera	Phyllostomidae	Frugivore	Volant	Arboreal browser	Nocturnal	Resident	10
17	*Dasyprocta mexicana*	Rodentia	Dasyproctidae	Frugivore	Terrestrial	Ground browser	Diurnal	Resident	3500

17	*Dasyprocta punctata*	Rodentia	Dasyproctidae	Frugivore	Terrestrial	Ground browser	Diurnal	Resident	2650
18	*Nyctomys sumichrasti*	Rodentia	Cricetidae	Frugivore	Semi-arboreal	Arboreal browser	Nocturnal	Resident	47.5
18	*Sciurus deppei*	Rodentia	Sciuridae	Frugivore	Semi-arboreal	Arboreal browser	Diurnal	Resident	250
18	*Sciurus yucatanensis*	Rodentia	Sciuridae	Frugivore	Semi-arboreal	Arboreal browser	Diurnal	Resident	430
18	*Bassariscus sumichrasti*	Carnivora	Procyonidae	Frugivore	Semi-arboreal	Arboreal browser	Nocturnal	Resident	1100
19	*Caluromys derbianus*	Didelphimorphia	Didelphidae	Frugivore	Arboreal	Arboreal browser	Nocturnal	Resident	307.5
19	*Coendou mexicanus*	Rodentia	Erethizontidae	Frugivore	Arboreal	Arboreal browser	Nocturnal	Resident	2000
19	*Ototylomys phyllotis*	Rodentia	Cricetidae	Frugivore	Arboreal	Arboreal browser	Nocturnal	Resident	100
19	*Potos flavus*	Carnivora	Procyonidae	Frugivore	Arboreal	Arboreal browser	Nocturnal	Resident	3000
19	*Alouatta palliata*	Primates	Atelidae	Frugivore	Arboreal	Arboreal browser	Diurnal	Resident	5775
19	*Alouatta villosa*	Primates	Atelidae	Frugivore	Arboreal	Arboreal browser	Diurnal	Resident	8893
19	*Ateles geoffroyi*	Primates	Atelidae	Frugivore	Arboreal	Arboreal browser	Diurnal	Resident	7267.5
20	*Scalopus aquaticus*	Soricomorpha	Talpidae	Insectivore	Fossorial	Underground hunter	Nocturnal	Resident	46
20	*Scapanus latimanus*	Soricomorpha	Talpidae	Insectivore	Fossorial	Underground hunter	Nocturnal	Resident	100
20	*Scapanus anthony*	Soricomorpha	Talpidae	Insectivore	Fossorial	Underground hunter	Nocturnal	Resident	100
21	*Tlacuatzin canescens*	Didelphimorphia	Didelphidae	Insectivore	Semi-arboreal	Arboreal hunter	Nocturnal	Resident	50
21	*Tamandua mexicana*	Pilosa	Myrmecophagidae	Insectivore	Semi-arboreal	Arboreal hunter	Cathemeral	Resident	4500
21	*Reithrodontomys fulvescens*	Rodentia	Cricetidae	Insectivore	Semi-arboreal	Arboreal hunter	Nocturnal	Resident	10
22	*Marmosa mexicana*	Didelphimorphia	Didelphidae	Insectivore	Arboreal	Arboreal hunter	Nocturnal	Resident	54.5
22	*Cyclopes didactylus*	Pilosa	Cyclopedidae	Insectivore	Arboreal	Arboreal hunter	Nocturnal	Resident	266
22	*Centronycteris centralis*	Chiroptera	Emballonuridae	Insectivore	Volant	Arboreal hunter	Nocturnal	Resident	5.5
22	*Glyphonycteris sylvestris*	Chiroptera	Phyllostomidae	Insectivore	Volant	Arboreal hunter	Nocturnal	Resident	9
22	*Lampronycteris brachyotis*	Chiroptera	Phyllostomidae	Insectivore	Volant	Arboreal hunter	Nocturnal	Resident	12
22	*Lophostoma evotis*	Chiroptera	Phyllostomidae	Insectivore	Volant	Arboreal hunter	Nocturnal	Resident	20
22	*Mimon cozumelae*	Chiroptera	Phyllostomidae	Insectivore	Volant	Arboreal hunter	Nocturnal	Resident	35
22	*Natalus stramineus*	Chiroptera	Natalidae	Insectivore	Volant	Arboreal hunter	Nocturnal	Resident	4.5
22	*Natalus mexicanus*	Chiroptera	Natalidae	Insectivore	Volant	Arboreal hunter	Nocturnal	Resident	4.5
23	*Peropteryx kappleri*	Chiroptera	Emballonuridae	Insectivore	Volant	Air hunter under canopy	Nocturnal	Resident	10
23	*Peropteryx macrotis*	Chiroptera	Emballonuridae	Insectivore	Volant	Air hunter under canopy	Nocturnal	Resident	5.75
23	*Rhynchonycteris naso*	Chiroptera	Emballonuridae	Insectivore	Volant	Air hunter under canopy	Nocturnal	Resident	3
23	*Saccopteryx bilineata*	Chiroptera	Emballonuridae	Insectivore	Volant	Air hunter under canopy	Nocturnal	Resident	8.9
23	*Saccopteryx leptura*	Chiroptera	Emballonuridae	Insectivore	Volant	Air hunter under canopy	Nocturnal	Resident	8.9
23	*Cynomops mexicanus*	Chiroptera	Molossidae	Insectivore	Volant	Air hunter under canopy	Nocturnal	Resident	1150
23	*Eumops auripendulus*	Chiroptera	Molossidae	Insectivore	Volant	Air hunter under canopy	Nocturnal	Resident	53
23	*Eumops perotis*	Chiroptera	Molossidae	Insectivore	Volant	Air hunter under canopy	Nocturnal	Resident	69
23	*Eumops underwoodi*	Chiroptera	Molossidae	Insectivore	Volant	Air hunter under canopy	Nocturnal	Resident	54
23	*Eumops ferox*	Chiroptera	Molossidae	Insectivore	Volant	Air hunter under canopy	Nocturnal	Resident	53
23	*Eumops nanus*	Chiroptera	Molossidae	Insectivore	Volant	Air hunter under canopy	Nocturnal	Resident	37.5
23	*Molossus aztecus*	Chiroptera	Molossidae	Insectivore	Volant	Air hunter under canopy	Nocturnal	Resident	14
23	*Molossus coibensis*	Chiroptera	Molossidae	Insectivore	Volant	Air hunter under canopy	Nocturnal	Resident	19.5
23	*Molossus rufus*	Chiroptera	Molossidae	Insectivore	Volant	Air hunter under canopy	Nocturnal	Resident	29
23	*Molossus sinaloae*	Chiroptera	Molossidae	Insectivore	Volant	Air hunter under canopy	Nocturnal	Resident	21
23	*Molossus alvarezi*	Chiroptera	Molossidae	Insectivore	Volant	Air hunter under canopy	Nocturnal	Resident	24
23	*Molossus pretiosus*	Chiroptera	Molossidae	Insectivore	Volant	Air hunter under canopy	Nocturnal	Resident	24
23	*Nyctinomops aurispinosus*	Chiroptera	Molossidae	Insectivore	Volant	Air hunter under canopy	Nocturnal	Resident	20
23	*Nyctinomops femorosaccus*	Chiroptera	Molossidae	Insectivore	Volant	Air hunter under canopy	Nocturnal	Resident	14.625
23	*Nyctinomops laticaudatus*	Chiroptera	Molossidae	Insectivore	Volant	Air hunter under canopy	Nocturnal	Resident	13
23	*Nyctinomops macrotis*	Chiroptera	Molossidae	Insectivore	Volant	Air hunter under canopy	Nocturnal	Resident	26

23	*Promops centralis*	Chiroptera	Molossidae	Insectivore	Volant	Air hunter under canopy	Nocturnal	Resident	23
23	*Tadarida brasiliensis*	Chiroptera	Molossidae	Insectivore	Volant	Air hunter under canopy	Nocturnal	Resident	9.5
23	*Mormoops megalophylla*	Chiroptera	Mormoopidae	Insectivore	Volant	Air hunter under canopy	Nocturnal	Resident	15
23	*Pteronotus gymnonotus*	Chiroptera	Mormoopidae	Insectivore	Volant	Air hunter under canopy	Nocturnal	Resident	14
23	*Pteronotus personatus*	Chiroptera	Mormoopidae	Insectivore	Volant	Air hunter under canopy	Nocturnal	Resident	8
23	*Noctilio albiventris*	Chiroptera	Noctilionidae	Insectivore	Volant	Air hunter under canopy	Nocturnal	Resident	70
23	*Lonchorhina aurita*	Chiroptera	Phyllostomidae	Insectivore	Volant	Air hunter under canopy	Nocturnal	Resident	16
23	*Lophostoma brasiliense*	Chiroptera	Phyllostomidae	Insectivore	Volant	Air hunter under canopy	Nocturnal	Resident	10
23	*Macrophyllum macrophyllum*	Chiroptera	Phyllostomidae	Insectivore	Volant	Air hunter under canopy	Nocturnal	Resident	7.5
23	*Phylloderma stenops*	Chiroptera	Phyllostomidae	Insectivore	Volant	Air hunter under canopy	Nocturnal	Resident	60
23	*Thyroptera tricolor*	Chiroptera	Thyropteridae	Insectivore	Volant	Air hunter under canopy	Nocturnal	Resident	4
23	*Lasiurus blossevillii*	Chiroptera	Vespertilionidae	Insectivore	Volant	Air hunter under canopy	Nocturnal	Resident	9.5
23	*Lasiurus seminolus*	Chiroptera	Vespertilionidae	Insectivore	Volant	Air hunter under canopy	Nocturnal	Resident	9.5
23	*Corynorhinus mexicanus*	Chiroptera	Vespertilionidae	Insectivore	Volant	Air hunter under canopy	Nocturnal	Resident	9
23	*Corynorhinus townsendii*	Chiroptera	Vespertilionidae	Insectivore	Volant	Air hunter under canopy	Nocturnal	Resident	9
23	*Eptesicus brasiliensis*	Chiroptera	Vespertilionidae	Insectivore	Volant	Air hunter under canopy	Nocturnal	Resident	10.5
23	*Eptesicus furinalis*	Chiroptera	Vespertilionidae	Insectivore	Volant	Air hunter under canopy	Nocturnal	Resident	8
23	*Eptesicus fuscus*	Chiroptera	Vespertilionidae	Insectivore	Volant	Air hunter under canopy	Nocturnal	Resident	17
23	*Euderma maculatum*	Chiroptera	Vespertilionidae	Insectivore	Volant	Air hunter under canopy	Nocturnal	Resident	18
23	*Idionycteris phyllotis*	Chiroptera	Vespertilionidae	Insectivore	Volant	Air hunter under canopy	Nocturnal	Resident	12
23	*Lasionycteris noctivagans*	Chiroptera	Vespertilionidae	Insectivore	Volant	Air hunter under canopy	Nocturnal	Resident	10
23	*Lasiurus borealis*	Chiroptera	Vespertilionidae	Insectivore	Volant	Air hunter under canopy	Nocturnal	Resident	6.69
23	*Lasiurus cinereus*	Chiroptera	Vespertilionidae	Insectivore	Volant	Air hunter under canopy	Nocturnal	Resident	56.085
23	*Lasiurus ega*	Chiroptera	Vespertilionidae	Insectivore	Volant	Air hunter under canopy	Nocturnal	Resident	15
23	*Lasiurus intermedius*	Chiroptera	Vespertilionidae	Insectivore	Volant	Air hunter under canopy	Nocturnal	Resident	10
23	*Lasiurus xanthinus*	Chiroptera	Vespertilionidae	Insectivore	Volant	Air hunter under canopy	Nocturnal	Resident	16
23	*Myotis ciliolabrum*	Chiroptera	Vespertilionidae	Insectivore	Volant	Air hunter under canopy	Nocturnal	Resident	3
23	*Myotis elegans*	Chiroptera	Vespertilionidae	Insectivore	Volant	Air hunter under canopy	Nocturnal	Resident	4
23	*Myotis evotis*	Chiroptera	Vespertilionidae	Insectivore	Volant	Air hunter under canopy	Nocturnal	Resident	8
23	*Myotis fortidens*	Chiroptera	Vespertilionidae	Insectivore	Volant	Air hunter under canopy	Nocturnal	Resident	6.5
23	*Myotis findleyi*	Chiroptera	Vespertilionidae	Insectivore	Volant	Air hunter under canopy	Nocturnal	Resident	5.5
23	*Myotis occultus*	Chiroptera	Vespertilionidae	Insectivore	Volant	Air hunter under canopy	Nocturnal	Resident	5
23	*Myotis peninsularis*	Chiroptera	Vespertilionidae	Insectivore	Volant	Air hunter under canopy	Nocturnal	Resident	5
23	*Myotis planiceps*	Chiroptera	Vespertilionidae	Insectivore	Volant	Air hunter under canopy	Nocturnal	Resident	7
23	*Myotis thysanodes*	Chiroptera	Vespertilionidae	Insectivore	Volant	Air hunter under canopy	Nocturnal	Resident	7.5
23	*Myotis velifer*	Chiroptera	Vespertilionidae	Insectivore	Volant	Air hunter under canopy	Nocturnal	Resident	8.5
23	*Myotis volans*	Chiroptera	Vespertilionidae	Insectivore	Volant	Air hunter under canopy	Nocturnal	Resident	6
23	*Myotis yumanensis*	Chiroptera	Vespertilionidae	Insectivore	Volant	Air hunter under canopy	Nocturnal	Resident	5.55
23	*Myotis lucifugus*	Chiroptera	Vespertilionidae	Insectivore	Volant	Air hunter under canopy	Nocturnal	Resident	5
23	*Myotis melanorhinus*	Chiroptera	Vespertilionidae	Insectivore	Volant	Air hunter under canopy	Nocturnal	Resident	4.053
23	*Nycticeius humeralis*	Chiroptera	Vespertilionidae	Insectivore	Volant	Air hunter under canopy	Nocturnal	Resident	9.7
23	*Parastrellus hesperus*	Chiroptera	Vespertilionidae	Insectivore	Volant	Air hunter under canopy	Nocturnal	Resident	3.794
23	*Perimyotis subflavus*	Chiroptera	Vespertilionidae	Insectivore	Volant	Air hunter under canopy	Nocturnal	Resident	5.844
23	*Rhogeessa aeneus*	Chiroptera	Vespertilionidae	Insectivore	Volant	Air hunter under canopy	Nocturnal	Resident	4
23	*Rhogeessa alleni*	Chiroptera	Vespertilionidae	Insectivore	Volant	Air hunter under canopy	Nocturnal	Resident	6.9
23	*Rhogeessa genowaysi*	Chiroptera	Vespertilionidae	Insectivore	Volant	Air hunter under canopy	Nocturnal	Resident	5
23	*Rhogeessa gracilis*	Chiroptera	Vespertilionidae	Insectivore	Volant	Air hunter under canopy	Nocturnal	Resident	3.5
23	*Rhogeessa mira*	Chiroptera	Vespertilionidae	Insectivore	Volant	Air hunter under canopy	Nocturnal	Resident	5

23	*Rhogeessa parvula*	Chiroptera	Vespertilionidae	Insectivore	Volant	Air hunter under canopy	Nocturnal	Resident	6.5
23	*Rhogeessa tumida*	Chiroptera	Vespertilionidae	Insectivore	Volant	Air hunter under canopy	Nocturnal	Resident	4
23	*Rhogeessa bickhami*	Chiroptera	Vespertilionidae	Insectivore	Volant	Air hunter under canopy	Nocturnal	Resident	5
24	*Pteronotus davyi*	Chiroptera	Mormoopidae	Insectivore	Volant	Ground hunter	Nocturnal	Resident	7.5
24	*Pteronotus parnellii*	Chiroptera	Mormoopidae	Insectivore	Volant	Ground hunter	Nocturnal	Resident	15
24	*Macrotus californicus*	Chiroptera	Phyllostomidae	Insectivore	Volant	Ground hunter	Nocturnal	Resident	11.7
24	*Macrotus waterhousii*	Chiroptera	Phyllostomidae	Insectivore	Volant	Ground hunter	Nocturnal	Resident	15.5
24	*Micronycteris microtis*	Chiroptera	Phyllostomidae	Insectivore	Volant	Ground hunter	Nocturnal	Resident	6.25
24	*Micronycteris schmidtorum*	Chiroptera	Phyllostomidae	Insectivore	Volant	Ground hunter	Nocturnal	Resident	6.25
24	*Micronycteris megalotis*	Chiroptera	Phyllostomidae	Insectivore	Volant	Ground hunter	Nocturnal	Resident	6.25
24	*Tonatia saurophila*	Chiroptera	Phyllostomidae	Insectivore	Volant	Ground hunter	Nocturnal	Resident	28.25
24	*Antrozous pallidus*	Chiroptera	Vespertilionidae	Insectivore	Volant	Ground hunter	Nocturnal	Resident	20.5
24	*Bauerus dubiaquercus*	Chiroptera	Vespertilionidae	Insectivore	Volant	Ground hunter	Nocturnal	Resident	15
25	*Balantiopteryx io*	Chiroptera	Emballonuridae	Insectivore	Volant	Air hunter above canopy	Nocturnal	Resident	4.35
25	*Balantiopteryx plicata*	Chiroptera	Emballonuridae	Insectivore	Volant	Air hunter above canopy	Nocturnal	Resident	5.8
25	*Diclidurus albus*	Chiroptera	Emballonuridae	Insectivore	Volant	Air hunter above canopy	Nocturnal	Resident	20.5
25	*Eumops bonariensis*	Chiroptera	Molossidae	Insectivore	Volant	Air hunter above canopy	Nocturnal	Resident	37.5
25	*Eumops hansae*	Chiroptera	Molossidae	Insectivore	Volant	Air hunter above canopy	Nocturnal	Resident	53
25	*Molossus molossus*	Chiroptera	Molossidae	Insectivore	Volant	Air hunter above canopy	Nocturnal	Resident	14
25	*Myotis albescens*	Chiroptera	Vespertilionidae	Insectivore	Volant	Air hunter above canopy	Nocturnal	Resident	5.5
25	*Myotis auriculus*	Chiroptera	Vespertilionidae	Insectivore	Volant	Air hunter above canopy	Nocturnal	Resident	5
25	*Myotis californicus*	Chiroptera	Vespertilionidae	Insectivore	Volant	Air hunter above canopy	Nocturnal	Resident	3.5
25	*Myotis keaysi*	Chiroptera	Vespertilionidae	Insectivore	Volant	Air hunter above canopy	Nocturnal	Resident	6.5
25	*Myotis nigricans*	Chiroptera	Vespertilionidae	Insectivore	Volant	Air hunter above canopy	Nocturnal	Resident	3.5
26	*Cryptotis alticola*	Soricomorpha	Soricidae	Insectivore	Semi-fossorial	Ground hunter	Cathemeral	Resident	12
26	*Cryptotis goldmani*	Soricomorpha	Soricidae	Insectivore	Semi-fossorial	Ground hunter	Cathemeral	Resident	9.5
26	*Cryptotis goodwini*	Soricomorpha	Soricidae	Insectivore	Semi-fossorial	Ground hunter	Cathemeral	Resident	20
26	*Cryptotis griseoventris*	Soricomorpha	Soricidae	Insectivore	Semi-fossorial	Ground hunter	Cathemeral	Resident	12
26	*Cryptotis magna*	Soricomorpha	Soricidae	Insectivore	Semi-fossorial	Ground hunter	Cathemeral	Resident	7
26	*Cryptotis mayensis*	Soricomorpha	Soricidae	Insectivore	Semi-fossorial	Ground hunter	Cathemeral	Resident	102.5
26	*Cryptotis merriami*	Soricomorpha	Soricidae	Insectivore	Semi-fossorial	Ground hunter	Cathemeral	Resident	20
26	*Cryptotis mexicana*	Soricomorpha	Soricidae	Insectivore	Semi-fossorial	Ground hunter	Cathemeral	Resident	8.2
26	*Cryptotis nelsoni*	Soricomorpha	Soricidae	Insectivore	Semi-fossorial	Ground hunter	Cathemeral	Resident	12
26	*Cryptotis obscura*	Soricomorpha	Soricidae	Insectivore	Semi-fossorial	Ground hunter	Cathemeral	Resident	4
26	*Cryptotis parva*	Soricomorpha	Soricidae	Insectivore	Semi-fossorial	Ground hunter	Cathemeral	Resident	4.5
26	*Cryptotis peregrina*	Soricomorpha	Soricidae	Insectivore	Semi-fossorial	Ground hunter	Cathemeral	Resident	12
26	*Cryptotis phillipsii*	Soricomorpha	Soricidae	Insectivore	Semi-fossorial	Ground hunter	Cathemeral	Resident	12
26	*Cryptotis lacandonensis*	Soricomorpha	Soricidae	Insectivore	Semi-fossorial	Ground hunter	Cathemeral	Resident	12
26	*Cryptotis tropicalis*	Soricomorpha	Soricidae	Insectivore	Semi-fossorial	Ground hunter	Cathemeral	Resident	4.5
26	*Megasorex gigas*	Soricomorpha	Soricidae	Insectivore	Semi-fossorial	Ground hunter	Cathemeral	Resident	10.5
26	*Notiosorex cockrumi*	Soricomorpha	Soricidae	Insectivore	Semi-fossorial	Ground hunter	Cathemeral	Resident	5.2
26	*Notiosorex crawfordi*	Soricomorpha	Soricidae	Insectivore	Semi-fossorial	Ground hunter	Cathemeral	Resident	4.65
26	*Notiosorex evotis*	Soricomorpha	Soricidae	Insectivore	Semi-fossorial	Ground hunter	Cathemeral	Resident	5.65
26	*Notiosorex villai*	Soricomorpha	Soricidae	Insectivore	Semi-fossorial	Ground hunter	Cathemeral	Resident	5.2
26	*Notiosorex tataticuli*	Soricomorpha	Soricidae	Insectivore	Semi-fossorial	Ground hunter	Cathemeral	Resident	5.2
26	*Sorex ixtlanensis*	Soricomorpha	Soricidae	Insectivore	Semi-fossorial	Ground hunter	Cathemeral	Resident	6.7
26	*Sorex orizabae*	Soricomorpha	Soricidae	Insectivore	Semi-fossorial	Ground hunter	Cathemeral	Resident	6.7
26	*Sorex salvini*	Soricomorpha	Soricidae	Insectivore	Semi-fossorial	Ground hunter	Cathemeral	Resident	6.7

26	*Sorex arizonae*	Soricomorpha	Soricidae	Insectivore	Semi-fossorial	Ground hunter	Cathemeral	Resident	7.2
26	*Sorex emarginatus*	Soricomorpha	Soricidae	Insectivore	Semi-fossorial	Ground hunter	Cathemeral	Resident	5.75
26	*Sorex macrodon*	Soricomorpha	Soricidae	Insectivore	Semi-fossorial	Ground hunter	Cathemeral	Resident	10.55
26	*Sorex milleri*	Soricomorpha	Soricidae	Insectivore	Semi-fossorial	Ground hunter	Cathemeral	Resident	12.85
26	*Sorex monticolus*	Soricomorpha	Soricidae	Insectivore	Semi-fossorial	Ground hunter	Cathemeral	Resident	5.5
26	*Sorex oreopolus*	Soricomorpha	Soricidae	Insectivore	Semi-fossorial	Ground hunter	Cathemeral	Resident	5
26	*Sorex ornatus*	Soricomorpha	Soricidae	Insectivore	Semi-fossorial	Ground hunter	Cathemeral	Resident	7.2
26	*Sorex saussurei*	Soricomorpha	Soricidae	Insectivore	Semi-fossorial	Ground hunter	Cathemeral	Resident	6.7
26	*Sorex sclateri*	Soricomorpha	Soricidae	Insectivore	Semi-fossorial	Ground hunter	Cathemeral	Resident	7.2
26	*Sorex stizodon*	Soricomorpha	Soricidae	Insectivore	Semi-fossorial	Ground hunter	Cathemeral	Resident	7.2
26	*Sorex ventralis*	Soricomorpha	Soricidae	Insectivore	Semi-fossorial	Ground hunter	Cathemeral	Resident	5
26	*Sorex veraepacis*	Soricomorpha	Soricidae	Insectivore	Semi-fossorial	Ground hunter	Cathemeral	Resident	7
27	*Cabassous centralis*	Cingulata	Dasypodidae	Insectivore	Terrestrial	Ground hunter	Nocturnal	Resident	2500
27	*Dasypus novemcinctus*	Cingulata	Dasypodidae	Insectivore	Terrestrial	Ground hunter	Nocturnal	Resident	5500
27	*Onychomys arenicola*	Rodentia	Cricetidae	Insectivore	Terrestrial	Ground hunter	Nocturnal	Resident	26
27	*Onychomys leucogaster*	Rodentia	Cricetidae	Insectivore	Terrestrial	Ground hunter	Nocturnal	Resident	45
27	*Onychomys torridus*	Rodentia	Cricetidae	Insectivore	Terrestrial	Ground hunter	Nocturnal	Resident	25
27	*Peromyscus beatae*	Rodentia	Cricetidae	Insectivore	Terrestrial	Ground hunter	Nocturnal	Resident	26
27	*Peromyscus mexicanus*	Rodentia	Cricetidae	Insectivore	Terrestrial	Ground hunter	Nocturnal	Resident	39.5
27	*Scotinomys teguina*	Rodentia	Cricetidae	Insectivore	Terrestrial	Ground hunter	Diurnal	Resident	15
27	*Conepatus leuconotus*	Carnivora	Mephitidae	Insectivore	Terrestrial	Ground hunter	Nocturnal	Resident	2700
27	*Conepatus semistriatus*	Carnivora	Mephitidae	Insectivore	Terrestrial	Ground hunter	Nocturnal	Resident	2450
27	*Mephitis macroura*	Carnivora	Mephitidae	Insectivore	Terrestrial	Ground hunter	Nocturnal	Resident	650
27	*Mephitis mephitis*	Carnivora	Mephitidae	Insectivore	Terrestrial	Ground hunter	Nocturnal	Resident	3250
27	*Spilogale gracilis*	Carnivora	Mephitidae	Insectivore	Terrestrial	Ground hunter	Nocturnal	Resident	488.5
27	*Spilogale putorius*	Carnivora	Mephitidae	Insectivore	Terrestrial	Ground hunter	Nocturnal	Resident	544
27	*Spilogale pygmaea*	Carnivora	Mephitidae	Insectivore	Terrestrial	Ground hunter	Nocturnal	Resident	600
27	*Spilogale angustifrons*	Carnivora	Mephitidae	Insectivore	Terrestrial	Ground hunter	Nocturnal	Resident	544
28	*Oryzomys nelsoni*	Rodentia	Cricetidae	Omnivore	Terrestrial	Ground browser	Nocturnal	Resident	37
28	*Peromyscus caniceps*	Rodentia	Cricetidae	Omnivore	Terrestrial	Ground browser	Nocturnal	Resident	30.62
28	*Nasua narica*	Carnivora	Procyonidae	Omnivore	Terrestrial	Ground browser	Diurnal	Resident	4000
28	*Ursus americanus*	Carnivora	Ursidae	Omnivore	Terrestrial	Ground browser	Cathemeral	Resident	88000
28	*Ursus arctos*	Carnivora	Ursidae	Omnivore	Terrestrial	Ground browser	Cathemeral	Resident	190000
29	*Canis latrans*	Carnivora	Canidae	Omnivore	Terrestrial	Ground hunter	Cathemeral	Resident	14000
29	*Urocyon cinereoargenteus*	Carnivora	Canidae	Omnivore	Terrestrial	Ground hunter	Nocturnal	Resident	5500
29	*Eira barbara*	Carnivora	Mustelidae	Omnivore	Terrestrial	Ground hunter	Nocturnal	Resident	4500
30	*Didelphis virginiana*	Didelphimorphia	Didelphidae	Omnivore	Semi-arboreal	Ground browser	Nocturnal	Resident	4700
30	*Didelphis marsupialis*	Didelphimorphia	Didelphidae	Omnivore	Semi-arboreal	Ground browser	Nocturnal	Resident	4200
30	*Metachirus nudicaudatus*	Didelphimorphia	Didelphidae	Omnivore	Semi-arboreal	Ground browser	Nocturnal	Resident	460
30	*Philander opossum*	Didelphimorphia	Didelphidae	Omnivore	Semi-arboreal	Arboreal browser	Nocturnal	Resident	450
30	*Bassariscus astutus*	Carnivora	Procyonidae	Omnivore	Semi-arboreal	Ground hunter	Nocturnal	Resident	1081
30	*Procyon lotor*	Carnivora	Procyonidae	Omnivore	Semi-arboreal	Ground browser	Nocturnal	Resident	6100
31	*Rheomys mexicanus*	Rodentia	Cricetidae	Piscivore	Semi-aquatic	Aquatic surface hunter	Nocturnal	Resident	40
31	*Rheomys thomasi*	Rodentia	Cricetidae	Piscivore	Semi-aquatic	Aquatic surface hunter	Nocturnal	Resident	27
31	*Lontra canadensis*	Carnivora	Mustelidae	Piscivore	Semi-aquatic	Aquatic surface hunter	Nocturnal	Resident	9500
31	*Lontra longicaudis*	Carnivora	Mustelidae	Piscivore	Semi-aquatic	Aquatic surface hunter	Diurnal	Resident	10000
32	*Chironectes minimus*	Didelphimorphia	Didelphidae	Piscivore	Terrestrial	Aquatic surface hunter	Nocturnal	Resident	289
32	*Procyon pygmaeus*	Carnivora	Procyonidae	Omnivore	Semi-arboreal	Aquatic surface hunter	Nocturnal	Resident	2500

32	*Noctilio leporinus*	Chiroptera	Noctilionidae	Piscivore	Volant	Aquatic surface hunter	Nocturnal	Resident	70
32	*Myotis vivesi*	Chiroptera	Vespertilionidae	Piscivore	Volant	Aquatic surface hunter	Nocturnal	Resident	5.5
33	*Desmodus rotundus*	Chiroptera	Phyllostomidae	Hematophagous	Volant	Ground hunter	Nocturnal	Resident	32.5
33	*Diaemus youngi*	Chiroptera	Phyllostomidae	Hematophagous	Volant	Ground hunter	Nocturnal	Resident	40
33	*Diphylla ecaudata*	Chiroptera	Phyllostomidae	Hematophagous	Volant	Ground hunter	Nocturnal	Resident	35
34	*Leopardus wiedii*	Carnivora	Felidae	Carnivore	Semi-arboreal	Ground hunter	Nocturnal	Resident	4000
34	*Chrotopterus auritus*	Chiroptera	Phyllostomidae	Carnivore	Volant	Ground hunter	Nocturnal	Resident	85.5
34	*Trachops cirrhosus*	Chiroptera	Phyllostomidae	Carnivore	Volant	Ground hunter	Nocturnal	Resident	36.5
34	*Vampyrum spectrum*	Chiroptera	Phyllostomidae	Carnivore	Volant	Ground hunter	Nocturnal	Resident	34
35	*Canis lupus*	Carnivora	Canidae	Carnivore	Terrestrial	Ground hunter	Nocturnal	Resident	51500
35	*Vulpes macrotis*	Carnivora	Canidae	Carnivore	Terrestrial	Ground hunter	Nocturnal	Resident	2500
35	*Herpailurus yagouaroundi*	Carnivora	Felidae	Carnivore	Terrestrial	Ground hunter	Nocturnal	Resident	6250
35	*Leopardus pardalis*	Carnivora	Felidae	Carnivore	Terrestrial	Ground hunter	Nocturnal	Resident	12250
35	*Lynx rufus*	Carnivora	Felidae	Carnivore	Terrestrial	Ground hunter	Nocturnal	Resident	18350
35	*Panthera onca*	Carnivora	Felidae	Carnivore	Terrestrial	Ground hunter	Nocturnal	Resident	102000
35	*Puma concolor*	Carnivora	Felidae	Carnivore	Terrestrial	Ground hunter	Nocturnal	Resident	74500
35	*Galictis vittata*	Carnivora	Mustelidae	Carnivore	Terrestrial	Ground hunter	Nocturnal	Resident	2000
35	*Mustela frenata*	Carnivora	Mustelidae	Carnivore	Terrestrial	Ground hunter	Cathemeral	Resident	400
35	*Mustela nigripes*	Carnivora	Mustelidae	Carnivore	Terrestrial	Ground hunter	Nocturnal	Resident	800
35	*Taxidea taxus*	Carnivora	Mustelidae	Carnivore	Terrestrial	Ground hunter	Cathemeral	Resident	8000
