# Supplementary material for: Classification and distribution of functional groups of birds and mammals in Mexico
Source: PLoS One. 2023 Nov 7;18(11):e0287036. doi: 10.1371/journal.pone.0287036 (PMC10629651; doi:10.1371/journal.pone.0287036)
Supplement: S1 Text — (DOCX) [file pone.0287036.s003.docx]

**S1 Text**. Description of each functional group of birds and mammals in Mexico.

**Functional groups of birds**

***Invertivores*** (16 FG). FG1*: Arboreal hunter, migrant*- arboreal species that feed on insects on the trees, and can be found in winter (e.g., Western tanager, *Piranga ludoviciana*). FG2: *Arboreal hunter, transient* - arboreal species that feed on insects on the trees, and are transient in Mexico (e.g., Cerulean warbler, *Setophaga cerulea*). FG4: *Arboreal hunter, resident*- arboreal species that feed on insects on the trees, and can be observed year-round (e.g., Acorn woodpecker, *Melanerpes formicivorus*). FG5: *Volant, arboreal hunter*- species that, during the flight, catch and feed on insects on trees (e.g., Great antshrike, *Taraba major*). FG6: *Semi-arboreal, arboreal hunter*- semi-arboreal species that feed on insects mainly on the trees (e.g., House wren, *Troglodytes aedon*). FG12: *Semi-aquatic hunter*- species associated with water ecosystems and feed on insects (e.g., Upland sandpiper, *Bartramia longicauda*). FG13: *Semi-arboreal, ground hunter, resident*- semi-arboreal species that feed on insects mainly on the ground, and can be observed year-round (e.g., Gilded flicker, *Colaptes chrysoides*). FG14: *Semi-arboreal, ground hunter, migrant*- semi-arboreal species that feed on insects mainly on the ground, and can be found in winter (e.g., Northern flicker, *Colaptes auratus*). FG15: *Ground hunter, migrant*- terrestrial species that feed on insects on the ground, and can be observed in winter (e.g., Black-headed nightingale-thrush, *Passerina amoena*). FG16: *Ground hunter, resident*- terrestrial species that feed on insects on the ground, and can be found year-round (e.g., Black-headed nightingale-thrush, *Catharus mexicanus*). FG17: *Air hunter under canopy, resident*- species that, during the flight, feed mainly on insects caught in the air under the tree canopy, and can be found year-round (e.g., Scarlet flycatcher, *Pyrocephalus rubinus*). FG18: *Air hunter under canopy- transient*- species that, during the flight, feed mainly on insects caught in the air under the tree canopy, and are transient in Mexico (e.g., Gray kingbird, *Tyrannus dominicensis*). FG19: *Air hunter under canopy, migrant*- species that, during the flight, feed mainly on insects caught in the air under the tree canopy, and can be found in the country in winter (e.g., Least flycatcher, *Empidonax minimus*). FG20: *Air hunter above canopy*- species that, during the flight, feed mainly on insects caught in the air above the tree canopy (e.g., Sinaloa martin, *Progne sinaloae*). FG21: *Volant, ground hunter, nocturnal, resident*- species that, during their night flight, feed primarily on insects caught on the ground (e.g., Eastern screech owl, *Megascops asio*). FG22: *Volant, ground hunter, mixed activity period, mixed seasonality*- species that, during the flight, feed primarily on insects caught on the ground, with diurnal or cathemeral species, and can be resident or migrant (e.g., Blue-capped motmot, *Momotus coeruliceps*).

***Carnivores*** (6 FG). FG3: *Arboreal hunter*- species that feed on vertebrates in the tree canopy (e.g., Harpy eagle, *Harpia harpyja*). FG7: *Air hunter under canopy, migrant*- species that, during the flight, feed on other vertebrates caught in the air mainly between the tree canopy and the ground, and can be observed in winter (e.g., Cooper's hawk, *Accipiter cooperii*). FG8: *Air hunter under canopy, resident*- species that, during the flight, feed on other vertebrates caught between the tree canopy and the ground, and can be found year-round (e.g., Bicolored hawk, *Accipiter bicolor*). FG9: *Volant, ground hunter, diurnal, migrant*- species that, during the flight, hunt other vertebrates that are on the ground, during the day, and can be found in winter (e.g., Swainson's hawk, *Buteo swainsoni*). FG10: *Volant, ground hunter, nocturnal*- species that, during their night flight, hunt other vertebrates that are on the ground (e.g., Fulvous owl, *Strix fulvescens*). FG11: *Volant, ground hunter, diurnal, resident*- species that, during the flight, hunt other vertebrates that are on the ground, during the day, and can be found year-round (e.g., Black-and-white hawk-eagle, *Spizaetus melanoleucus*).

***Herbivores*** (5 FG). FG23: *Semi-aquatic, ground gleaner*- semi-aquatic species that eat different parts of plants mostly on the ground (e.g., Greater white-fronted goose, *Anser albifrons*). FG24: *Ground gleaner*- terrestrial species that eat different parts of plants on the ground (e.g., Ocellated turkey, *Meleagris ocellata*). FG26: *Semi-aquatic, aquatic gleaner, resident*- semi-aquatic species that eat different parts of plants mostly on the water surface, and can be found in the country all year round (e.g., American coot, *Fulica americana)*. FG27: *Semi-aquatic, aquatic gleaner, migrant*- semi-aquatic species that eat different parts of plants mostly on the water surface (e.g., Canvasback, *Aythya valisineria*). FG28: *Semi-aquatic, aquatic gleaner, accidental*- semi-aquatic species that eat different parts of plants during the day and mostly on the water surface, and are accidental (e.g., Tundra swan, *Cygnus columbianus).*

***Aquatic vertivores/ invertivores*** (9 FG). FG29: *Wader, cathemeral*- species that capture aquatic invertebrates in the mud or sand, during the day or night (e.g., Piping plover, *Charadrius melodus*). FG30: *Wader, diurnal, migrant*- species that capture aquatic invertebrates in the mud or sand during the day and can be found in the country in winter (e.g., Spotted sandpiper, *Actitis macularius*). FG31: *Wader, diurnal, mixed seasonality*- species that capture aquatic invertebrates in the mud or sand during the day, and are resident, transient or accidental (e.g., American avocet, *Recurvirostra americana*). FG32: *Volant, aquatic surface hunter, resident*- species that during flight hunt fish that are on the water surface, and can be found year-round (e.g., Ringed kingfisher, *Megaceryle torquata*). FG33: *Volant, aquatic surface hunter, migrant*- species that during flight, hunt fish that are on the water surface, and can be found in winter (e.g., Osprey, *Pandion haliaetus*). FG34: *Semi-aquatic hunter, resident* - species that feed on fish or aquatic invertebrates found on the water surface, either from the shore of the water body or by plunging, during the day, and can be found year-round (e.g., Tricolored heron, *Egretta tricolor*). FG35: *Semi-aquatic hunter, large*- large-body size species (> 4500 g) that feed on fish or aquatic invertebrates found on the water surface, either from the shore of the water body or by plunging (e.g., American white pelican, *Pelecanus erythrorhynchos*). FG36: *Semi-aquatic hunter, cathemeral*- species that feed on fish or aquatic invertebrates found on the water surface, either from the shore of the water body or by plunging, during the day or night, and can be found in winter (e.g., Common merganser, *Mergus merganser*). FG37: *Semi-aquatic hunter, migrant*- species that feed on fish or aquatic invertebrates found on the water surface, either from the shore of the water body or by plunging, during the day, and can be found mainly in winter (e.g., Lesser scaup, *Aythya affinis*).

***Granivores*** (5 FG). FG38: *Ground gleaner, migrant*- terrestrial species that glean grains and seeds on the ground, and can be found in winter (e.g., White-throated sparrow, *Zonotrichia albicollis*). FG39: *Ground gleaner, resident*- terrestrial species that glean grains and seeds on the ground, and can be found year-round (e.g., yellow-eyed junco, *Junco phaeonotus*). FG40: *Semi-aquatic gleaner*- semi-aquatic species that glean grains and seeds on the ground or the water surface (e.g., Black-bellied whistling duck, *Dendrocygna autumnalis*). FG41: *Semi-arboreal gleaner*- species that glean grains and seeds on both the ground and trees (e.g., Northern cardinal, *Cardinalis cardinalis*). FG52: *Arboreal gleaner*- species that glean grains and seeds on trees (e.g., Pine siskin, *Spinus pinus*).

***Scavengers*** (1 FG). FG43: *Scavenger*- species that feed on carrion (e.g., King vulture, *Sarcoramphus papa*).

***Nectarivores*** (3 FG). FG44: *Arboreal*- arboreal species that eat nectar from flowers (e.g., Red-legged honeycreeper (*Cyanerpes cyaneus*). FG45: *Volant, migrant*- species that eat nectar from flowers while flying, and can be found mainly in winter (e.g., Costa's hummingbird, *Calypte costae*). FG46: *Volant, resident*- species that eat nectar from flowers while flying, and can be found year-round (e.g., Mexican sheartail, *Doricha eliza*).

***Frugivores*** (4 groups). FG47: *Arboreal gleaner*- arboreal species that eat fruits that are on the trees (e.g., Collared aracari, *Pteroglossus torquatus*). FG48: *Semi-arboreal, arboreal gleaner*- semi-arboreal species that eat fruits that are mostly on the trees (e.g., Rufous-bellied chachalaca, *Ortalis wagleri*). FG49: *Ground gleaner*- species that eat fruits that are on the ground (e.g., Great tinamou, *Tinamus major*). FG50: *Semi-arboreal, ground gleaner*- semi-arboreal species that eat fruits that are mostly on the ground (e.g., Great curassow, *Crax rubra*).

***Omnivores*** (3 FG). FG25: *Semi-aquatic gleaner*- species that feed from plants and animals obtained on the water surface or on the ground (e.g., Whooping crane, *Grus americana*). FG42: *Ground gleaner*- species that feed from plants and animals obtained from the ground (e.g., Greater roadrunner, *Geococcyx californianus*). FG51: *Arboreal gleaner*- species that feed from plants and animals obtained on trees (e.g., White-throated magpie-jay, *Calocitta formosa*).

**Functional groups of mammals**

***Granivores*** (4 FG). FG1: *Ground browser, nocturnal*- ground-dwelling nocturnal terrestrial species that feed on seeds (e.g., Phillips's kangaroo rat, *Dipodomys phillipsii*). FG2: *Ground browser, diurnal*- ground-dwelling diurnal terrestrial species that feed on seeds (e.g., White-tailed antelope squirrel, *Ammospermophilus leucurus*). FG11: *Semi-arboreal, ground browser*- semi-arboreal species that feed on seeds mainly on the ground (e.g., Mearns's squirrel, *Tamiasciurus mearnsi*). FG12: *Semi-arboreal, arboreal browser*- semi-arboreal species that feed on seeds mainly on trees (e.g., Mexican fox squirrel, *Sciurus nayaritensis*).

***Herbivores*** (10 FG). FG3: *Semi-fossorial, underground browser*- semi-fossorial species that feed on parts of plants that are underground (e.g., Goldman's pocket gopher, *Cratogeomys goldmani*). FG4: *Semi-aquatic browser*- semi-aquatic species that feed on parts of plants mainly on the water surface (e.g., Muskrat, *Ondatra zibethicus*). FG5: *Semi-arboreal, ground browser*- semi-arboreal species that feed on different parts of plants mostly on the ground (e.g., Allen's woodrat, *Hodomys alleni*). FG6: *Semi-fossorial/semi-aquatic, ground browser*- semi-fossorial or semi-aquatic species that feed on parts of plants that are on the ground (e.g., Mexican prairie dog, *Cynomys mexicanus*). FG7: *Ground browser/grazer, nocturnal*- nocturnal terrestrial species that feed on different parts of plants on the ground (e.g., Allen's cotton rat, *Sigmodon alleni*). FG8: *Ground browser/grazer, cathemeral*- terrestrial species that eat different parts of plants on the ground (e.g., Central American red brocket, *Mazama temama*). FG9: *Ground browser/grazer, large*- large-body size terrestrial species (> 900 000 g) that feed on different parts of plants on the ground (e.g., American bison, *Bison bison*). FG10: *Ground browser/grazer, diurnal* – diurnal terrestrial species that feed on different parts of plants on the ground (e.g., White-lipped peccary, *Tayassu pecari*). FG13: *Arboreal browser*- species that feed on different parts of plants on the trees (e.g., Chiapan climbing rat, *Tylomys bullaris*). FG14: *Semi-arboreal, arboreal browser*- semi-arboreal species that feed on different parts of plants mostly on trees (e.g., Allen's squirrel, *Sciurus alleni*).

***Nectarivores*** (1 FG). FG15: *Nectarivores*- species that feed on nectar from flowers (e.g., Western long-tongued bat, *Glossophaga morenoi*).

***Frugivores*** (4 FG). FG16: *Aerial browser*- species that feed on fruits while flying (e.g., Hairy fruit-eating bat, *Artibeus hirsutus*). FG17: *Ground browser*- ground-dwelling species that feed on fruits (e.g., Central American agouti, *Dasyprocta punctata*). FG18: *Semi-arboreal browser*- semi-arboreal species that feed on fruits that are primarily on trees (e.g., Sumichrast's vesper rat, *Nyctomys sumichrasti*). FG19: *Arboreal browser*- arboreal species that feed on fruits that are on trees (e.g., Mantled howler, *Alouatta palliata*).

***Invertivores*** (8 FG). FG20*: Fossorial hunter*- species that live underground and feed on insects (e.g., Northern broad-footed mole, *Scapanus latimanus*). FG21: *Semi-arboreal hunter*- semi-arboreal species that feed on insects on trees or on the ground (e.g., Northern tamandua, *Tamandua mexicana*). FG22: *Arboreal hunter*- arboreal or volant species that feed on insects on trees (e.g., Cozumelan golden bat, *Mimon cozumelae*). FG23: *Air hunter under canopy*- species that, during the flight, feed on insects between the ground and the tree canopy (e.g., Slender yellow bat, *Rhogeessa gracilis*). FG24: *Volant, ground hunter*- species that, during the flight, feed primarily on arthropods on the ground (e.g., Pallid bat, *Antrozous pallidus*). FG25: *Air hunter above the canopy*- species that, during the flight, feed mainly on insects above the tree canopy (e.g., Velvety free-tailed bat, *Molossus molossus*). FG26*: Semi-fossorial hunter*- species adapted to excavate that feed on insects on the ground (e.g., Orizaba long-tailed shrew, *Sorex orizabae*). FG27: *Ground hunter*- terrestrial species that feed on insects on the ground (e.g., Striped skunk, *Mephitis mephitis*).

***Omnivores*** (3 FG). FG28: *Ground browser*- species that feed from plants and animals on the ground, with a significant preference for plants (e.g., American black bear, *Ursus americanus*). FG29: *Ground hunter*- species that feed from plants and animals on the ground, with a preference for animals (e.g., Gray fox, *Urocyon cinereoargenteus*). FG30: *Semi-arboreal browser*- species that feed from plants and animals obtained on trees, on the ground or even on the water surface (e.g., Virginia opossum, *Didelphis virginiana*).

***Aquatic vertivores/ invertivores*** (2 FG). FG31: *Semi-aquatic hunter*- semi-aquatic species that feed on fish or aquatic invertebrates found on the water surface (e.g., North American river otter, *Lontra canadensis*). FG32: *Terrestrial/volant aquatic hunter*- terrestrial o volant species that feed on fish or aquatic invertebrates found on the water surface (e.g., Fish-eating bat, *Myotis vivesi*).

***Hematophagous*** (1 FG). FG33: *Hematophagous*- species that feed on vertebrate blood (e.g., Common vampire bat, *Desmodus rotundus*).

***Carnivores*** (2 FG). FG34: *Semi-arboreal/Volant hunter*- semi-arboreal or volant species that feed on vertebrates that live on the ground (e.g., Spectral bat, *Vampyrum spectrum*). FG35: *Ground hunter*- terrestrial species that feed on vertebrates that live on the ground (e.g., Jaguar, *Panthera onca*).
