# Supplementary material for: Classification and distribution of functional groups of birds and mammals in Mexico
Source: PLoS One. 2023 Nov 7;18(11):e0287036. doi: 10.1371/journal.pone.0287036 (PMC10629651; doi:10.1371/journal.pone.0287036)

**S1 Maps.** Species richness map of each functional group of birds and mammals in Mexico.

**SUMMARY**

FUNCTIONAL GROUPS OF BIRDS…………………………………………. 2

Invertivores…………………………………………………………….. 2

Carnivores…………………………………………………………….... 4

Herbivores…………………………….………………………………... 5

Aquatic vertivore/invertivore..…………………………………………. 5

Granivores……………………………………………………………… 6

Scavengers……………………………………………………………… 7

Nectarivores……………………………………………………………. 7

Frugivores……………………………………………………………… 8

Omnivores……………………………………………………………… 9

FUNCTIONAL GROUPS OF MAMMALS…………………………………… 10

Granivores…………………………………………………………….... 10

Herbivores…………………………………………………………….... 11

Nectarivores……………………………………………………………. 12

Frugivores…………………………………………………………….... 12

Invertivores……………………………………………………………... 13

Omnivores…………………………………………………………….... 14

Aquatic vertivore/invertivore………………………………………...... 14

Hematophagous………………………………………………………… 15

Carnivores…………………………………………………………….... 15

FUNCTIONAL GROUPS SHARED BY BIRDS AND MAMMALS…………. 16

**FUNCTIONAL GROUPS OF BIRDS**


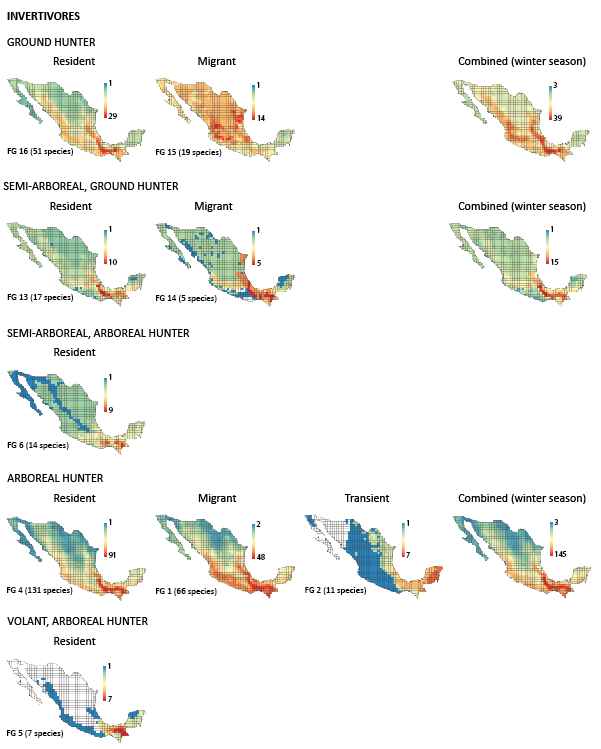


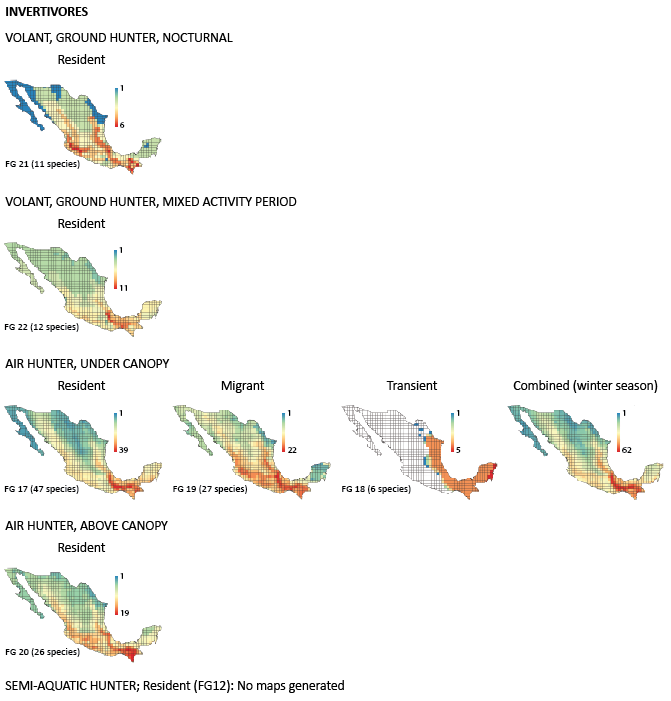


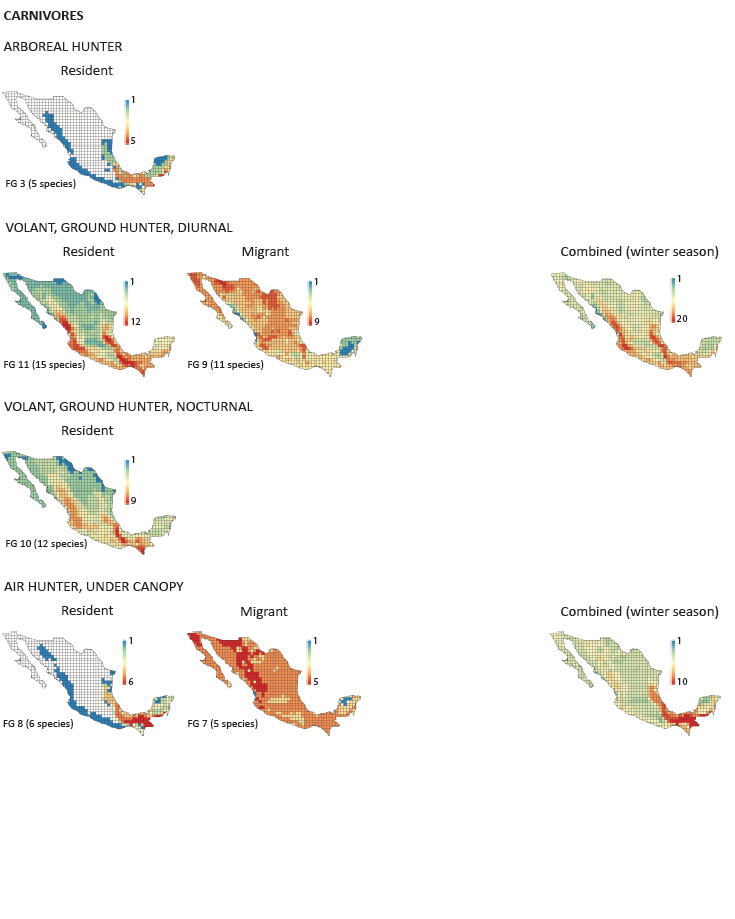


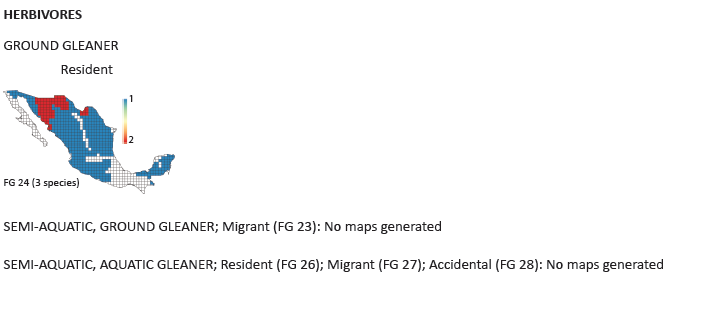


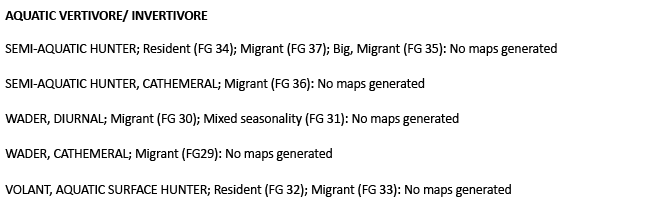


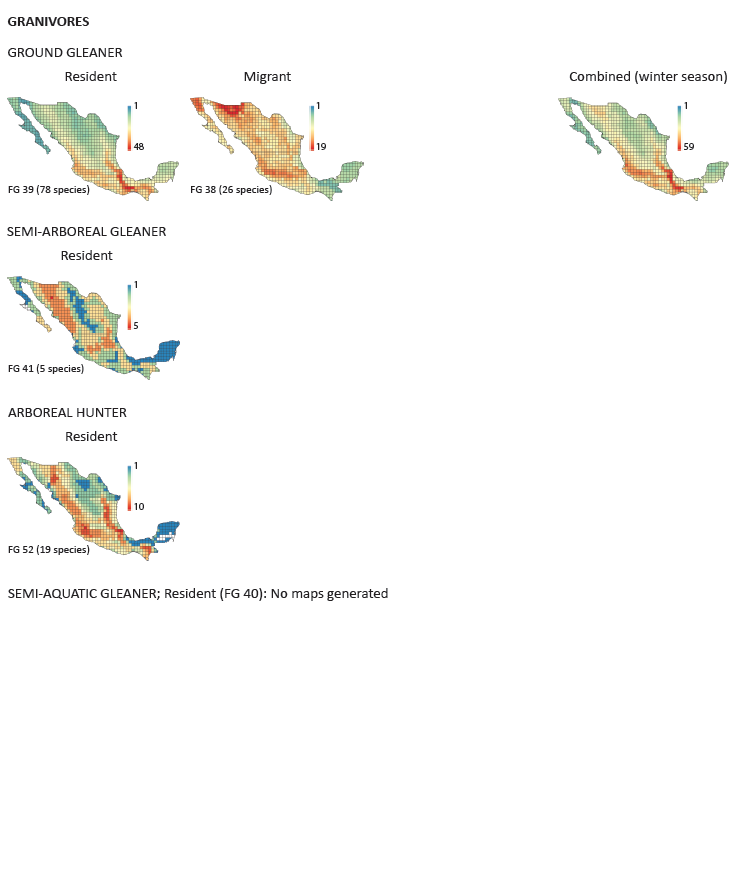


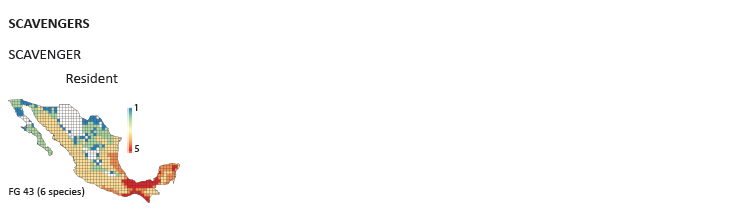


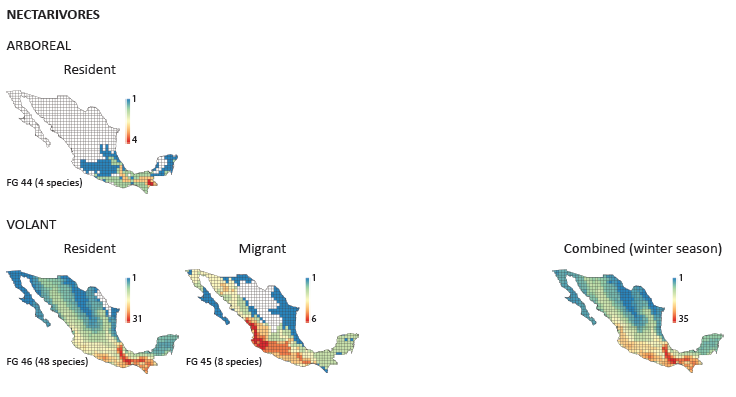


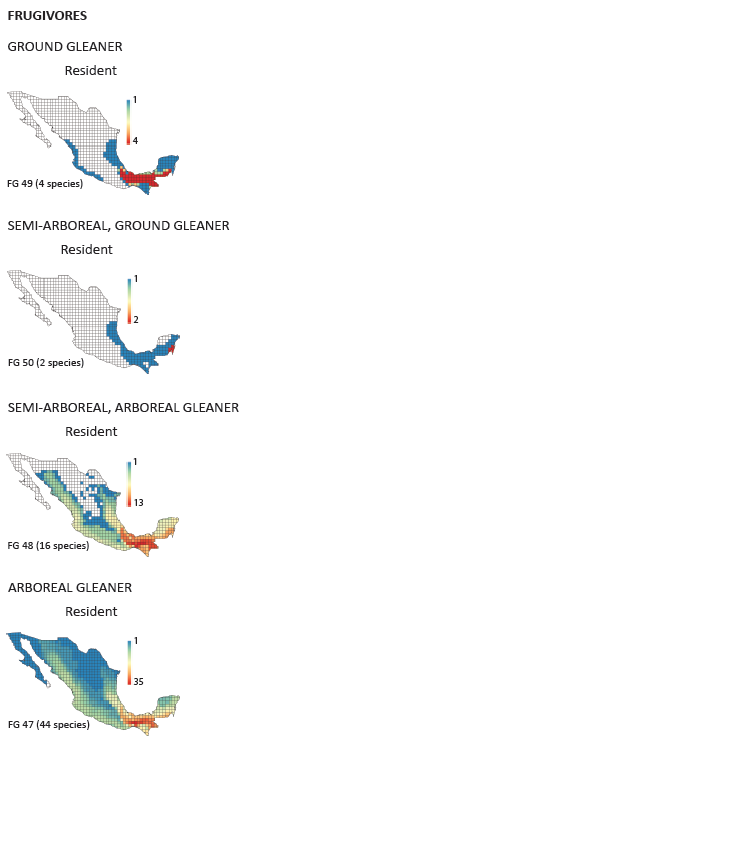


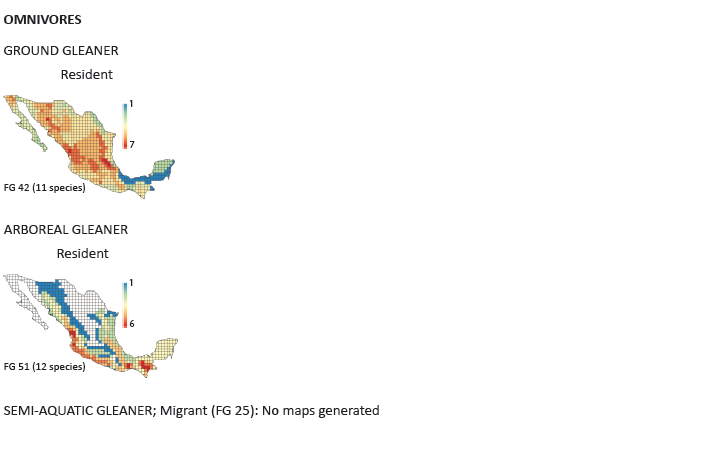


**FUNCTIONAL GROUPS OF MAMMALS**


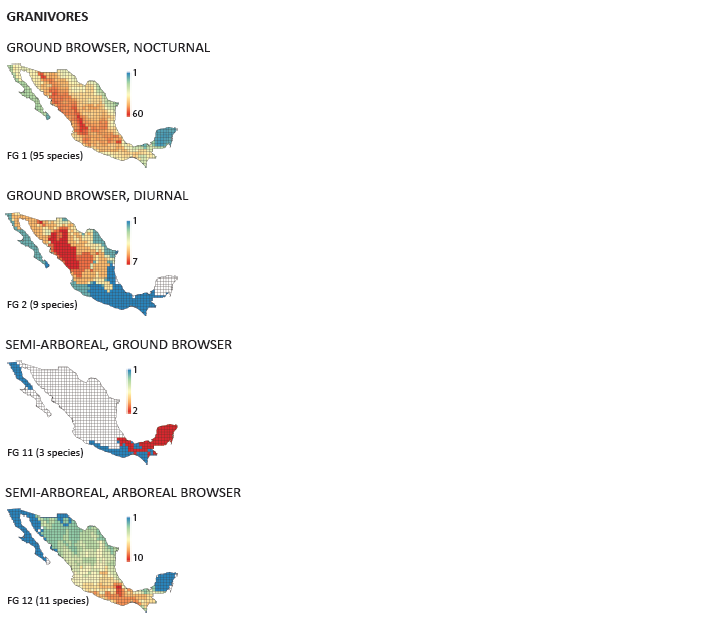


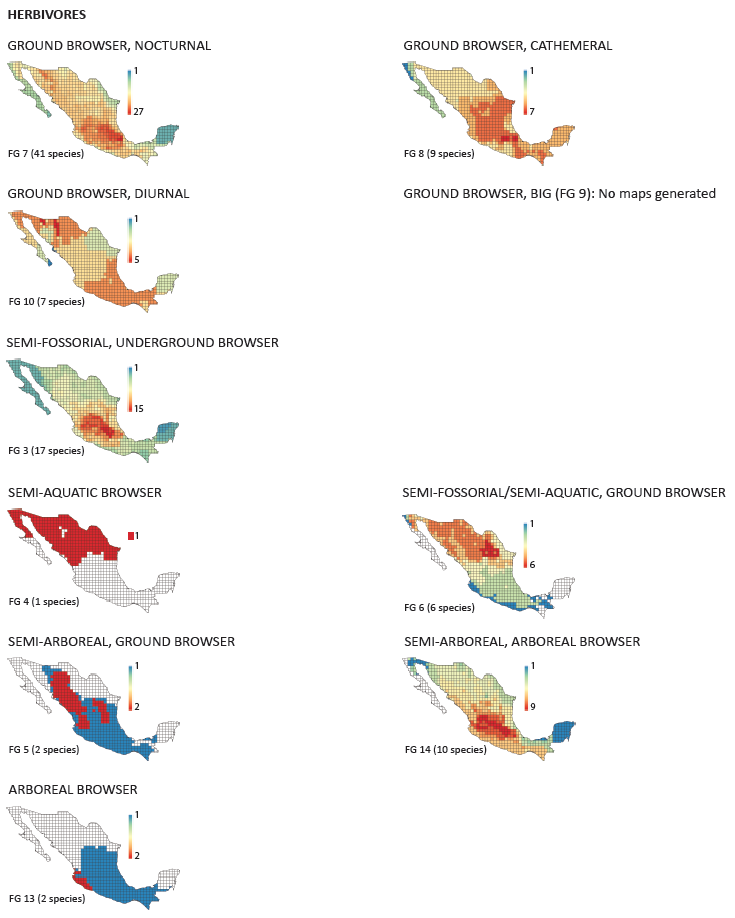


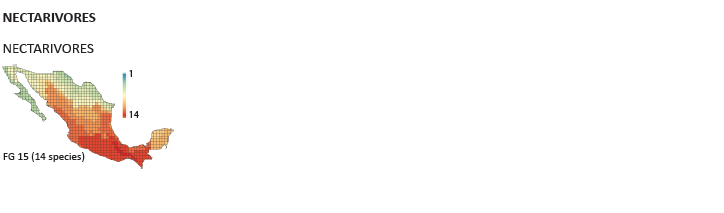


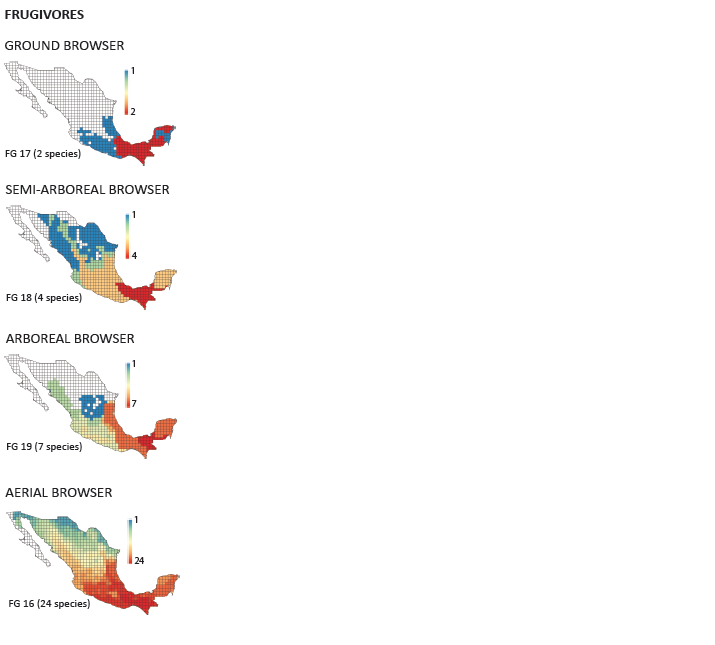


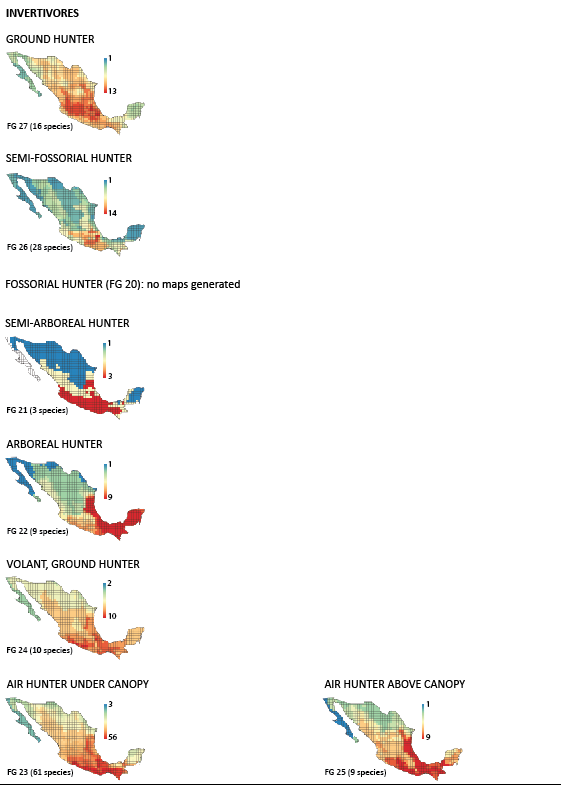


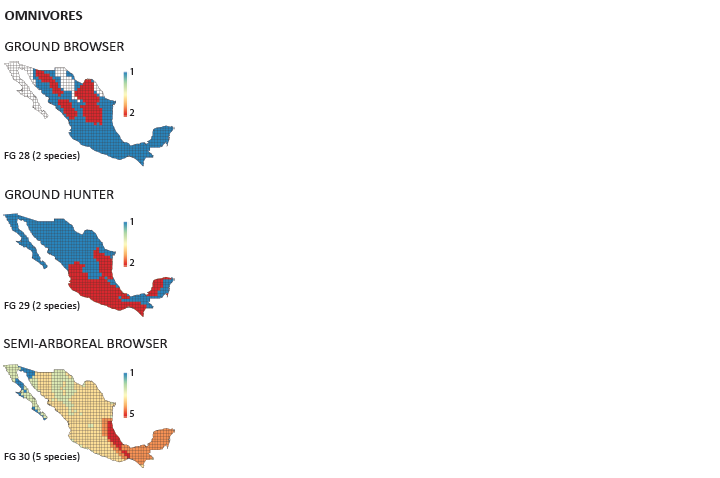


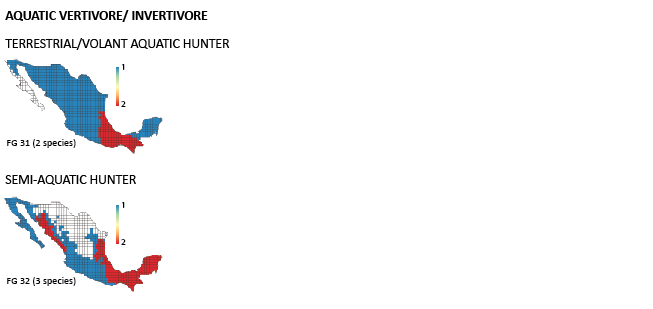


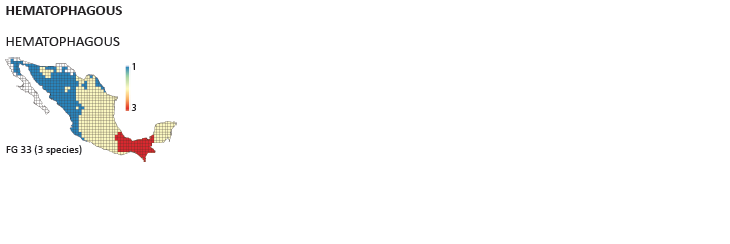


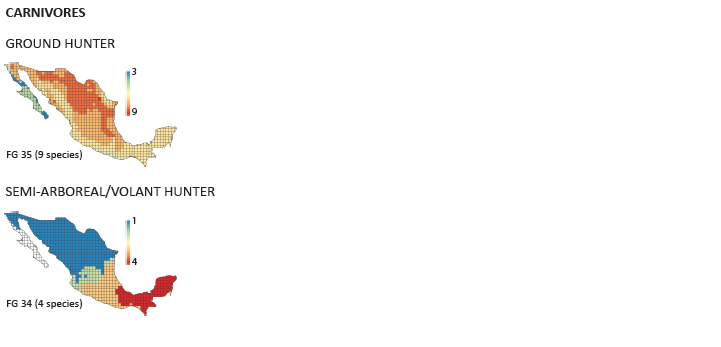


**FUNCTIONAL GROUPS SHARED BY BIRDS AND MAMMALS**


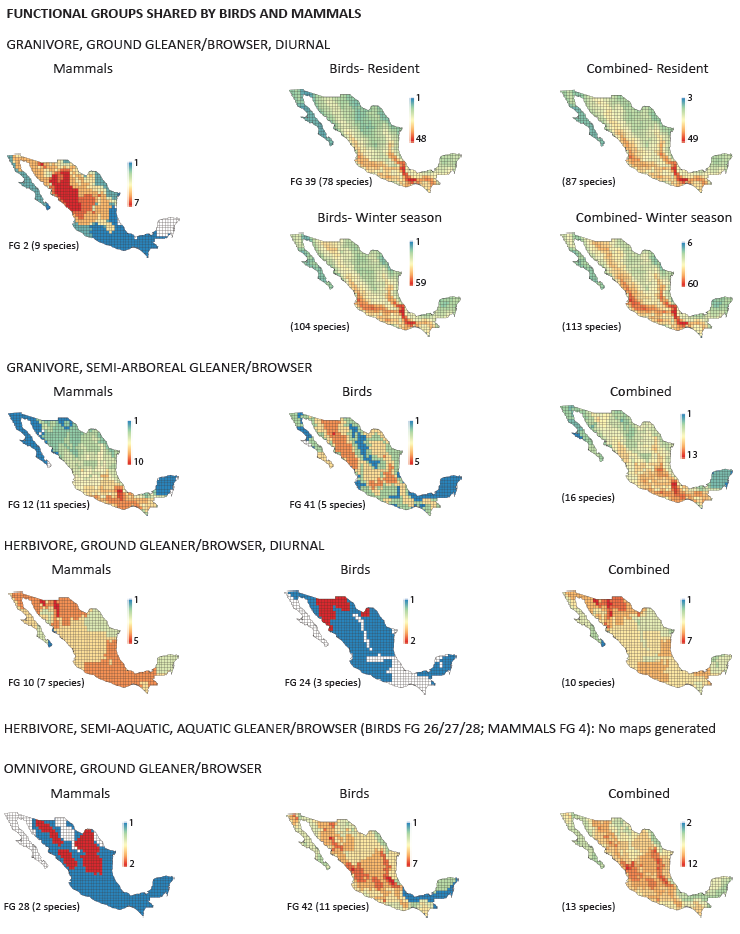


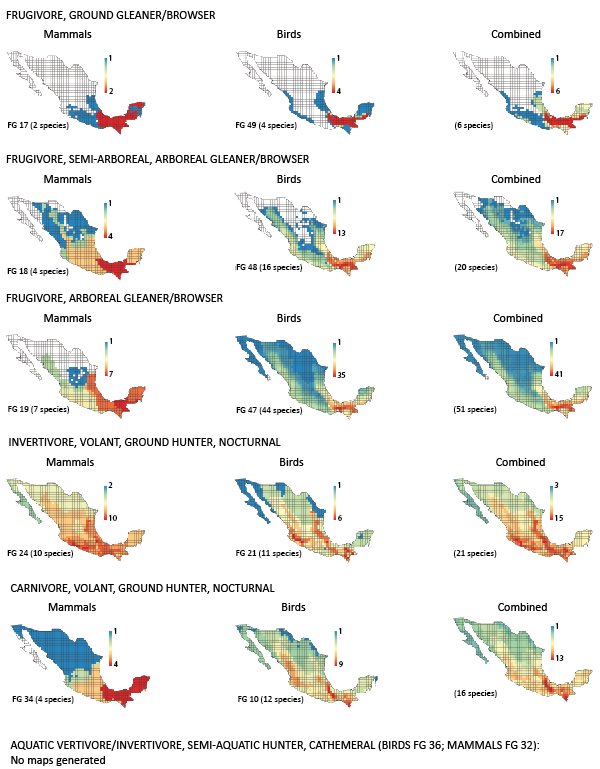

Supplement: S1 File — (DOCX) [file pone.0287036.s004.docx]
